# Plasma protein biomarker model for screening Alzheimer disease using multiple reaction monitoring-mass spectrometry

**DOI:** 10.1038/s41598-022-05384-8

**Published:** 2022-01-24

**Authors:** Yeongshin Kim, Jaenyeon Kim, Minsoo Son, Jihyeon Lee, Injoon Yeo, Kyu Yeong Choi, Hoowon Kim, Byeong C. Kim, Kun Ho Lee, Youngsoo Kim

**Affiliations:** 1 Interdisciplinary Program of Bioengineering, Seoul National University College of Engineering, Seoul, Republic of Korea; 2 Department of Biomedical Engineering, Seoul National University College of Medicine, 28 Yongon-Dong, Chongno-Ku, Seoul, 110-799 Republic of Korea; 3 Gwangju Alzheimer’s Disease and Related Dementia Cohort Research Center and Department of Biomedical Science, Chosun University, Gwangju, 61452 Republic of Korea; 4 Department of Neurology, Chosun University Hospital, Gwangju, 61452 Republic of Korea; 5 Department of Neurology, Chonnam National University Medical School, Gwangju, 61469 Republic of Korea; 6 Department of Biomedical Science, Chosun University, Gwangju, 61452 Republic of Korea; 7 Aging Neuroscience Research Group, Korea Brain Research Institute, Daegu, 41062 Republic of Korea

**Keywords:** Proteomics, Diagnostic markers

## Abstract

Alzheimer disease (AD) is a leading cause of dementia that has gained prominence in our aging society. Yet, the complexity of diagnosing AD and measuring its invasiveness poses an obstacle. To this end, blood-based biomarkers could mitigate the inconveniences that impede an accurate diagnosis. We developed models to diagnose AD and measure the severity of neurocognitive impairment using blood protein biomarkers. Multiple reaction monitoring–mass spectrometry, a highly selective and sensitive approach for quantifying targeted proteins in samples, was used to analyze blood samples from 4 AD groups: cognitive normal control, asymptomatic AD, prodromal AD), and AD dementia. Multimarker models were developed using 10 protein biomarkers and apolipoprotein E genotypes for amyloid beta and 10 biomarkers with Korean Mini-Mental Status Examination (K-MMSE) score for predicting Alzheimer disease progression. The accuracies for the AD classification model and AD progression monitoring model were 84.9% (95% CI 82.8 to 87.0) and 79.1% (95% CI 77.8 to 80.5), respectively. The models were more accurate in diagnosing AD, compared with single APOE genotypes and the K-MMSE score. Our study demonstrates the possibility of predicting AD with high accuracy by blood biomarker analysis as an alternative method of screening for AD.

## Introduction

Alzheimer disease (AD), characterized by tauopathy, amyloid beta (A*β*) plaque formation, and cognitive impairment, is the leading cause of neurodegenerative dementia and the sixth-most frequent cause of death in the US^1,2^. Despite numerous attempts, Food and Drug Administration (FDA)-approved treatments for AD remain undeveloped. Consequently, early diagnosis and monitoring are crucial for improving the prognosis of AD patients^2^.

Diagnosing AD at an early stage is difficult—especially asymptomatic AD, because such patients exhibit a normal cognitive state even after plaque deposition^3^. As damage to neurons accumulates and cognitive impairment worsens, asymptomatic AD progresses to AD with mild cognitive impairment (MCI) (prodromal AD) and AD with dementia. Thus, diagnosing AD in the asymptomatic stage is needed to slow the progression of cognitive impairment^2^.

AD is diagnosed using cognitive assessments and several tests, including genetic tests, biomarker tests, and imaging per clinical criteria^4,5^. In addition, several tests are required to identify the cause of cognitive impairment or dementia, because symptoms can arise from other neurodegenerative diseases. AD biomarkers are also measured to confirm a diagnosis of AD^4,6^. These tests can pose a burden to potential AD patients and are perhaps unsuitable for continuous screening. Among cognitive tests, the Mini-Mental State Examination (MMSE) is widely used^7^, but it has several limitations, such as its vulnerability to illiteracy and association with education level^8^. Other questionnaire-based cognitive tests, such as the Mini-Cog and Montreal Cognitive Assessment (MoCA), have different administration times and can lead to nonuniform results^4,7^. Thus, various approaches are encouraged to determine cognitive states that free from such effects.

Cerebrospinal fluid (CSF) is the most widely used type of sample for assessing levels of biomarkers, such as amyloid beta 1–42 (A*β*42) and tau proteins^9^. However, obtaining CSF is invasive, expensive, and difficult to perform periodically. Because continuous monitoring of its progress is recommended for AD, routine surveillance using blood biomarkers is needed. Because patients with AD have a damaged blood–brain barrier (BBB), their blood can reflect the state of the disease, based on proteins that have passed the disrupted BBB^10,11^.

A*β*42 and tau proteins are well-known biomarkers of AD that are accurate in diagnosing it^12^. Although these biomarkers have been used in routine clinical procedures, they have several limitations. Because A*β*42 and phosphorylated tau (p-tau) exist at subnanogram levels in 1 ml of human plasma, antibody enrichment is required to quantify them, introducing variation in testing and increasing the cost^13,14^. Also, quantifying tau proteins is difficult, because they exist as 6 isoforms with varying post-translational modifications^15^. Further, A*β*42 and t-tau are elevated in other diseases^16,17^, and additional biomarkers are needed to monitor AD owing to the complexity of the causes of AD such as neurodegeneration due to aging, lifestyle, and genetic factors^18^. In this regard, combining biomarkers into a multimarker model would be helpful in diagnosing AD more accurately.

In this study, we analyzed potential biomarkers in plasma from various stages of AD that reflect different severity of neurocognitive stages, and developed multimarker models for monitoring the progression of AD by multiple reaction monitoring-mass spectrometry (MRM-MS). MRM-MS quantifies several target proteins in complex samples simultaneously without antibodies^19,20^, generating results with high reproducibility and consistency between laboratories^21^. Samples from cognitive normal control (CN), asymptomatic Alzheimer disease (AsymAD), prodromal Alzheimer disease (ProdAD), and Alzheimer disease dementia (ADD) patients were analyzed. Our multimarker models, combining proteins with simple AD tests (K-MMSE and apolipoprotein E ɛ4 carrier), showed improved specificity in differentiating AD patients from the control group and evaluating disease progression. Models in this study demonstrate the possibility of diagnosing and monitoring AD more easily with greater discriminatory power.

## Results

### Characteristics of the study populations

**Figure 1:**
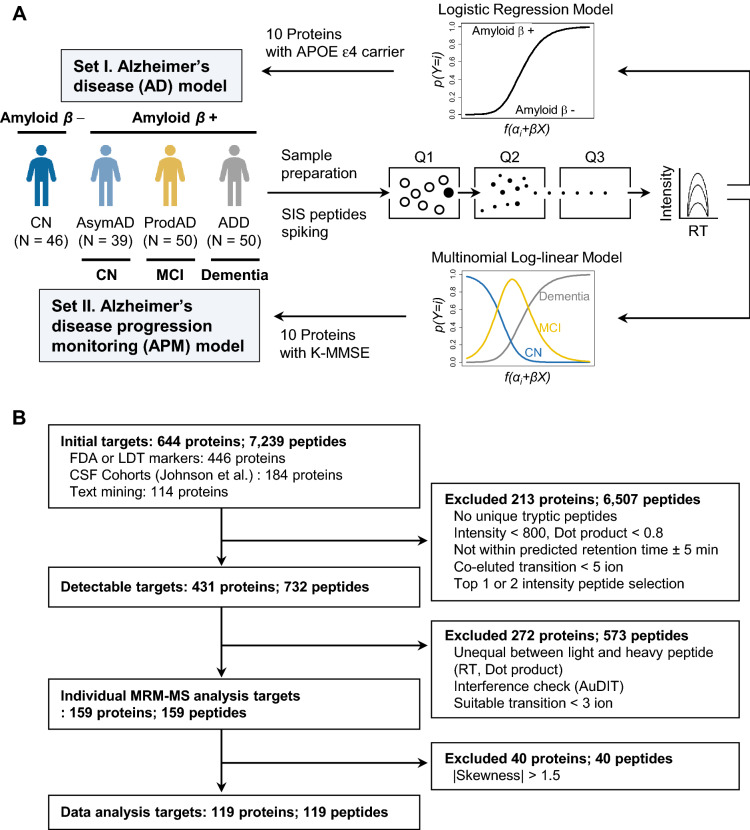
Sample and target protein selection. (**a**) Schematic workflow for developing AD classification model using multiple reaction monitoring-mass spectrometry and multinomial log-linear regression. 10 proteins and APOE genotyping results were selected as features for the Alzheimer disease model, and 10 proteins and K-MMSE scores were used for construction of the Alzheimer disease progression monitoring model. (**b**) Target protein selection workflow. A total of 644 proteins were compiled from three types of resources; 431 proteins that were detectable by MRM-MS analysis were selected. Quality of quantitation was assessed by evaluating the interference from SIS peptides. Ultimately, 159 peptides representing 159 proteins were selected for individual sample analysis, of which 119 were selected for data analysis, based on the skewness of the data.

A total of 185 samples were selected to measure plasma protein levels for developing a model for classifying AD (Fig. 1A). The patients were categorized into 4 groups, based on a diagnosis of dementia, cognitive impairment status, and amyloid PET: (A) cognitive normal control with negative amyloid beta result (CN; N = 46); (B) asymptomatic AD, defined as cognitively normal but positive for amyloid beta (AsymAD; N = 39): (C) prodromal AD group, which showed mild cognitive impairment and positive amyloid beta results (ProdAD; N = 50); and (D) AD dementia, comprising patients with dementia due to AD with amyloid-positive results (ADD; N = 50).

The demographics and clinical characteristics of the 185 patients are summarized in Table 1. The average age was 71.1 years (95% CI 69.4 to 72.9) for the “CN” group, 73.1 years (95% CI 71.3 to 74.9) for the “AsymAD” group, 74.4 years (95% CI 72.8 to 75.9) in the “ProdAD” group, and 73.1 years (95% CI 71.0 to 75.2) in the “ADD” group. The average education level of the “ADD” group was 7.3 years (95% CI 6.0 to 8.6), which was significantly lower than that in other groups (Bonferroni post hoc test, *P* = 0.028 for “CN” group, *P* = 5.1e−4 for “AsymAD” group, and *P* = 5.3e−4 for “ProdAD” group). K-MMSE scores differed significantly between the 4 groups (ANOVA, *P* = 3.4e−27). The percentage of APOE ɛ4 carriers was 19.6% for the “CN” group, 71.8% for the “AsymAD” group, 62.0% for the “ProdAD” group, and 58.0% in the “ADD” group (χ^2^ test, *P* = 3.0e−6). A*β* positivity was evaluated using the PET-BAPL scores of 185 subjects. The percentage of A*β* positivity was 100% for the “AsymAD,” “ProdAD,” and “ADD” groups, and there were no A*β*-positive subjects in the “CN” group.

**Table 1:** Demographics of the three Alzheimer disease groups.

Descriptions		CN, N = 46		AsymAD, N = 39		ProdAD, N = 50		ADD, N = 50		*P* value
		Number of patients (Percent)								
Gender	Female	25	54.3%	21	53.8%	16	32.0%	27	54.0%	0.068
	Male	21	45.7%	18	46.2%	34	68.0%	23	46.0%	
		Mean (95% CI)								
Age, yrs		71.1	(69.4–72.9)	73.1	(71.3–74.9)	74.4	(72.8–75.9)	73.1	(71.0–75.2)	0.091
Education years, yrs		10.1	(8.6–11.5)	11.4	(9.9–12.8)	11.1	(9.7–12.5)	7.3	(6.0–8.6)	< 0.0001****
K-MMSE^1^		26.8	(26.2–27.5)	27.6	(27.0–28.2)	25.3	(24.3–26.2)	18.0	(16.3–19.8)	< 0.0001****
		Number of patients (Percent)								
PET-BAPL	1	46	100.0%	0	0.0%	0	0.0%	0	0.0%	< 0.0001****
	2	0	0.0%	18	46.2%	13	26.0%	7	14.0%	
	3	0	0.0%	21	53.8%	37	74.0%	43	86.0%	
APOE genotype	ɛ2/ɛ2	2	4.3%	0	0.0%	0	0.0%	0	0.0%	0.0003***
	ɛ2/ɛ3	3	6.5%	0	0.0%	2	4.0%	0	0.0%	
	ɛ3/ɛ3	32	69.6%	11	28.2%	17	34.0%	21	42.0%	
	ɛ2/ɛ4	1	2.2%	1	2.6%	0	0.0%	3	6.0%	
	ɛ3/ɛ4	7	15.2%	25	64.1%	26	52.0%	24	48.0%	
	ɛ4/ɛ4	1	2.2%	2	5.1%	5	10.0%	2	4.0%	

^1^Higher score represents better cognitive function; Significance level is represented as “***” for *P* < 0.001 and “****” for *P* < 0.0001; Cognitive normal, CN; Asymptomatic Alzheimer disease, AsymAD; Prodromal Alzheimer disease, ProdAD; Alzheimer disease dementia, ADD.

### Target protein selection for quantitation

Highly quantitative MRM-MS assays for protein biomarkers were developed, as shown in Fig. 1B. To identify proteins with expression patterns that correlated with AD, 644 proteins were selected from the following sources: (1) compiled lists from various sources, including US Food and Drug Administration (FDA)-approved biomarkers, laboratory developed tests (LDT) from the Clinical Laboratory Improvement Amendments (CLIA) database (https://www.accessdata.fda.gov/scripts/cdrh/cfdocs/cfCLIA%20/search.cfm), Title 21 of the Electronic Code of Federal Regulations (eCFR) (https://www.ecfr.gov/cgibin/ECFR?page=browse), and Lab Tests Online (https://labtestsonline.org/tests-index); (2) targets that were differentially expressed in 2 cerebrospinal fluid AD cohorts from Johnson et al.^22^; and (3) a list of candidates from text mining biomarker studies on AD (Supplementary Table S1).

Targets were considered as detectable in plasma if (1) the peak intensity is greater than 800, (2) the elution profile and ratios of transitions were similar to the spectral library, and (3) peptides were unique tryptic peptides. 431 of 644 proteins were detectable in pooled plasma, and stable isotope labeled-standard (SIS) peptides were synthesized for 732 tryptic peptides coming from 431 detectable proteins. To quantify proteins in plasma samples, we selected the 159 most quantifiable peptides, representing 159 proteins. The best peptide that were interference-free, with highest intensity, and showed equality between endogenous and standard peptides were selected for each protein. 40 of 159 proteins were excluded due to high skewness (> 1.5 or < − 1.5). Details of the 119 quantitative proteins for quality control are summarized in Supplementary Table S2, Supplementary Fig. S1, and Supplementary Methods. No batch effect for the MRM-MS analysis of 185 samples was observed in the analytical batches (ANOVA, *P* = 0.116), AD groups (ANOVA, *P* = 0.815), or positron emission tomography-brain amyloid plaque load (PET-BAPL) groups (ANOVA, *P* = 0.630), based on their equivalent distribution (Supplementary Fig. S2).

### Development of model for Alzheimer disease classification

**Figure 2:**
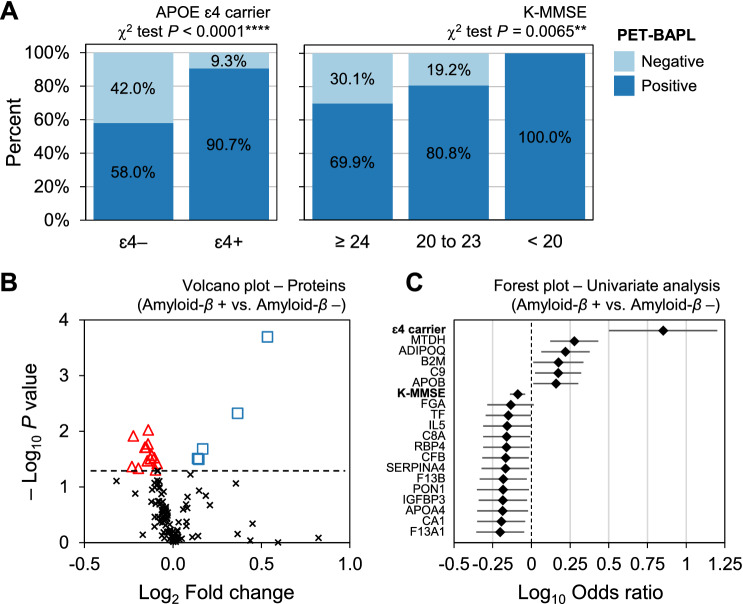
Identification of differentially expressed proteins between PET-BAPL groups. (**a**) PET-BAPL score proportion in APOE ɛ4 carriers and the K-MMSE group, respectively. (**b**) Volcano plot of differentially expressed proteins between PET-BAPL positive and negative groups. Eighteen proteins, with *t*-test *P* values lower than 0.05, are highlighted in red or blue. (**c**) Forest plot of univariate logistic regression analysis of 18 proteins, APOE genotyping results, and K-MMSE scores.

Positron emission tomography-brain amyloid plaque load (PET-BAPL) score, representing the level of deposition of amyloid-*β* (A*β*) in patients’ brains, correlated well with AD-related factors in our study set (Fig. 2A). Most A*β*-positive patients were APOE ɛ4 carriers (χ^2^ test, *P* < 1.0e−4) and had lower K-MMSE scores (χ^2^ test, *P* = 0.0065). To discover potential AD biomarkers, we compared PET-BAPL-positive (A*β*+) and -negative (A*β*−) groups regarding the expression of 119 proteins, of which 18 showed significant differential expression between groups (Fig. 2B, Supplementary Fig. S3, and Supplementary Table S3).

Univariate analysis was performed for the 18 proteins, APOE genotypes, and K-MMSE scores (Fig. 2C). Five proteins (MTDH, ADIPOQ, B2M, C9, and APOB) correlated positively with A*β* positivity, and 13 (FGA, TF, IL5, C8A, RBP4, CFB, SERPINA4, F13B, PON1, IGFBP3, APOA4, CA1, and F13A1) had a negative association. APOE genotype had a stronger association with the A*β* PET results than other protein biomarkers. In contrast, K-MMSE score contributed less to the classification of AD than the protein biomarkers. Eighteen proteins were used to develop a logistic regression model for classifying the A*β*-negative (CN; N = 46) and A*β*-positive AD groups (AsymAD, ProdAD, and ADD; N = 139).

**Figure 3:**
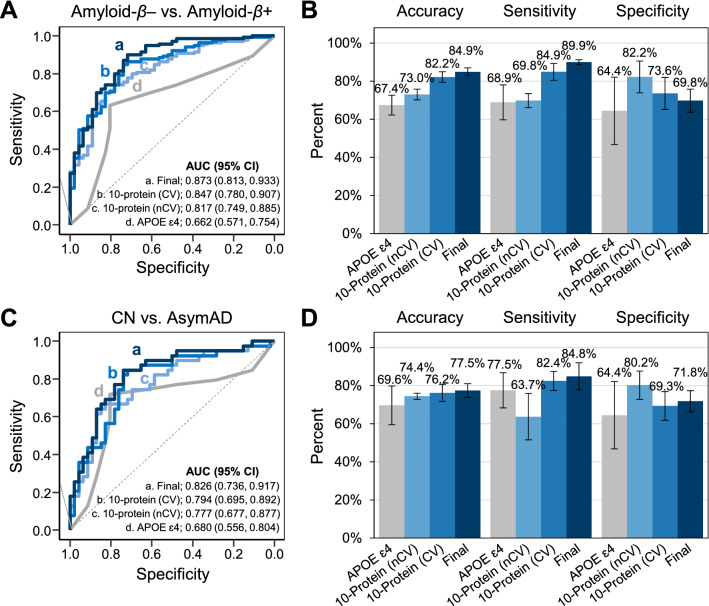
Protein model combined with clinical value for predicting amyloid beta-positive and -negative groups. (**a**) ROC curves for 10 proteins performed with nested crossvadation, 10 proteins performed with fivefold crossvalidation, APOE ɛ4 carriers, and final AD model (all combined) predicting amyloid *β*-positive and -negative groups. Area under the curve (AUC) and 95% CI of AUC values for each model are shown. (**b**) Sensitivity and specificity plots for the 4 models predicting amyloid *β*-positive and -negative groups. Error bars represent 95% confidence interval. (**c**) ROC curves of 10 proteins performed with nested crossvadation, 10 proteins performed with fivefold crossvalidation, APOE ɛ4 carriers, and final AD model (all combined) predicting “CN” and “AsymAD” groups. AUC value and 95% CI of AUC values for each model are shown. (**d**) Sensitivity and specificity plots for the 4 models predicting “CN” and “AsymAD” groups. Error bars represent 95% confidence interval.

Applying a recursive feature elimination (RFE) strategy using nested crossvalidation (nCV), 10 of 18 proteins (MTDH, ADIPOQ, APOB, TF, CA1, C9, APOA4, RBP4, F13A1, and FGA) were selected to develop an 10-protein model. Nested CV was performed with 5 outer folds to test the performance of the model, with 5 inner folds from each outer fold for feature selection and model validation in the training model. To validate the performance of the final model, termed the Alzheimer disease (AD) model, and demonstrate improvement in the performance of the multimarker panel compared with the single test, we compared the 10-protein model with the APOE ɛ4 carrier model (Fig. 3A), which had AUC values of 0.817 (95% CI 0.749 to 0.855) and 0.662 (95% CI 0.651 to 0.754), respectively. The balanced accuracy values of the 10-protein and APOE ɛ4 carrier models were 76.0% (95% CI 88.9 to 83.1) and 66.7% (95% CI 58.9 to 74.5), respectively. The final AD model, combining the 10-protein and APOE ɛ4 carrier models, had an AUC value of 0.873 (95% CI 0.813 to 0.933) and a balanced accuracy value of 79.9% (95% CI 73.2 to 86.6) and improved its sensitivity to 89.9% (95% CI 88.6 to 91.3), higher than that of the other models (Fig. 3B).

We also tested the APOE ɛ4 carrier, 10-protein, and final AD models to classify the “AsymAD” group among normal cognition groups (Fig. 3C). The 10-protein and APOE ɛ4 carrier models had AUC values of 0.777 (95% CI 0.677 to 0.877) and 0.680 (95% CI 0.556 to 0.804), respectively; their balanced accuracy values were 71.9% (95% CI 64.4 to 79.4) and 71.0% (95% CI 63.5 to 78.5). The final AD model performed best, based on its AUC of 0.826 (95% CI 0.736 to 0.917), balanced accuracy of 78.3% (95% CI 71.4 to 85.2), and sensitivity of 84.8% (95% CI 77.7 to 92.0) (Fig. 3D). The overall characteristics of the 3 models are summarized in Supplementary Table S5, and the coefficients of the final AD model are listed in Supplementary Table S6. MTDH and FGA contributed most versus the other candidate markers, based the significance of the coefficients (Supplementary Table S6).

### Multiclass model for predicting Alzheimer disease progression

**Figure 4:**
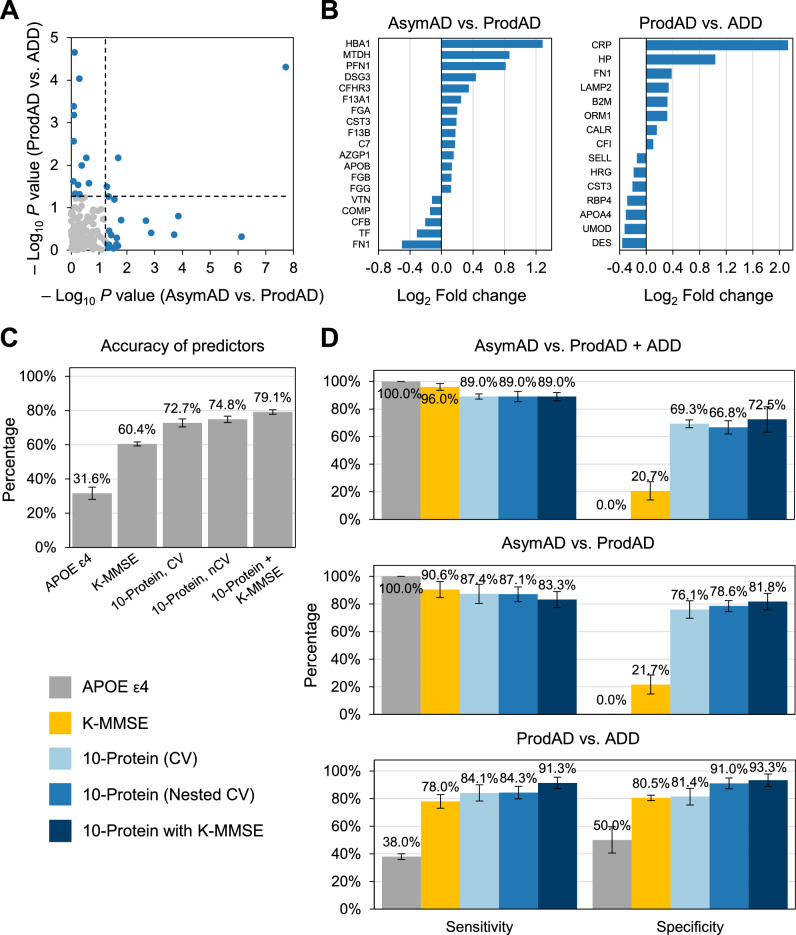
Construction of model for classifying AsymAD, ProdAD, and ADD. (**a**) Scatterplot of *P* values by student’s *t* test between “AsymAD” versus “ProdAD” groups and “ProdAD” versus “ADD” groups. Proteins with *P* values lower than 0.05 are marked as blue dots. (**b**) Fold-change of DEPs in “AsymAD” versus “ProdAD” groups and “ProdAD” versus “ADD” groups. (**c**) Accuracy of APOE ɛ4 carriers, K-MMSE, 10 proteins performed with nested crossvadation, 10 proteins performed with fivefold crossvalidation, and 10-protein with K-MMSE models. (**d**) Bar plots for sensitivity and specificity of APOE ɛ4 carriers, K-MMSE, and final model (10-protein with K-MMSE) in model prediction between “AsymAD” versus “ProdAD” plus “ADD” group, “AsymAD” versus “ProdAD” group, and “ProdAD” versus “ADD” group. Error bars represent 95% confidence interval.

To develop an AD progression monitoring (APM) model for testing the severity of functional degeneration, we identified 32 differentially expressed proteins (DEPs) between 3 representative groups: the “AsymAD” group for cognitively normal AD, the “ProdAD” group with mild cognitive impairment, and the “ADD” group, representing dementia (student’s *t *test, *P* < 0.05) (Fig. 4A and Supplementary Table S7). Of the 19 DEPs between the “AsymAD” and “ProdAD” groups, 14 were upregulated (HBA1, MTDH, PFN1, DSG3, CFHR3, F13A1, FGA, CST3, F13B, C7, AZGP1, APOB, FGB, and FGG) and 5 (FN1, TF, CFB, COMP, and VTN) declined in the “ProdAD” group (Fig. 4B). Of the 15 DEPs between the “ProdAD” and “ADD” groups, 8 were elevated (CRP, HP, FN1, LAMP2, B2M, ORM1, CALR, and CFI) and 7 were lower (DES, UMOD, APOA4, RBP4, CST3, HRG, and SELL) in the “ADD” group.

Nested CV was performed for the multinomial log-linear regression model with 5 outer folds and 5 inner folds for each outer fold. With RFE selection in the nested CV, the 10-protein model (CALR, F13A1, LAMP2, APOA4, B2M, FN1, FGA, ORM1, DES and MTDH) was deemed to have the best accuracy versus other models with differing numbers of variables (Fig. 4C). Notably, 4 of 10 proteins (APOA4, FGA, F13A1, and MTDH) were also included in the final AD model for classifying A*β*-positive AD groups. In the comparison of AsymAD and ProdAD, FN1, FGA, and F13A1 contributed more, based on the significance of the coefficients. In addition, APOA4, B2M, and ORM1 contributed more when differentiating between AsymAD and ADD.

Next, we compared the performance of the 10-protein model with other models that used conventional AD factors (APOE ɛ4 carrier and K-MMSE) (Fig. 4C and Supplementary Fig. S5). The 10-protein model was more accurate [72.7% (95% CI 70.4% to 75.0%)] than the APOE ɛ4 carrier [31.6% (95% CI 28.0% to 35.2%)] and K-MMSE models [60.4% (95% CI 59.3% to 61.6%)]. Further, the final APM model, combining 10 protein variables with K-MMSE, had the highest accuracy [79.1% (95% CI 77.8% to 80.5%)]. It also performed better than other models with conventional factors (Fig. 4D and Supplementary Table S9). The APOE ɛ4 carrier model failed to estimate neurocognitive impairments accurately, based on its zero specificity in comparing “AsymAD” with the “ProdAD” plus “ADD” groups and the “ProdAD” group alone, despite a sensitivity of 100%.

Overall, in the comparisons between AD groups, although K-MMSE had balanced sensitivity and specificity in its classification, the final APM model (10-protein with K-MMSE) had diagnostic power with sensitivity and specificity that exceeded 80%. The coefficients of the final APM model are listed in Supplementary Table S10, and box plots of its 11 features (10 proteins and K-MMSE score) are shown in Supplementary Fig. S5.

### Association of plasma proteins with CSF proteins

**Figure 5:**
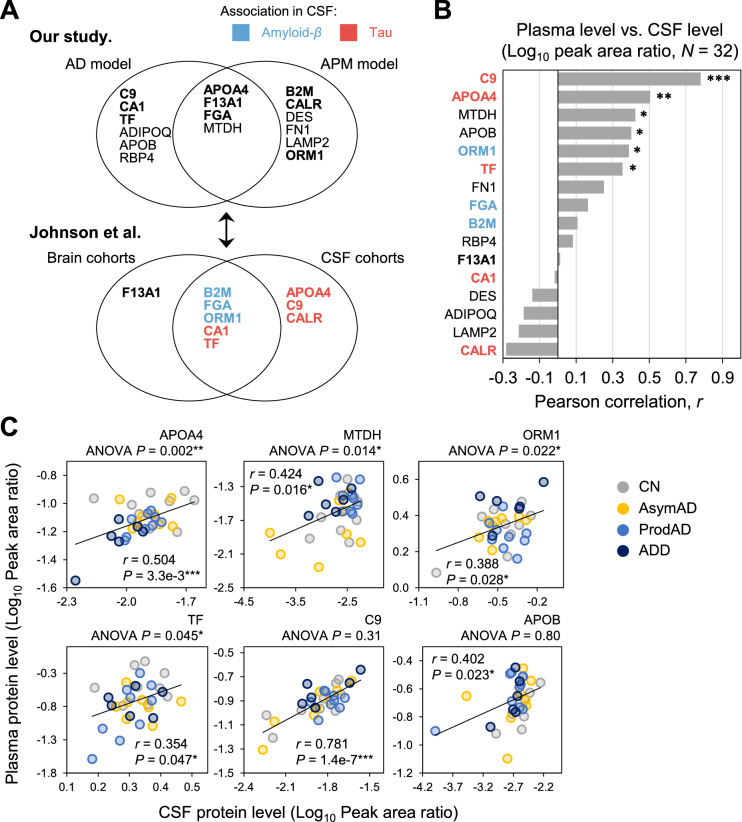
Association of plasma proteins with CSF proteins. (**a**) Correlation between Johnson et al. and our study. Between the AD and APM models, APOA4, F13A, FGA, and MTDH were selected by both as features, whereas C9, CA1, TF, APOB, RBP4, and ADIPOQ were unqiue to the AD model and CALR, B2M, LAMP2, DES, FN1, and ORM1 were unique to the APM model. Among 16 proteins, 9 were mentioned in Johson et al., in which B2M, FGA, and ORM1 were associated with amyloid and APOA4, C9, CALR, CA1, and TF were associated with tau. (**b**) Target correlation between CSF and plasma. Correlation of the 16 proteins among the 32 that matched in both CSF and plasma. Seven targets had Pearson’s correlation values above 0.3. (**c**) Scatter plot of 6 target proteins among the CSF and plasma samples.

To examine whether the proteins in the models were derived from the brain or cerebrospinal fluid (CSF) of AD patients, we compared the proteins in the 2 models with those that Johnson et al.^22^ identified in their brain and CSF cohorts (Fig. 5A). Nine of 16 proteins (56.3%) overlapped with the module proteins in the brain cohorts (6 proteins; B2M, CA1, F13A1, FGA, ORM1, and TF) or the DEPs in the CSF cohorts (8 proteins; APOA4, B2M, CA1, C9, CALR, FGA, ORM1, and TF). Notably, all 8 CSF proteins correlated significantly with amyloid-*β* or tau levels in the CSF (Supplementary Fig. S6).

The CSF levels of 16 proteins were compared with their plasma levels in 32 CSF samples, matched with plasma samples in our study. Six of 16 proteins (37.5%) correlated positively between plasma and CSF levels (Fig. 5B; Pearson’s correlation, *P* < 0.05). Moreover, APOA4, MTDH, ORM1, and TF differed significantly in the CN and 3 AD groups (Fig. 5C; ANOVA, *P* < 0.05).

## Discussion

In this study, potential protein biomarkers for AD were analyzed by MRM-MS. MRM-MS can accurately quantify low-abundance AD biomarkers in blood from early-stage AD patients with superior sensitivity. The high reproducibility, accuracy, and sensitivity of MRM-MS render it suitable for the early diagnosis of AD across institutions^23^. The diagnostic performance of the biomarkers was estimated, and a multimarker model was developed by combining biomarkers to improve accuracy of conventional biomarkers, compared with APOE genotyping and MMSE, which need additional tests for accurately diagnosing AD. Amyloid beta plaque deposition is tested primarily using amyloid PET imaging or CSF assay. Although these tests yield valid results on A*β* deposition status and although their use in measuring biomarkers is mentioned in diagnostic criteria for AD^4^, an A*β*-positive person by PET might need additional tests to verify whether the amyloidosis actually originates from AD or his advanced age^24^. In addition, lumbar puncture, which is needed to obtain CSF samples from patients, is relatively invasive. T hus, a blood biomarker test would be useful in screening for AD if it can be performed prior to PET imaging or CSF assay.

APOE genotyping and MMSE are also used for diagnosing AD and are typically less strenuous for patients. However, according to a meta-analysis, over 40% of non-APOE ε4 carriers are at risk of developing AD^25^. MMSE can give varying results, depending on the education level of the subjects or the recall words that examiners choose^8^. In effect, both APOE ɛ4 carriers and the K-MMSE model classified AD less accurately in our study—for the A*β*-positive versus A*β*-negative groups and the neurocognitive impairment groups.

Further, the final AD and APM models, combining protein biomarkers with conventional AD factors, made much better predictions. These 2 models could complement conventional diagnostic tests and help identify people with AD with high precision while reducing expenses and simplifying the process. Our models could increase the accuracy of predicting AD and reduce potential hazards, lower medical costs, and increase the quality of life for AD patients^26^. In particular, the APM model might also serve as a surrogate biomarker by determining whether neurodegeneration in AD patients improves through medications in clinical trials. Based on recent data that support treatments for mild and moderate AD, AD patients, especially those with MCI, might benefit from our model by identifying their ailment at an earlier stage and preparing for treatment accordingly^26^. To insure integrity of the method, 16 proteins included in the modesl were analyzed for limit of detection (LOD), limit of quantification (LOQ), and stability according to CPTAC guideline (Supplementary Methods). Most of the targets passed the evaluation, whereas CALR and DES failed once under the 24-h condition, DES failed once under the freeze and/ thaw condition, and ADIPOQ failed once in under the 2-time freeze and/ thaw condition, with variability under 30% (Supplymentary Table S12, S13). For future validation assays, precision of the analysis using crude SIS peptides can be compared with results of analysis with highly purified peptides in further studies.

Damaged neurons and synapses are a characteristic of AD patient brains. The toxicity of A*β* causes oxidative damage and synaptic alterations, and synaptic dysfunction is one of the main factors that effect cognitive impairments in AD patients^27^. In our study, beta-2-macroglobulin (B2M), which is associated with neuronal function, such as neurogenesis, and neuronal actin expression, was upregulated with the progression of AD^28,29^. Carbonic anhydrase 1 (CA1) is related to the preservation of pH levels in the brain, protecting CSF and neuronal function, and its levels decrease as more A*β* plaque is deposited^30^. Fibrinogen alpha chain (FGA) interacts with A*β* and can be deposited, leading to plaque-induced inflammation^31^. In addition, A*β* is a cause of inflammation, which damages neurons and aggravates the pathogenesis of AD^32^. Coagulation factor XIII A chain (F13A1) is detected in reactive microglia, which is related to neuroinflammation, and F13A1 is involved in transglutaminase-mediated polymerization of amyloid beta proteins^33^. Alpha-1-acid glycoprotein 1 (ORM1) was enriched in pathways that are associated with inflammation, such as IL-6 signaling, in a profiling study on plasma and brain from AD patients^34^. Transferrin (TF) regulates iron levels by binding to iron and controlling oxygen radical formation^35^.

Further, associations between AD and other proteins in our models have been reported in AD studies on inflammation (C9,CALR)^36^, A*β* plaque formation (APOB, FN1, APOA4, LAMP2)^37–39^, and neuronal and brain damage, such as glutamate accumulation in the synapses (MTDH)^40^ and synaptic plasiticity (ADIPOQ)^41^. C9 is related to complement activation and inflammation. APOB and APOA4 interact with amyloid beta and influence A*β* plaque formation but with opposing effects. APOA4 contributes to the clearance of amyloid beta by binding to amyloid beta and inducing glial cells to take up amyloid beta. APOB also binds to amyloid beta peptide, but its mechanism opposed that of APOA4. APOB has been suggested to have high amyloid-forming potential, and APOB can form amyloid-like structures^42^. FN1 is involved in clotting, which can affect amyloid beta fibrilization^39^. LAMP2 is associated with lysosomal function, and lysosomal dysfunction leads to ROS generation and deposition of wastes that can be neurodegenerative^43^. CALR, which is the receptor for C1q, mediates ROS production by binding to C1q and triggering signaling pathways that are related to ROS^44^. By combining biomarker candidates that represent various pathologies of AD, our AD and APM models can classify the stage of disease with enhanced specificity for AD.

Among the 16 proteins that were used in the final 2 models, 6 (APOA4, B2M, CALR, F13A1, FGA, and TF) were related to AD in the brain and CSF in a cohort study by Johnson et al.^22^; 4 proteins (B2M, FGA, F13A1, and TF) belonged to modules that were related to blood, myelin or oligodendrocyte, and microglia. By Gene Ontology analysis using all proteins in these modules, enriched terms were associated with major AD pathologies (Supplementary Table S11)^22^. Also, CSF levels of 6 proteins (APOA4, B2M, CALR, CST3, FGA, and TF) correlated with AD-related factors, such as cognitive score (MoCA) and CSF markers (A*β*, total tau, and phospho-tau) (Supplementary Fig. S6). Among the 6 proteins, APOA4 and TF were significantly linked to total tau (t-tau), phospho-tau, and MoCA score, except for CALR linked to t-tau and phospho-tau, but not to MoCA score. Conversely, B2M, CST3, and FGA correlated with A*β* but not MoCA score. Changes in A*β* and tau were involved in a process of preclinical AD in a longitudinal study, and exacerbation of tauopathy during disease progression has been suggested to have a more direct effect on cognitive decline by neurodegeneration due to a damaged cytoskeleton^1,45,46^. In addition, A*β* indirectly affects cognition in a tau-mediated manner, wherein changes in A*β* are followed by those in tau, and neurotoxic A*β* plaques cause neurodegeneration by affecting synaptic function^2,45^. Our APM model, constructed with A*β-* or tau-related protein biomarkers, might be a useful tool for monitoring neurodegeneration in AD patients.

There are several limitations of our study. The samples were obtained from ethnic Koreans, which might limit the applicability of the results to the general populace. Thus, prior to clinical use, the model must be tested in other countries and populations of different ethnicities and etiologies to validate its diagnostic power. Also, the model was developed with limited sample size. Small numbers of samples were included in each group; thus, the models were nested cross-validated to avoid overfitting. To check biasness, comparison between K-fold cross validation and nested cross validation were performed. Nevertheless, the model must be validated with a larger number of samples. MCI patients who were negative for amyloid beta were not included in our study. Because tests for filtering patients with impaired cognitive states due to other diseases are suggested in diagnostic guidelines for AD, further studies with such patients are needed to increase the accuracy of the models. Despite these limitations, the improved diagnostic power of our models demonstrate the possibility of diagnosing AD at an early stage by routine blood test. Our multimarker models thus reduce the burden of tests for diagnosing AD and help discriminate AD patients at an early stage, when they need continuous clinical care to prevent disease progression.

## Methods

### Materials

Rapigest™ SF was purchased from Waters (Milford, MA, USA). Neat formic acid (FA), dithiothreitol (DTT), iodoacetamide (IAA), ammonium bicarbonate (ABC), and bicinchoninic acid (BCA) solution were obtained from Sigma-Aldrich (St. Louis, MO, USA). Sequencing-grade modified trypsin was acquired from Promega (Madison, WI, USA). High-performance liquid chromatography (HPLC)-grade water, 0.1% FA in HPLC water, 0.1% FA in acetonitrile (ACN), and methanol were purchased from Thermo Fisher Scientific (Bremen, Germany).

### Study participants and sample collection

A total of 261 patients were prospectively recruited between 2014 and 2019 from the Gwangju Alzheimer’s & Related Dementia (GARD) cohort in Gwangju, Korea. All subjects were examined by experienced neurologists and underwent a full dementia screen, which comprised the Seoul Neuropsychological Screening Battery (SNSB) and Clinical Dementia Rating^47,48^. The SNSB is an overall neuropsychological test for 5 domains of cognition and includes the Korean version of the Mini-Mental State Examination (K-MMSE)^49^. Cognitive impairment was defined as a Z-score lower than -1.5 of the standard deviation (normalized for age, sex, and education) on at least 1 of the neuropsychological tests (memory, language, visuospatial function, attention, or frontal/executive). The clinical diagnosis of dementia and mild cognitive impairment (MCI) was performed per the National Institute on Aging and Alzheimer’s Association (NIA-AA) and International Working Group 2 (IWG-2) workgroups^4,6,50^. The subjects underwent MRI and Florbetaben positron emission tomography (PET) scans to image the brain. Florbetaben PET images for scoring the brain beta-amyloid plaque load (BAPL) were acquired as detailed^51,52^. PET images were acquired 90 min after intravenous injection of 300 MBq ^18^F-Florbetaben using a Discovery ST PET-CT scanner (General Electric Medical Systems, Milwaukee, WI, USA). BAPL scores were measured visually on the PET images: 1 indicated a beta-amyloid-negative PET scan, and 2 and 3 were considered beta-amyloid-positive. Subjects with BAPL scores 2 or 3 were classified as patients having AD.

For genotyping apolipoprotein E (APOE), whole blood was collected in ethylenediaminetetraacetic acid (EDTA) tubes. Peripheral blood leukocytes were isolated from blood, and genomic DNA was extracted from leukocytes. APOE was genotyped using a genomewide genotyping array (Affymetrix Axiom® KORV1.0, Santa Clara, CA, USA) at DNALink (Seoul, South Korea). The Center for Genome Science, Korea National Institute of Health, Republic of Korea (4845-301. 3000-3031) designed and optimized the genotyping array^53^, which included 2 APOE-related small nucleotide polymorphisms (rs7412, rs429358).

A total of 76 of 261 subjects were excluded from the study for the following reasons: dementia or MCI that was unrelated to AD, refusal to participate, failure to be administered amyloid PET tracer, and altered status on re-examination. A total of 185 plasma samples were selected. All 185 subjects were classified into the four groups (46 CN subjects, 39 AsymAD patients, 50 ProdAD patients, and 50 ADD patients) according to A*β* positivity by BAPL scores and clinical criteria by NIA-AA and IWG-2 workgroups.

### Ethical approval and consent to participate

The study was approved by the institutional review board (IRB) of Chosun University Hospital, Korea (IRB approval No. CHOSUN 2013-12-018-070). All research methods were conducted in accordance with the Declaration of Helsinki and relevant guidelines and regulations. Written informed consent was provided by all volunteers and family members or authorized caregivers in the case of cognitively impaired patients.

### Sample preparation

A total of 185 plasma samples were prepared in block-randomized batches with respect to age, gender, and group, using the psych package (Version 1.9.12) in R (Version 4.0.0). Six high-abundance proteins (albumin, immunoglobulin G, immunoglobulin A, haptoglobin, transferrin, and alpha-1-antitrypsin) were depleted on a high-performance liquid chromatography (HPLC) instrument that was coupled to a Multiple Affinity Removal System Human-6 (MARS Hu-6, 4.6 mm × 100 mm, Agilent, CA, USA) (Supplementary Methods). Depleted plasma samples were then concentrated at 3470 g for 6 h at 4 °C using 3000-Da molecular weight cutoff (MWCO) centrifugal filter units (Amicon Ultra-4 3 K, Millipore, MA, USA). After concentration, the protein in the plasma samples was measured using the Pierce™ BCA Protein Assay Kit (Thermo Scientific, Rockford, IL, USA). Then, 20 μL of solution that contained 0.2% RapiGest, 20 mM dithiothreitol (Merck, Darmstadt, Germany), and 100 mM ABC, pH 8.0 was added to 100 μg of protein and incubated at 60 °C for 60 min with shaking for denaturation and reduction. Next, 10 μL 100 mM iodoacetamide (Sigma, MO, USA) was added and incubated at room temperature for 30 min with shaking in the dark to alkylate the proteins. Subsequently, 40 μL 0.1 μg/μL trypsin (sequencing-grade, Promega, WI, USA) in 50 mM ABC solution, pH 8.0 was added and incubated at 37 °C for 4 h with shaking to digest the proteins. The digestion was quenched with 10 μL 10% formic acid at 37 °C for 30 min with shaking.

Quenched samples were centrifuged at 16,602 g and 4 °C for 60 min, and 90 μL of the supernatant was transferred to a clean tube to remove any byproducts of the RapiGest. Digested samples were cleaned using Oasis® HLB (hydrophilic-lipophilic balance) 1 cc (30 mg) extraction cartridges (Waters Corp., MA, USA). Columns were activated with 1 mL 100% methanol and equilibrated with 3 mL 100% acetonitrile and 5 mL 0.1% formic acid in distilled water. Each digested plasma samples was loaded onto the column twice and washed with 3 mL 0.1% formic acid in distilled water. Samples were eluted with 0.5 mL 0.1% formic acid in 40% acetonitrile and 0.5 mL 0.1% formic acid in 60% acetonitrile. Eluates were lyophilized on a vacuum centrifuge and resolubilized to 0.25 μg/μL in 0.1% formic acid/water.

### Liquid chromatography-tandem mass spectrometry

The plasma samples were analyzed on an Agilent 6490 triple quadrupole (QQQ) mass spectrometer (Agilent, CA, USA) with a Jetstream electrospray source, coupled to a 1260 Infinity HPLC system (Agilent, CA, USA). Buffers that consisted of 0.1% formic acid/water (v/v) and 0.1% formic acid/acetonitrile (v/v) were used as mobile phases A and B during the mass analysis. A reversed-phase analytical column (150 mm × 0.5 mm id, Agilent Zorbax SB-C18, 3.5-μm particle size) was used with mobile phases A and B to separate the peptides. In every analysis between batches, LC quality control and a suitability test were performed using 6 × 5 LC–MS/MS Peptide Reference Mix from Promega (Madison, WI, USA).

The multiple reaction monitoring-mass spectrometry (MRM-MS) analyses were performed in positive mode. A total of 10 μL of sample was injected to the mass spectrometer for each analysis. The total run time of LC was 70 min, and the specific LC buffer system was as follows: The flow rate of the mobile phases was 40 μL/min. The LC gradient started at 97% mobile phase A and 3% mobile phase B. The gradient of mobile phase B was increased linearly from 3 to 40% and flowed for 52 min for separation of elution of the peptides. The buffer with 60% mobile phase B flowed for 3 min, and the column was equilibrated for the next run for 15 min with the buffer with 3% mobile phase B. The ion spray capillary voltage was 2500 V, and the nozzle voltage was set to 2000 V. The cell accelerator voltage was 5 V, the delta electron multiplier voltage (EMV) was adjusted to 200 V, and the fragment voltage was set to 380 V. The temperature of the drying gas was set to 250 °C at 15 L/min, and the sheath gas was adjusted to 350 °C at a flow rate of 12 L/min.

### Proteomic data processing and statistical analysis

Raw MRM-MS data were processed in Skyline (MacCoss Lab, University of Washington, Seattle, WA) to compute the peak areas of the transitions. The peak areas of endogenous peptides were normalized to those of their corresponding stable isotope-labeled standard (SIS) peptides to compare the relative abundance of candidate peptides between samples. SIS peptides were synthesized with lysine or arginine that were heavy isotope-labeled (^13^C_6_^15^N_2_ or ^13^C_6_^15^N_4_) at the C-termini. Targets with interference signals were excluded, per the Automated Detection of Inaccurate and imprecise Transitions (AuDIT) algorithm^54^. The log_10_-transformed, protein-wise centered and scaled peak area ratios were used for data processing. Interference-free check and quality control for individual analysis are detailed in Supplementary Methods.

Differences between patient groups were determined by student’s *t* test for bivariate and analysis of variance (ANOVA) for multiple groups using SPSS (versions 23.0 and 25.0; IBM, Chicago, IL), and unadjusted *P* values were used to save potential candidate biomarkers and minimize type II errors^55^. Pearson’s correlation and χ^2^ test were performed to verify the association between protein level and clinical information. The skewness of the log-transformed protein level was assessed using the e1071 package (version 1.7–3) in R. Logistic regression was perfomed to develop a binary classification model using the ‘glm’ function in the caret package (version 6.0–86). To develop a multiclass classification model, multinomial log-linear regression was performed using the ‘multinom’ function in the nnet (version 7.3–14) and caret packages (version 6.0–86) in R. Both models were validated using nested crossvalidation (5 outer folds and 5 inner folds for each outer fold) and fivefold cross validation^56,57^. The associations of the 16 proteins in the AD model for detecting AD and the APM model for monitoring the progression of AD with biological functions that were related to the progression of AD were characterized using protein lists of postmortem brain network modules and published proteomic data on CSF cohorts from Johnson et al.^22^. Functional annotations of proteins that were enriched in each risk subgroup were identified using the Database for Annotation, Visualization and Integrated Discovery (DAVID) Bioinformatics Resources (Version 6.8)^58^.

## Supplementary Information

Supplementary Information 1.

Supplementary Information 2.

Supplementary Information 3.

## Data Availability

The raw MRM-MS files for all 185 plasma samples were deposited into PeptideAtlas with the quantitation target lists (Dataset identifier: PASS01631; Password: SH6233b). The quantitation information were also deposited to Panorama Public repository (https://panoramaweb.org/CXlSFG.url). Clinical information and cleaned expression data from Johnson et al. that were used to characterize the associations of the 17 proteins in the AD and APM models were downloaded from the “Synapse” data portal (https://www.synapse.org/consensus; Synapse ID: syn20933797).
