# Supplementary material for: Plasma protein biomarker model for screening Alzheimer disease using multiple reaction monitoring-mass spectrometry
Source: Sci Rep. 2022 Jan 24;12:1282. doi: 10.1038/s41598-022-05384-8 (PMC8786819; doi:10.1038/s41598-022-05384-8)
Supplement: Supplementary file 1 — Supplementary Information 1. [file 41598_2022_5384_MOESM1_ESM.pdf]

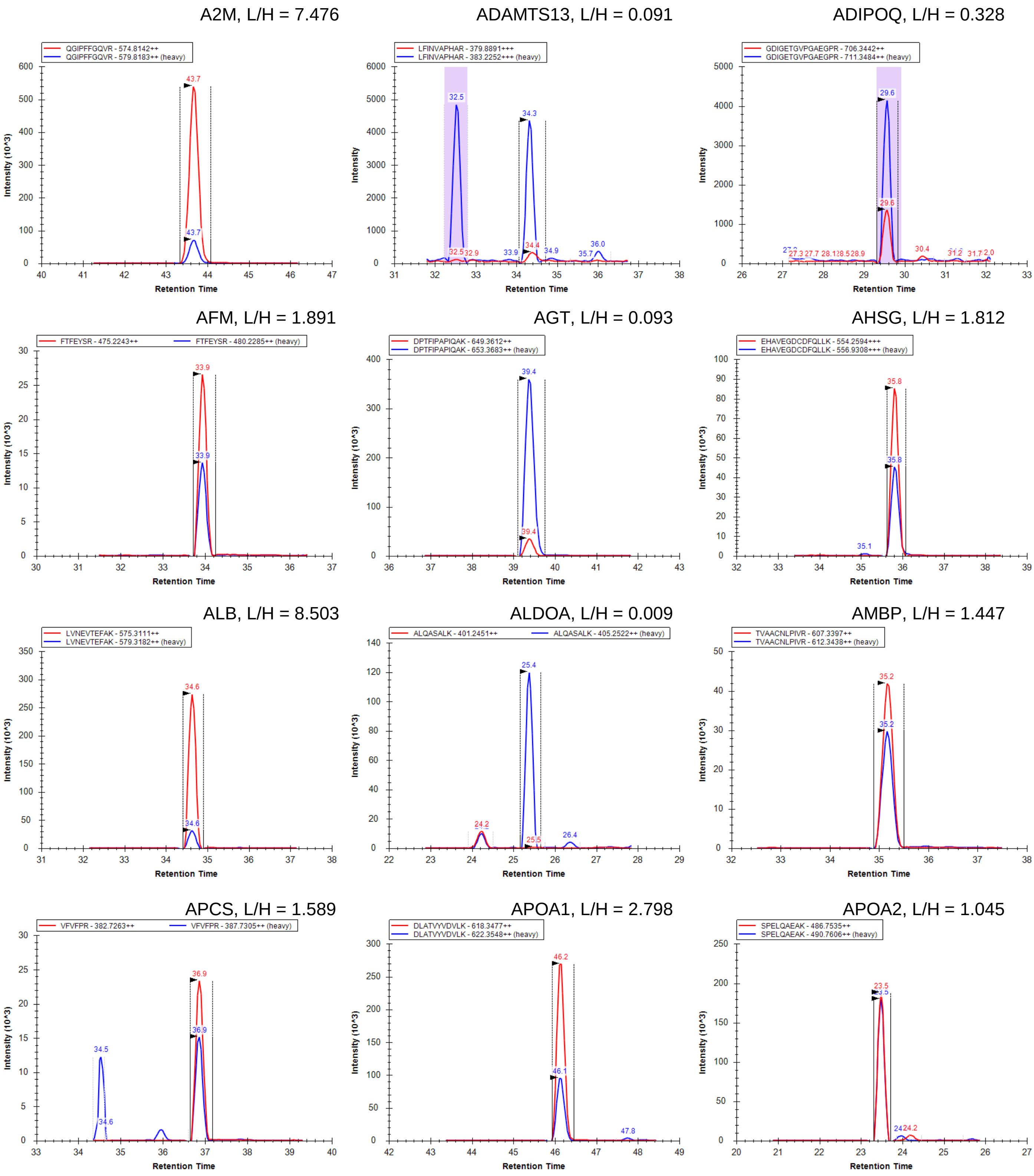

Supplementary Figure S1. Extracted Ion Chromatograms (XIC) of examples of light-to-heavy peptide peak area ratios of 119 proteins. Light-to-heavy peak area ratio values are shown in the upper right of the chromatograms. The chromatograms of light peptides are red, whereas chromatograms of the corresponding heavy peptides are blue.

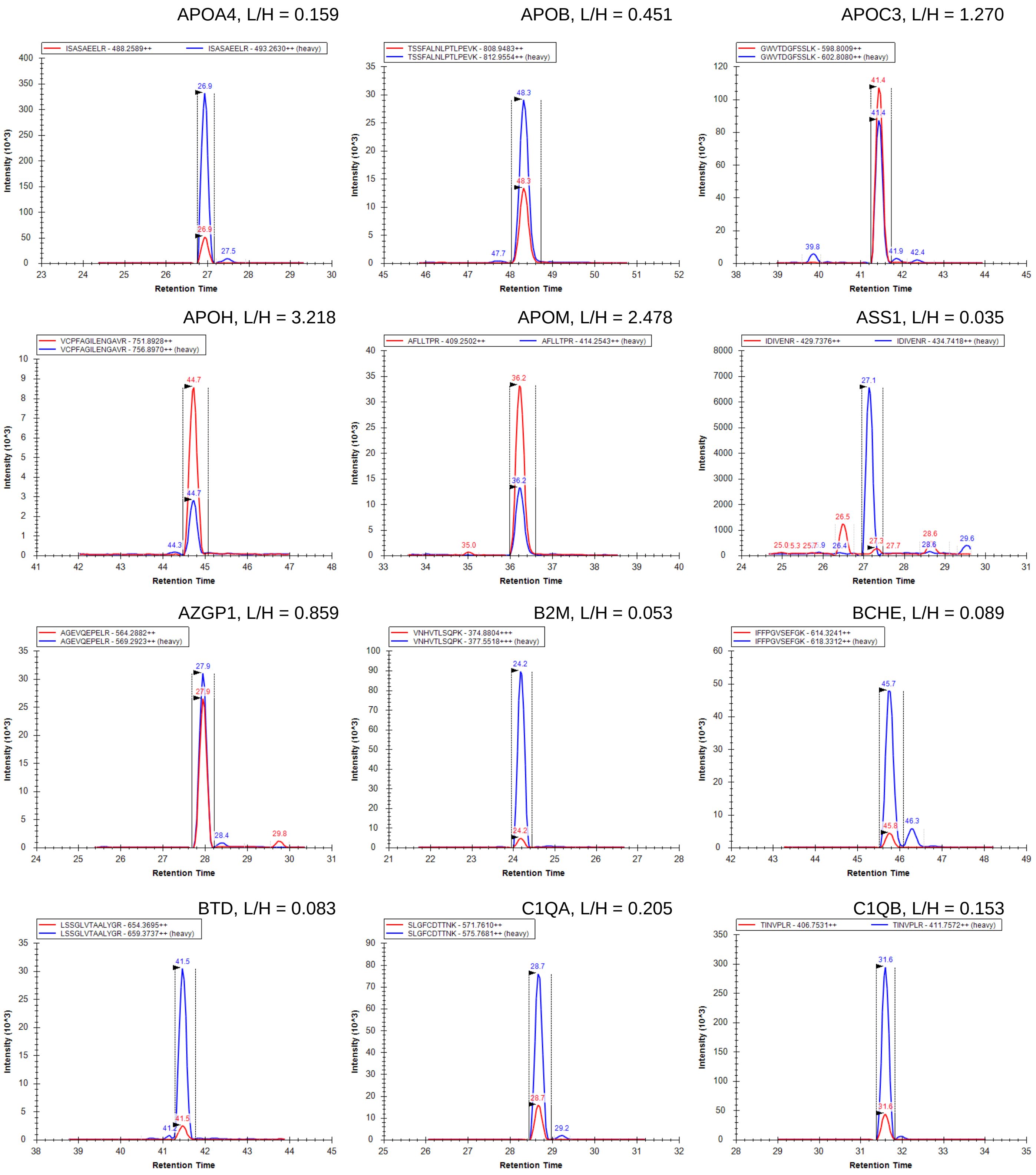

Supplementary Figure S1 (Continued)

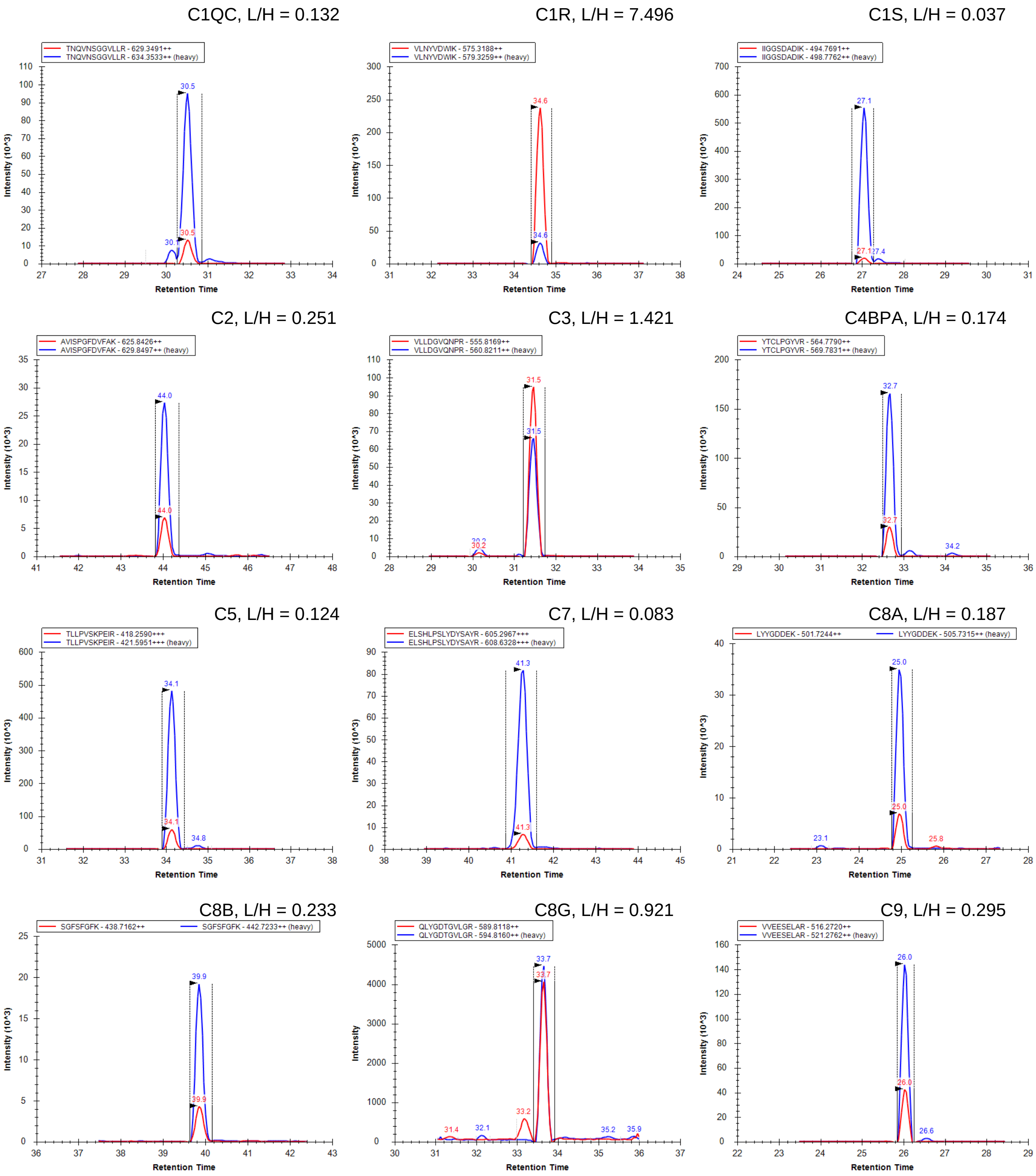

Supplementary Figure S1 (Continued)

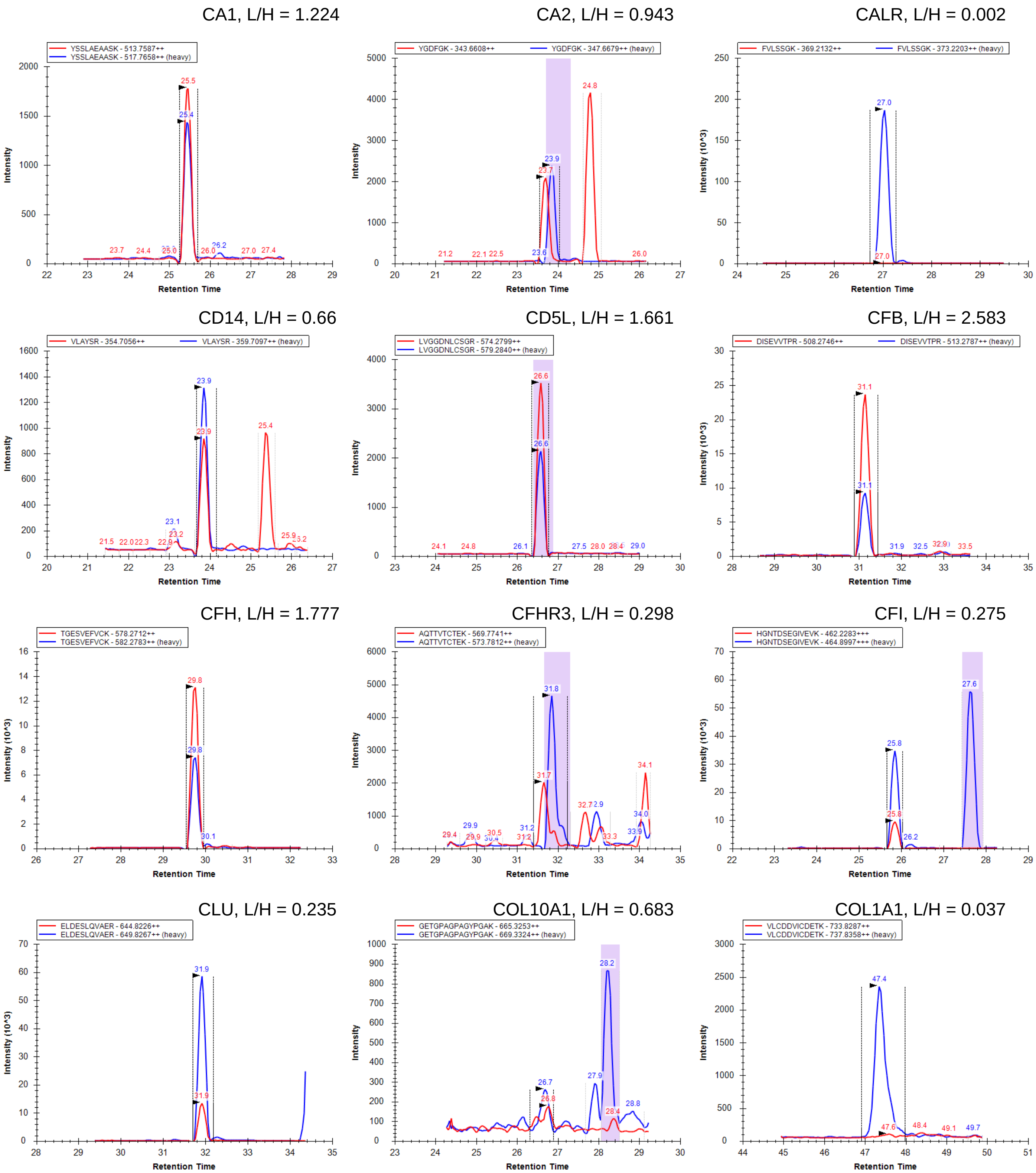

Supplementary Figure S1 (Continued)

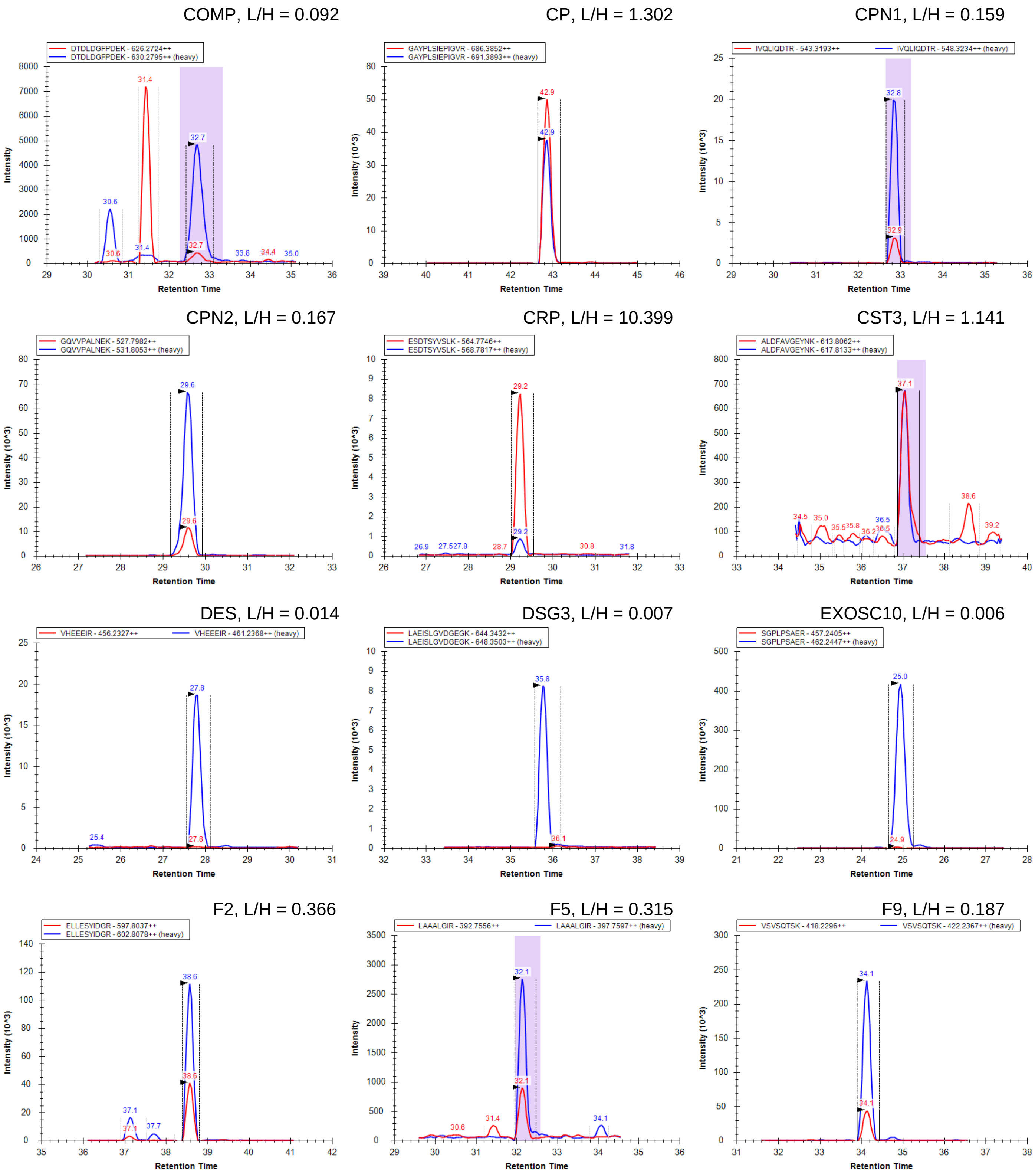

Supplementary Figure S1 (Continued)

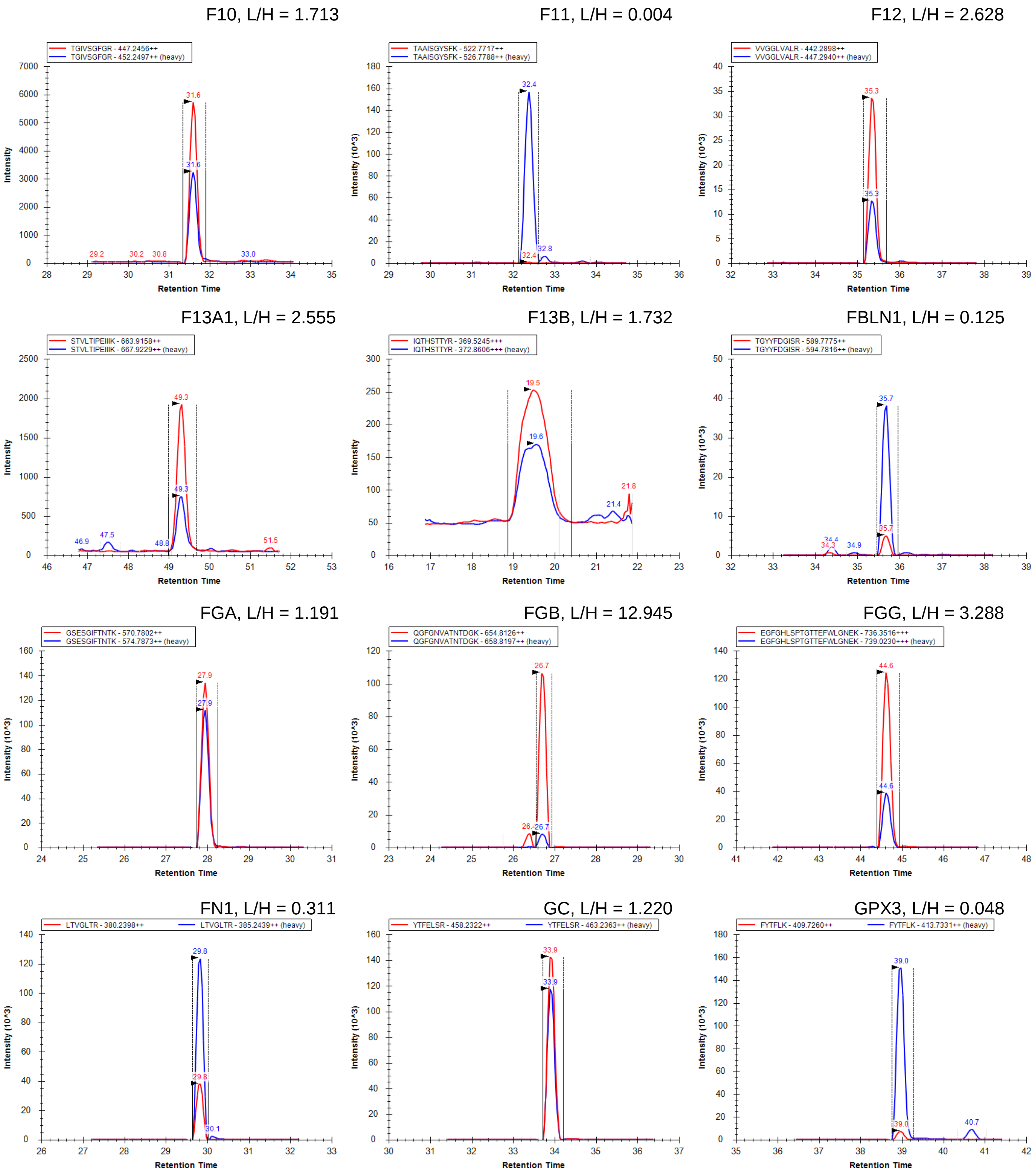

Supplementary Figure S1 (Continued)

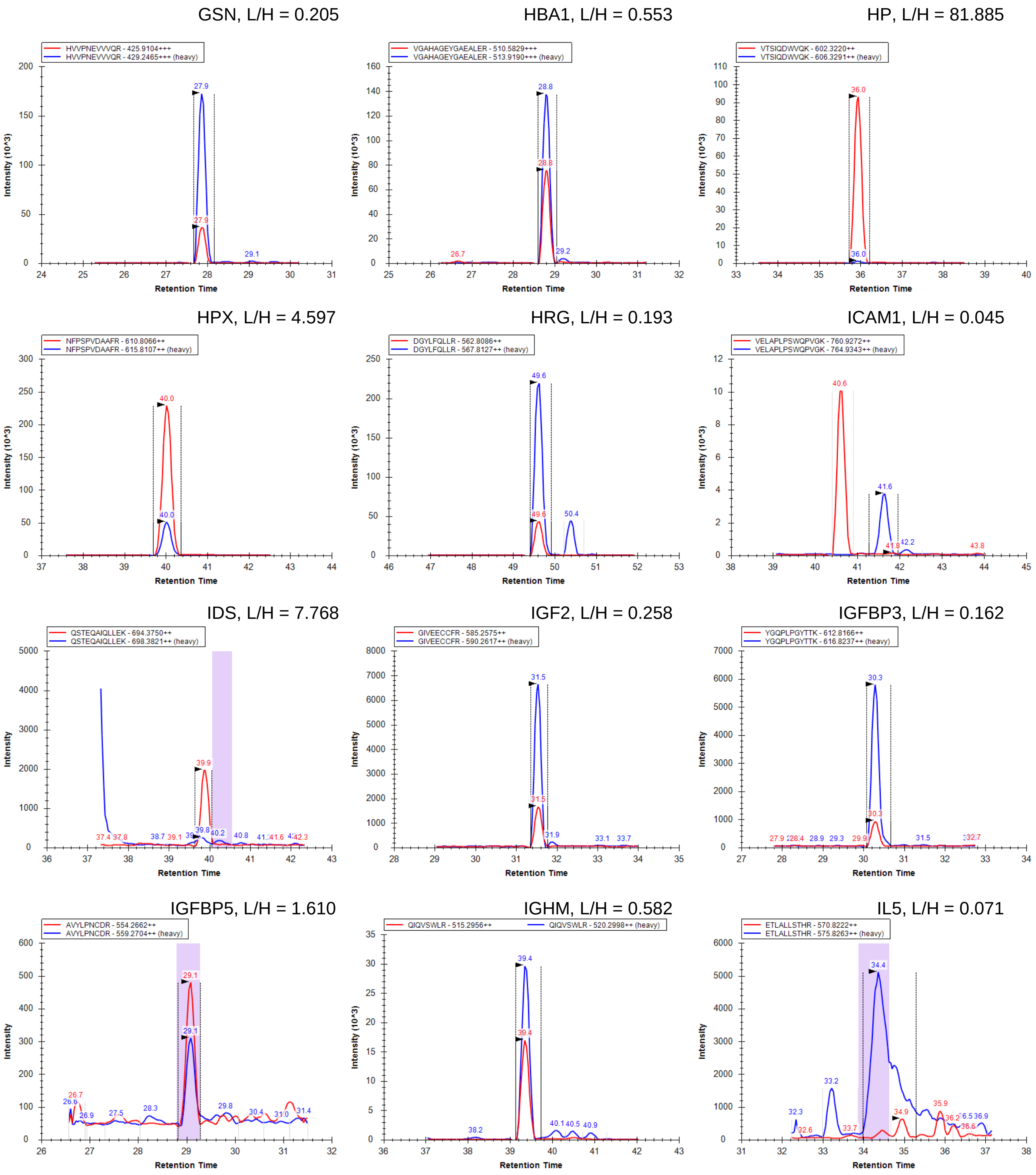

Supplementary Figure S1 (Continued)

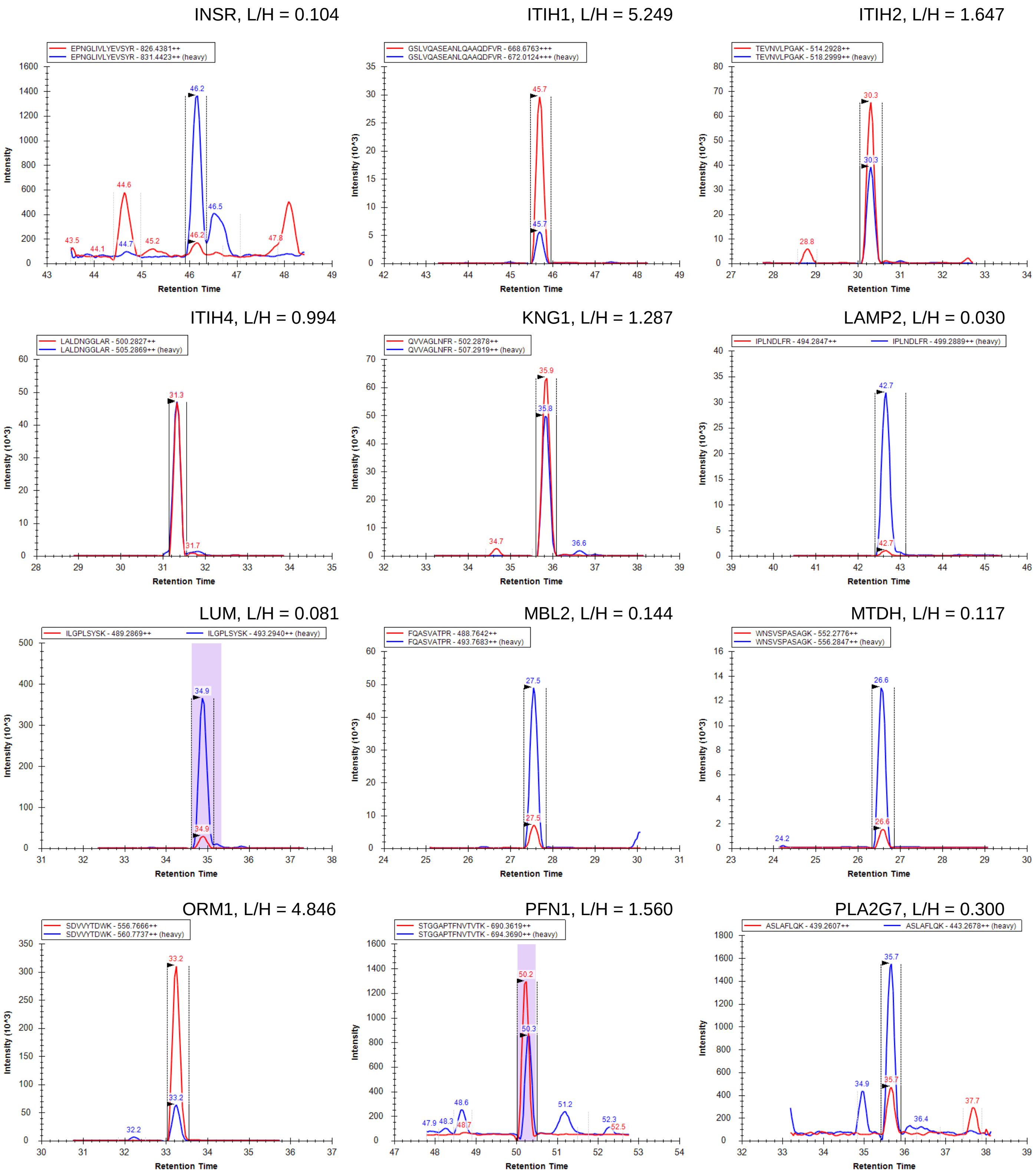

Supplementary Figure S1 (Continued)

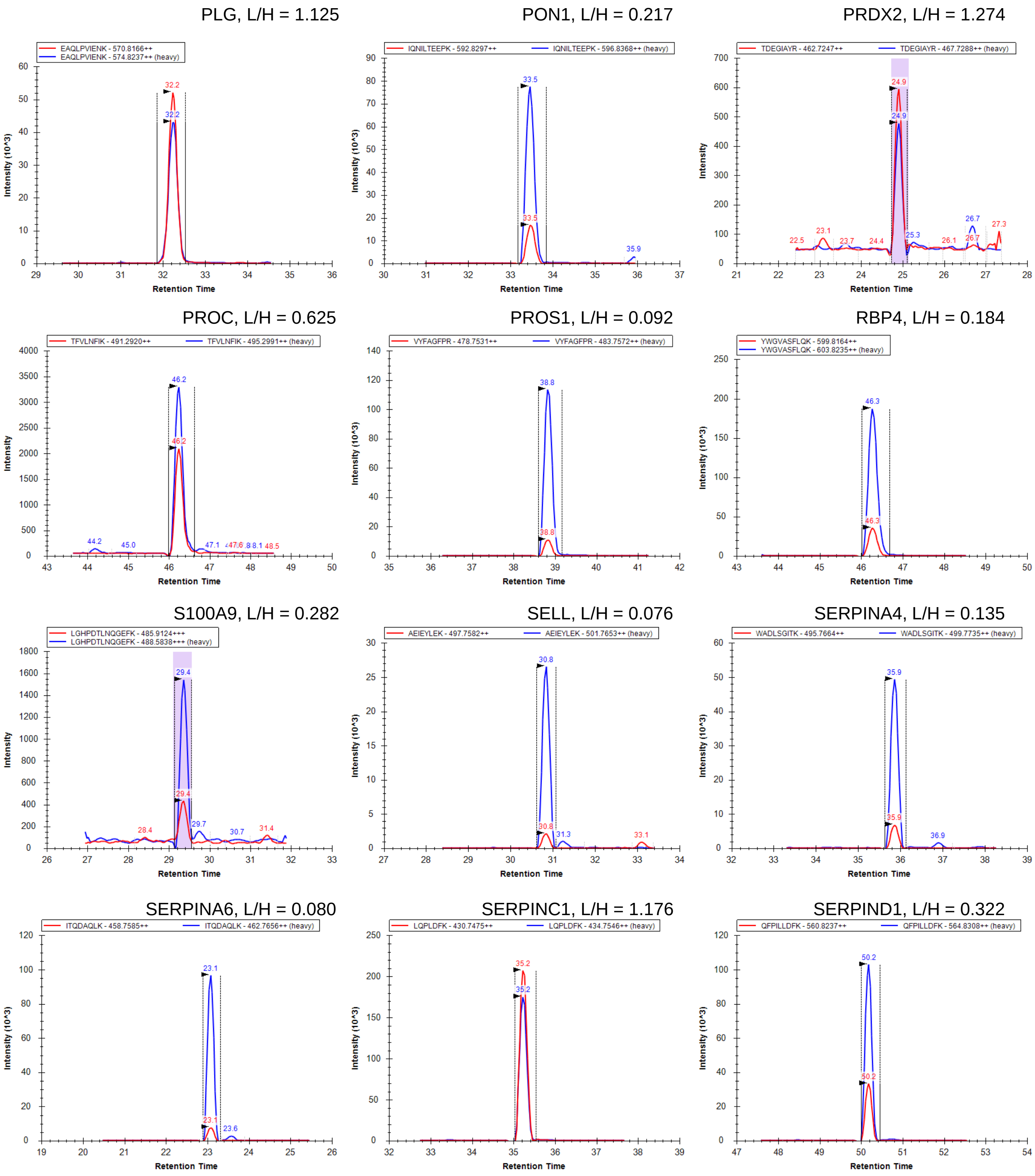

Supplementary Figure S1 (Continued)

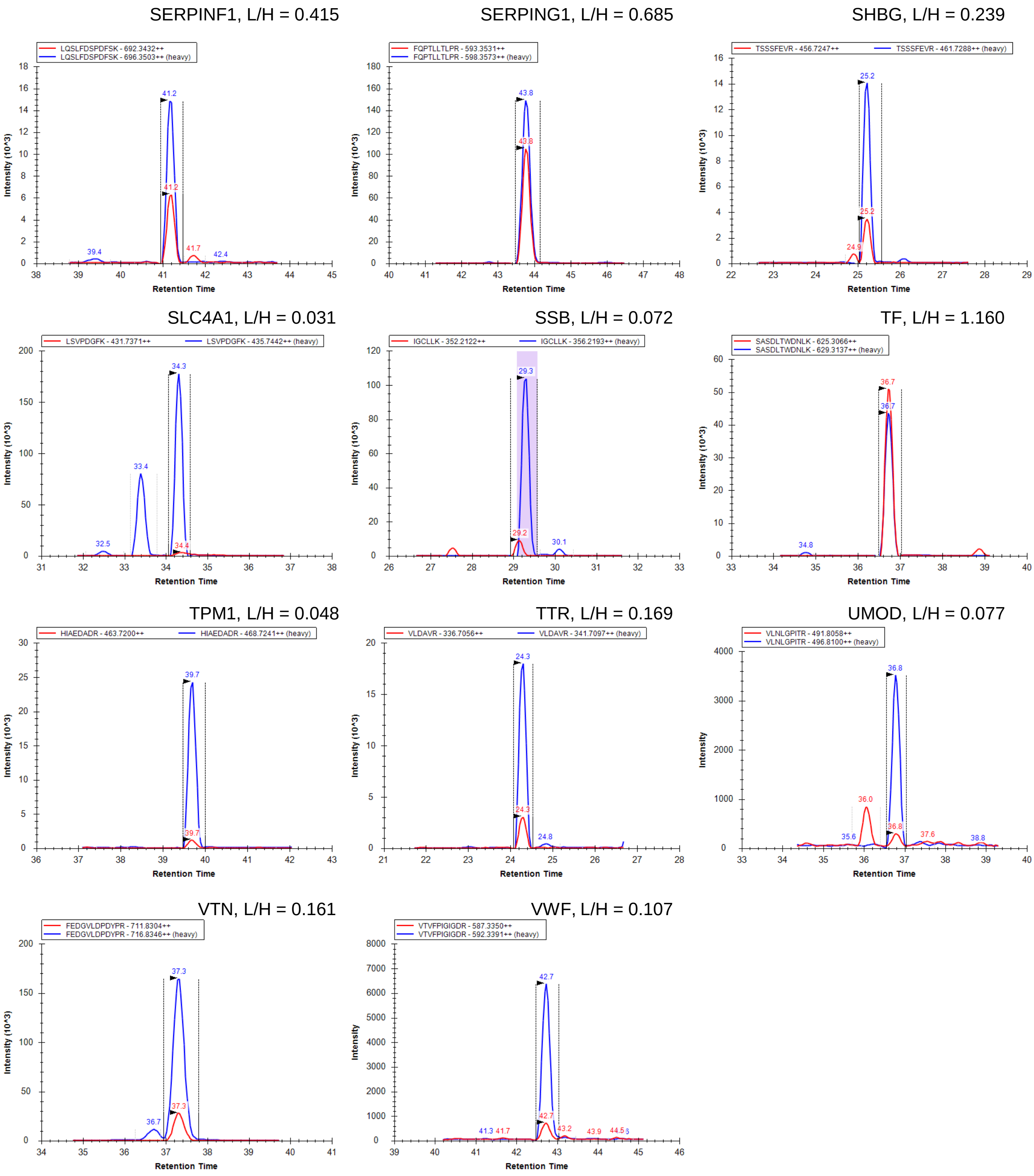

Supplementary Figure S1 (Continued)

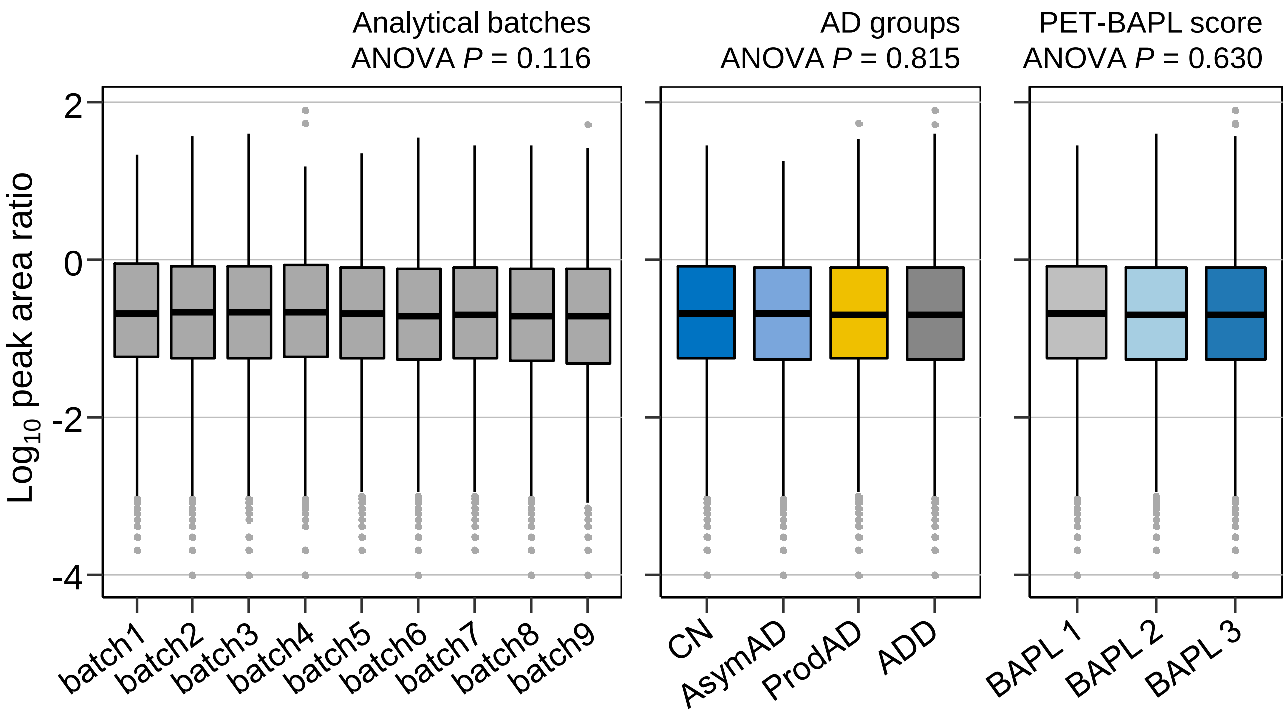

Supplementary Figure S2. Variation of overall 119 proteins levels in analytical batches, AD groups, and PET-BAPL score groups. Peak area ratios of 119 proteins from 185 subjects were measured by MRM-MS; their distribution between batches, cognitive control and AD patient groups, and BAPL scores are shown in box plots. Significance of differences in protein peak area ratios between groups was measured by one-way ANOVA.

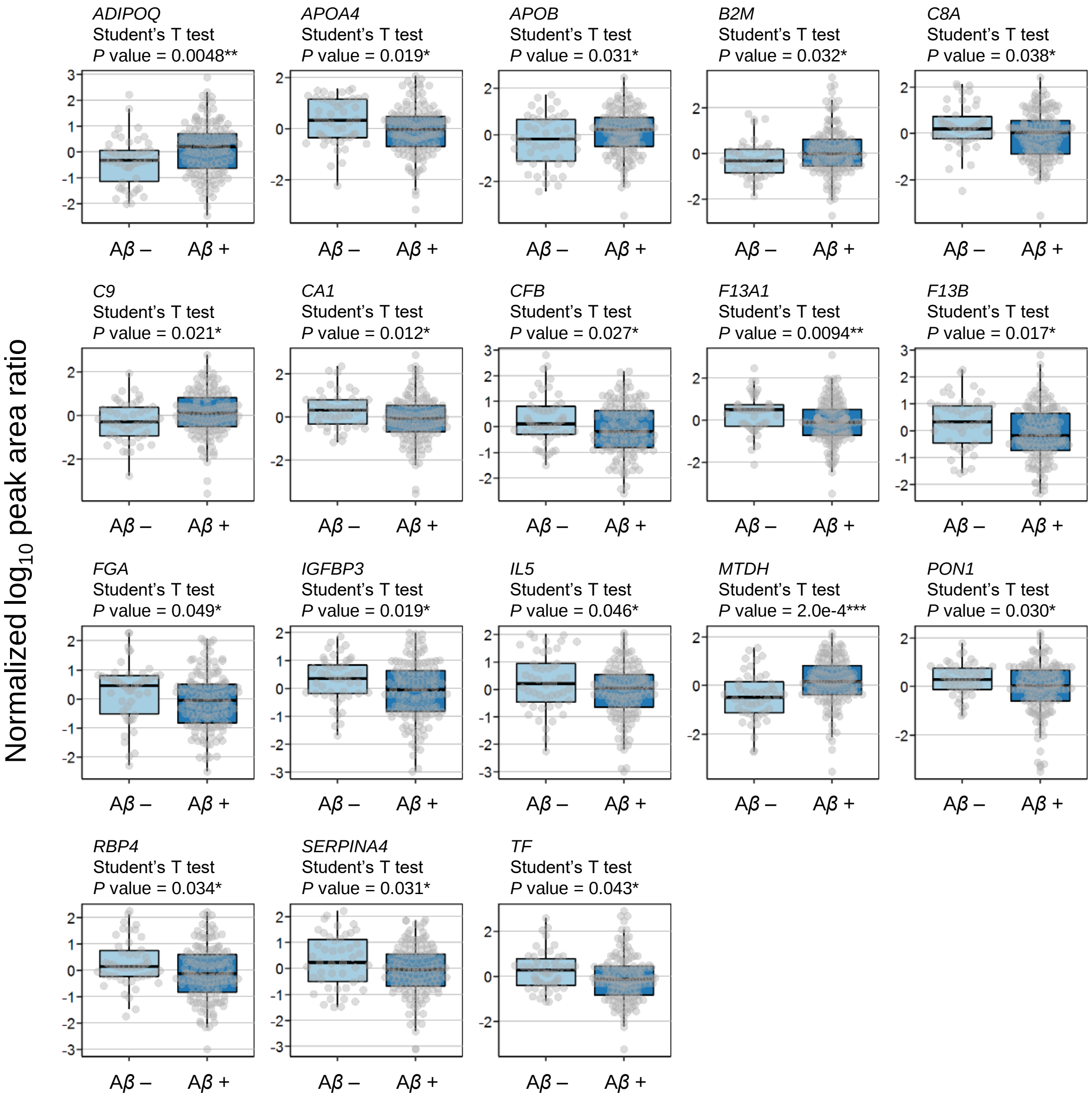

Supplementary Figure S3. The 18 differentially expressed proteins in PET-BAPL score groups. Box plots of 18 DEPs in the PET-BAPL groups: Amyloid-β-negative (PET-BAPL 1) and Amyloid-β-positive (PET-BAPL 2-3). Significant differences in protein expression of BAPL groups were measured by student's t-test, and significance levels are represented as "*" for $P < 0.05$; "**" for $P < 0.01$; and "***" for $P < 0.001$.

Prediction (APOE ε4)

References	AsymAD	ProdAD	ADD	*TPR*
AsymAD	0.0% (±0.0%)	17.2% (±3.0%)	10.8% (±3.0%)	0.0% (±0.0%)
ProdAD	0.0% (±0.0%)	18.0% (±3.4%)	18.0% (±3.4%)	50.0% (±9.5%)
ADD	0.0% (±0.0%)	22.3% (±0.9%)	13.7% (±0.6%)	38.0% (±2.0%)
FPR	NA	70.0% (±3.0%)	65.0% (±5.4%)	

Prediction (K-MMSE)

References	AsymAD	ProdAD	ADD	*TPR*
AsymAD	5.8% (±1.8%)	21.5% (±2.5%)	0.7% (±0.7%)	20.7% (±6.6%)
ProdAD	2.9% (±1.8%)	26.6% (±1.3%)	6.5% (±0.7%)	74.0% (±4.0%)
ADD	0.0% (±0.0%)	7.9% (±1.7%)	28.1% (±1.8%)	78.0% (±4.9%)
FPR	22.5% (±11.8%)	52.3% (±1.0%)	20.3% (±2.8%)	

Prediction (10 proteins, CV)

References	AsymAD	ProdAD	ADD	*TPR*
AsymAD	18.7% (±1.3%)	5.1% (±0.9%)	4.3% (±1.3%)	66.8% (±4.8%)
ProdAD	4.3% (±1.8%)	28.8% (±1.9%)	2.9% (±1.3%)	80.0% (±5.5%)
ADD	3.6% (±1.1%)	5.0% (±1.4%)	27.4% (±2.0%)	76.0% (±5.1%)
FPR	27.0% (±7.6%)	25.6% (±1.9%)	20.9% (±3.0%)	

Prediction (10 proteins with K-MMSE)

References	AsymAD	ProdAD	ADD	*TPR*
AsymAD	20.2% (±2.3%)	4.3% (±1.3%)	3.6% (±1.6%)	72.5% (±9.2%)
ProdAD	5.8% (±2.2%)	28.0% (±2.0%)	2.1% (±1.4%)	78.0% (±5.8%)
ADD	2.2% (±0.9%)	2.9% (±1.4%)	30.9% (±1.7%)	86.0% (±5.1%)
FPR	26.6% (±4.3%)	19.8% (±3.5%)	14.3% (±4.2%)	

Prediction (10 proteins, nCV)

References	AsymAD	ProdAD	ADD	*TPR*
AsymAD	19.4% (±0.8%)	6.5% (±1.8%)	2.1% (±1.4%)	69.3% (±2.8%)
ProdAD	3.6% (±2.0%)	26.7% (±3.3%)	5.7% (±1.8%)	74.0% (±8.7%)
ADD	4.3% (±1.4%)	5.1% (±1.8%)	26.6% (±2.1%)	74.0% (±6.0%)
FPR	28.5% (±3.9%)	29.0% (±5.5%)	19.8% (±6.3%)	

Supplementary Figure S4. Confusion matrix of prediction results of APOE genotyping, K-MMSE, the 10-protein models by CV and nested CV, and the model with 10 proteins and K-MMSE score. Prediction results of the 5 models for assessing neurocognitive impairments using data from the analysis of the 139 individual samples. Gray squares contain correct prediction results, and the proportion of classifications with 95% confidence intervals is shown. True positive rates (TPRs) and false positive rates (FPRs) for each group are presented in the matrices to show the diagnostic performance of the 5 models.

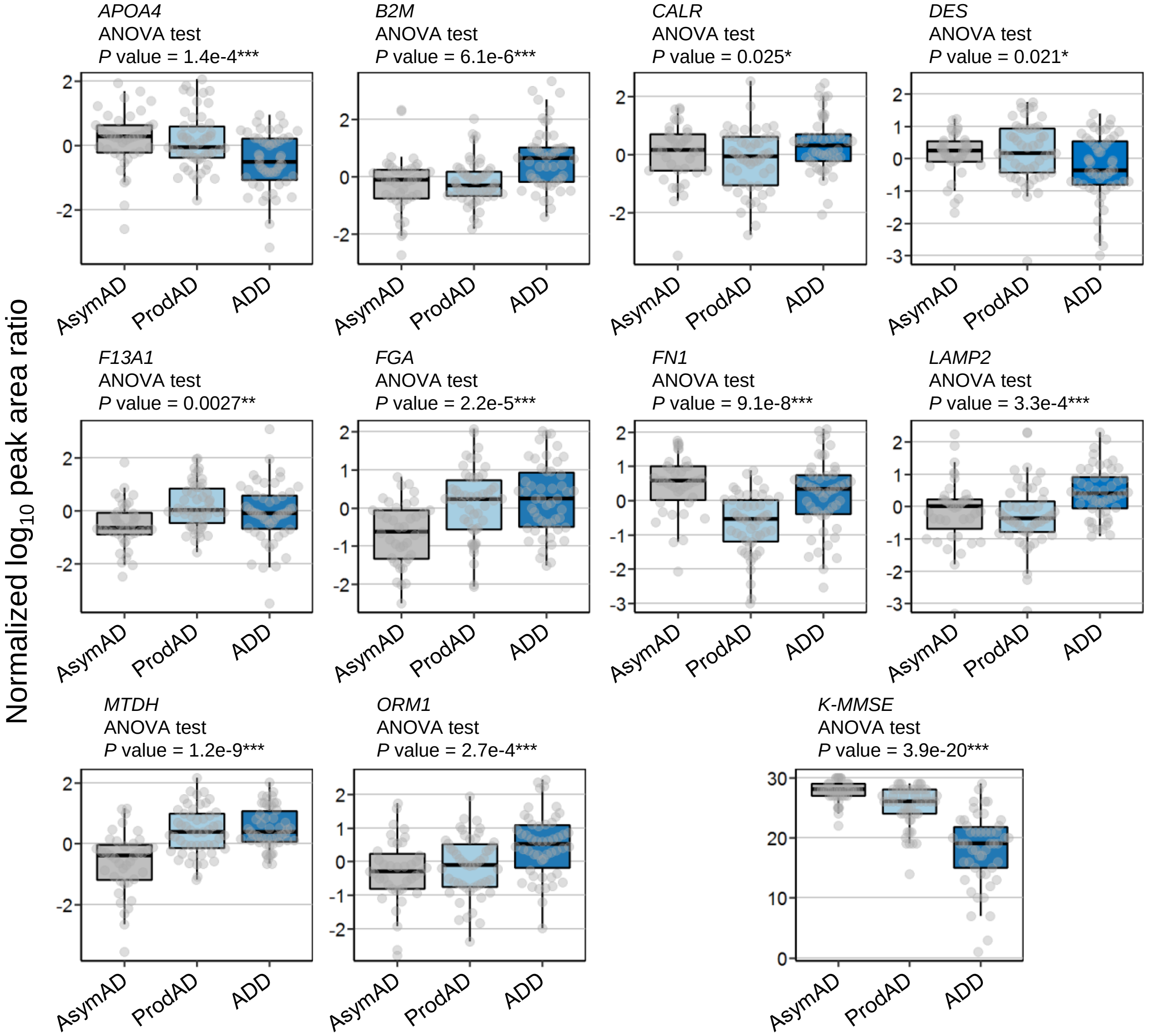

Supplementary Figure S5. Box plots of the 11 features in the final APM model. Relative plasma protein levels of the 10 proteins from asymptomatic AD (AsymAD) group, prodromal AD (ProdAD) group, and ADD group were measured by MRM-MS. K-MMSE scores of the subjects were measured and included in the final model. *P*-values for the comparison of protein levels and K-MMSE scores from the 3 groups were calculated by ANOVA. Significance levels are represented as "#" for $P < 0.1$; "**" for $P < 0.01$; and "***" for $P < 0.001$. Box plots represent the first, second, and third quartiles, and whiskers represent the 1.5 x interquartile range (IQR).

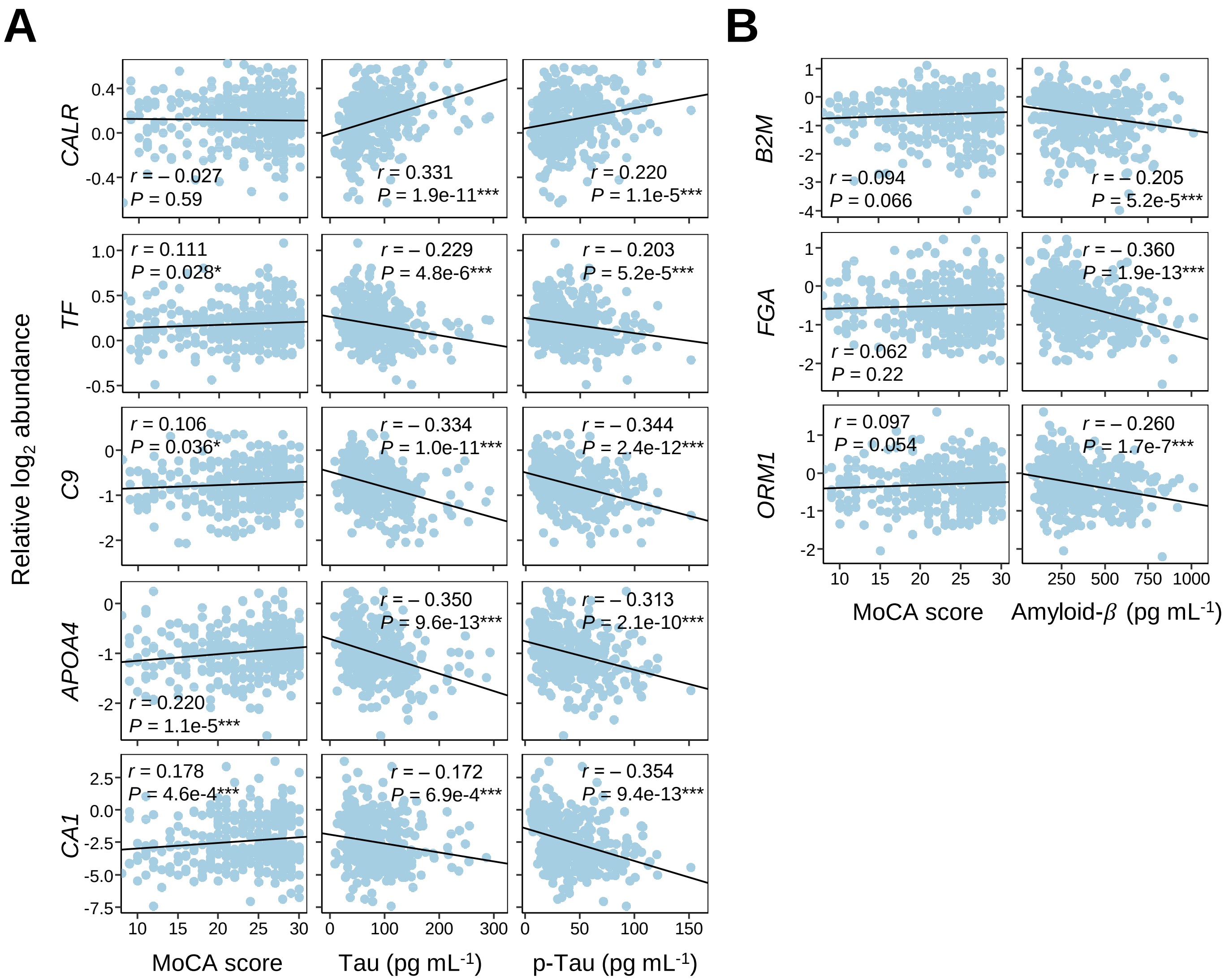

Supplementary Figure S6. CSF levels of proteins in the external CSF cohorts. Relative CSF levels of the proteins and their correlation with the original tests, such as cognitive score (MoCA), amyloid-β level, total tau level, and phospho-tau level in the CSF cohort [Cohort 1 (N = 297) and Cohort2 (N = 96) from Johnson et al.]. Pearson's correlation was used to measure the correlation between protein levels and original tests. Significance levels are indicated as "*" for $P < 0.05$; "**" for $P < 0.01$; and "***" for $P < 0.001$.
