# Supplementary material for: Plasma protein biomarker model for screening Alzheimer disease using multiple reaction monitoring-mass spectrometry
Source: Sci Rep. 2022 Jan 24;12:1282. doi: 10.1038/s41598-022-05384-8 (PMC8786819; doi:10.1038/s41598-022-05384-8)
Supplement: Supplementary file 2 — Supplementary Information 2. [file 41598_2022_5384_MOESM2_ESM.pdf]

Plasma Protein Biomarker Model for Screening Alzheimer Disease Using Multiple Reaction Monitoring-Mass Spectrometry

Yeongshin Kim[1,#]; Jaenyeon Kim[1,#]; Minsoo Son[1,#]; Jihyeon Lee[2]; Injoon Yeo[2], PhD; Kyu Yeong Choi[3], PhD; Hoowon Kim[3,4], MD, PhD; Byeong C. Kim[5], MD, PhD; Kun Ho Lee[3,6,7,*], PhD and Youngsoo Kim[1,2,*], PhD

[1]Interdisciplinary Program of Bioengineering, Seoul National University College of Engineering, Seoul, Republic of Korea; [2]Department of Biomedical Sciences, Seoul National University College of Medicine, Seoul, Republic of Korea; [3]Gwangju Alzheimer's & Related Dementia Cohort Research Center, Chosun University, Gwangju 61452, Republic of Korea; [4]Department of Neurology, Chosun University Hospital, Gwangju 61452, Republic of Korea; [5]Department of Neurology, Chonnam National University Medical School, Gwangju 61469, Republic of Korea; [6]Department of Biomedical Science, Chosun University, Gwangju 61452, Republic of Korea; [7]Aging Neuroscience Research Group, Korea Brain Research Institute, Daegu 41062, Republic of Korea

Supplementary methods

Depletion of high-abundance proteins

Six high-abundance proteins (albumin, immunoglobulin G, immunoglobulin A, haptoglobin, transferrin, and alpha-1-antitrypsin) were depleted on a high-performance liquid chromatography (HPLC) instrument that was coupled to a Multiple Affinity Removal System Human-6 (MARS Hu-6, 4.6 mm x 100 mm, Agilent, CA, USA). A total of 44 μL of each plasma sample was diluted with 176 μL MARS buffer A (Agilent, CA, USA) and filtered using 0.22 μm Spin-X filters (Corning Costar, NY, USA). Then, 200 μL of each diluted sample was injected into the HPLC instrument. The total run time for each run was 28 min. The MARS column was equilibrated with buffer A, and 200 μL of each diluted sample was loaded onto the MARS column at a flow rate of 0.5 mL/min for 10 min. Flow-through fractions, eluted at 3 min and containing low-abundance proteins, were used in subsequent experiments. The bound proteins were eluted at 16.5 min at a flow rate of 1.0 mL/min using 100% buffer B (Agilent, CA, USA).

Analytical target selection strategy

A FASTA file that contained the sequences of all 644 target proteins was inputted into Skyline[1] (Ver20.1, MacCoss Lab, University of Washington, USA), and *in silico* digestion, with the transition setting, was performed per the following criteria: trypsin proteolytic peptide, with no missed cleavage; peptide length, 6−30 amino acids; exclusion of peptides that contained methionine or RP/KP; structural modification, carbamidomethylation at cysteine; 2+, 3+ charge-state for precursor ions; 1+, 2+ charge-state for y, b fragment ions;

MS spectrum range, 10−1400 m/z; and Method match tolerance, 0.055 m/z. The transition patterns for each peptide were compared with the National Institute of Standards and Technology (NIST) reference library (http://peptide.nist.gov/), PeptideAtlas spectral library (http://www.peptideatlas.org/speclib/), and SWATHatlas spectral library (https://db.systemsbiology.net/sbeams/cgi/PeptideAtlas/GetDIALibs). A 431-protein set and a 732-peptide set were selected as detectable targets in plasma digests, with dot-product scores above 0.8, peak intensity greater than 800, and only unique tryptic peptides. Crude stable isotope-labeled standard (SIS) peptides (JPT Peptide Technology, Acton, MA, USA) were synthesized to quantify each protein.

Interference-free check in MRM-MS analyses

The automated detection of inaccurate and imprecise transitions (AuDIT) algorithm[2] was used to identify contributions from falsely transmitted ions. Transitions that met the criteria of consistent peak areas, repeated analysis [coefficient of variance (CV) < 30%], and an adjusted P value $>1\times10^{-5}$ for the comparison between the product ion intensity of endogenous peptides and the SIS peptides in the triplicate analyses were selected for further analysis. The pooled plasma digests of the training set samples were used as a matrix. All SIS peptides were spiked at 100-femtomole (fmol) quantities for the AuDIT analysis.

Protein quantitation and Quality control for individual sample analysis

Peak area ratios (endogenous-to-SIS peptide) were used to compare the relative abundance of target peptides between samples. The quantity of spiked SIS peptides were matched with each endogenous target level in the pooled matrix sample (Supplementary table

2). Log_{10}-transformed and protein-wise-centered, scaled peak area ratios (endogenous-to-SIS) were used for further data processing [the caret package (Version 6.0-86)]. In the individual sample analysis, single replicate analysis was performed. The proteins were used only when the skewness of the data was between -1.5 and 1.5, and a total of 119 proteins were verified in the individual sample analysis.

Nested Crossvalidation.

We have performed nested crossvalidation, using the Python scikit-learn package[3,4]. The dataset was randomly divided into 5 outer loops, and within each outer loop, datasets were again divided into 5 inner loops. Five sets of 4 training sets and 1 test set combination were used for the feature selection and model validation. Finally, we used a selection probability approach to select features. The most frequently selected features [appearing at least 4 times in the 5 inner loops (> 80%)] were validated using the test set from the outer loops. We reported the summarization of 5 estimates. To compare biasness, performance of same feature model were also validated by 5 fold crossvalidation of entire set, as a biased CV.

The AD model to classify amyloid beta positivity was initially developed using 18 differentially expressed proteins (MTDH, ADIPOQ, B2M, C9, APOB, FGA, TF, IL5, C8A, RBP4, CFB, SERPINA4, F13B, PON1, IGFBP3, APOA4, CA1, and F13A1). Features were selected from 18 proteins in each inner loop by recursive feature elimination (RFE).

Five sets of the final features were selected (Set 1: ADIPOQ, APOA4, APOB, C8A, C9, CA1, CFB, F13A1, F13B, FGA, MTDH, PON1, RBP4, and SERPINA4. Set 2: ADIPOQ, APOA4, APOB, B2M, C8A, C9, FGA, MTDH, SERPINA4, and TF. Set 3: ADIPOQ, APOA4, APOB, B2M, C8A, C9, CA1, CFB, F13A1, F13B, FGA, IGFBP3, IL5, MTDH,

PON1, RBP4, SERPINA4, and TF. Set 4: ADIPOQ, APOA4, APOB, CA1, F13A1, F13B, IGFBP3, IL5, MTDH, RBP4, and TF. Set 5: ADIPOQ, APOB, B2M, C9, CA1, F13A1, FGA, IL5, MTDH, RBP4, and TF). Each set had accuracy above 67% (78.4%, 81.1%, 67.6%, 67.6%, and 70.3%). Of 11 proteins that were selected in the original AD model from the first revision, 9 proteins (81.8%) were selected again from this trial and performed well, which indicates feature selection in the nested CV performed repeatedly well. Among the 5 feature sets, MTDH, ADIPOQ, APOB, TF, CA1, C9, APOA4, RBP4, F13A1, and FGA were most frequently selected, We selected 10 these proteins as the AD model features.

All 5 sets of outer loops were then used for model fitting to get coefficients. With AD model, the training set from each outer loop was used for model fitting, and the test set was used to validate the model. To compare nested cross validation with biased validation, we also performed 5 fold cross validation. The performance of our model, whether performed with nested crossvalidation(nCV AD model) or 5 fold crossvalidation(CV AD model), shown better accuracy then APOE ε4 alone (73.0%, 82.2%, and 67.4%, respectively). We then combined AD model with APOE e4 and validated the performance using 5 fold cross validation. The final model out performed both nCV model and CV model with higher AUROC(0.873, 0.817, and 0.847, respectively).

The same procedure was performed for the APM model (which categorized AsymAD, ProdAD, and ADD). A total of 32 differentially expressed proteins (HBA1, MTDH, PFN1, DSG3, CFHR3, F13A1, FGA, CST3, F13B, C7, AZGP1, APOB, FGB, FGG, FN1, TF, CFB, COMP, VTN, CRP, HP, LAMP2, B2M, ORM1, CALR, CFI, DES, UMOD, APOA4, RBP4, HRG, and SELL) were used for feature selection, and 5 sets of features were selected (Set 1: APOA4, B2M, CALR, CFB, CST3, F13A1, FGA, FN1, LAMP2, MTDH, ORM1, and PFN1; Set 2: APOA4, APOB, B2M, C7, CFHR3, CFI, DES, F13A1, FGA, FGG, FN1, LAMP2,

MTDH, PFN1, RBP4, SELL, and VTN; Set 3: APOA4, APOB, B2M, CALR, CFB, CFHR3, CFI, COMP, CRP, DES, F13A1, FGA, FGB, FGG, FN1, HRG, LAMP2, MTDH, ORM1, PFN1, and RBP4; Set 4: APOA4, APOB, AZGP1, B2M, C7, CALR, CFB, CFHR3, CFI, DES, F13A1, FGA, FN1, HRG, LAMP2, MTDH, ORM1, PFN1, RBP4, SELL, TF, and UMOD; Set 5: APOA4, B2M, CALR, DES, F13A1, FGA, FN1, LAMP2, MTDH, ORM1, and TF). Of 13 proteins from the original APM model, 8 proteins (61.5%) were selected again, by the selection probability approach. Among the 5 feature sets, APOA4, B2M, CALR, DES F13A1, FN1, FGA, LAMP2, MTDH, and ORM1 were chosen for features of APM model.

All 5 sets of outer loops were then used for model fitting to get coefficients. With AD model, the training set from each outer loop was used for model fitting, and the test set was used to validate the model. To compare nested cross validation with biased validation, we also performed 5 fold cross validation. The performance of our model, whether performed with nested crossvalidation(nCV APM model) or 5 fold crossvalidation(CV APM model), shown better accuracy then APOE ε4 or K-MMSE alone (74.8%, 72.7%, , 31.6%, and 60.4%, respectively). We then combined APM model with K-MMSE and validated the performance using 5 fold cross validation. The final model out performed both nCV model and CV model with higher Accuracy of 79.1%.

In summary, for the AD model, MTDH, ADIPOQ, B2M, C9, APOB, FGA, TF, IL5, C8A, CFB, APOA4, CA1, and F13A1 were selected. Trained and tested by each outer loop, the model performed with biased accuracy (83.3%, 83.3%, 80.6%, 66.7%, and 75.6%). For the APM model, APOB, PFN1, VTN, FGG, CRP, TF, CALR, F13A1, LAMP2, APOA4, ORM1, CFB, CST3, B2M, FN1, COMP, FGA, and MTDH were chosen as features. The model also performed biased results (accuracy: 88.9%, 82.1%, 60.7%, 78.6%, and 63.0%).

Method validation following CPTAC guidelines

To validate the assay, CPTAC guidelines was referenced to validate the limit of detection (LOD), limit of quantification (LOQ), and stability[5]. Pooled normal serum was used as a matrix, and of 119 target biomarkers, we chose 17 of most relevant targets for validation (APOA4, APOB, B2MG, CFAB, F13A, FIBA, LYRIC, CO9, CAH1, TRFE, ADIPO, CALR, COMP, CRP, CYTC, FINC, A1AG1).

As in the CPTAC guidelines, LOD is determined from a pure matrix that is injected before the first curve, using the average plus 3 times the standard deviation of the signal. To validate the LOQ, calibration curves were determined to validate the lower limit of quantification (LLOQ) of each target. Per CPTAC guidelines, the LLOQ should be determined as lowest point of the curve that provides enough linearity and a CV under 20%. In total, 14 points of the curve were prepared, from 1000 fmol to 0.122 fmol by 1:2 serial dilution. Each target was qualified with different LLOQ concentrations (Supplementary Table 10). For every target, points from the LLOQ to the highest point were used to calculate the slope and intercept of the curve. Concentrations (fmol) were than back-calculated by substituting the PAR value into the curve function (y=kx+b).

For stability, the experiment required 12 aliquots of the same sample, prepared with matrix that was spiked with 3 times the LLOQ concentration of the internal standard. Every aliquot was injected in duplicate, with the initial injection and follows by injection after at least 6 hours and then 24 hours. Freeze and thaw experiments were also required, with 3 aliquots being frozen and thawed once and injected and another 3 aliquots frozen and thawed 2 times and injected. Finally, after 4 weeks, 3 thawed aliquots were analyzed. With the data

from the experiments above, values for 6 hours, 24 hours, 1-time freeze/thaw, 2-time freeze/thaw, and 4 weeks were compared with the value for the initially injected aliquot, termed the 0 hour sample. The variability of the values needed to be within 20% of the 0 hour value. The results of the 17 target biomarkers are listed in Supplementary Tables 10-11. Most targets passed the evaluation, whereas COMP and FN1 failed once under the 24-hour condition, and ADIPOQ failed once under the 2-time freeze/thaw condition, with variability under 30%.

References

1. MacLean, B. *et al*. Skyline: an open source document editor for creating and analyzing targeted proteomics experiments. *Bioinformatics* **26**, 966-968 (2010)

2. Abbatiello, S.E., Mani, D. R., Keshishian, H., Carr, S. A., Automated detection of inaccurate and imprecise transitions in peptide quantification by multiple reaction monitoring mass spectrometry. *Clin Chem* **56**, 291-305 (2010)

3. Vabalas, A., Gowen, E., Poliakoff, E. & Casson, A. J. Machine learning algorithm validation with a limited sample size. *PLoS One* **14**, e0224365, doi:10.1371/journal.pone.0224365 (2019).

4. Parvandeh, S., Yeh, H. W., Paulus, M. P. & McKinney, B. A. Consensus features nested cross-validation. *Bioinformatics* **36**, 3093-3098, doi:10.1093/bioinformatics/btaa046 (2020).

5. Whiteaker, J. R. *et al*. CPTAC Assay Portal: a repository of targeted proteomic assays. *Nat Methods* **11**, 703-704, doi:10.1038/nmeth.3002 (2014).
