# Supplementary material for: Plasma protein biomarker model for screening Alzheimer disease using multiple reaction monitoring-mass spectrometry
Source: Sci Rep. 2022 Jan 24;12:1282. doi: 10.1038/s41598-022-05384-8 (PMC8786819; doi:10.1038/s41598-022-05384-8)
Supplement: Supplementary file 3 — Supplementary Information 3. [file 41598_2022_5384_MOESM3_ESM.pdf]

Supplementary Table 1. The text mining of references related to Alzheimer's disease.

References	DOI
Abu Bakar, Zulzikry Hafiz et al. "Effect of Age on the Protein Profile of Healthy Malay Adults and its Association with Cognitive Function Competency." Journal of Alzheimer's disease	10.3233/JAD-180511
Alemi, Mobina et al. "Transthyretin participates in beta-amyloid transport from the brain to the liver--involvement of the low-density lipoprotein receptor-related protein 1?." Scientific r	10.1038/srep20164
Alisi L, Cao R, De Angelis C, et al. The Relationships Between Vitamin K and Cognition: A Review of Current Evidence. Front Neurol. 2019;10:239. Published 2019 Mar 19.	10.3389/fneur.2019.00239
Babić Leko M, Nikolac Perković M, Klepac N, et al. Relationships of Cerebrospinal Fluid Alzheimer's Disease Biomarkers and COMT, DBH, and MAOB Single Nucleotide Polymorph	10.3233/JAD-190991
Baranger, Kevin et al. "Long-Term Pantethine Treatment Counteracts Pathologic Gene Dysregulation and Decreases Alzheimer's Disease Pathogenesis in a Transgenic Mouse Mod	10.1007/s13311-019-00754-z
Bartus, R T et al. "The cholinergic hypothesis of geriatric memory dysfunction." Science (New York, N.Y.) vol. 217,4558 (1982): 408-14.	10.1126/science.7046051
Benedet, Andréa L et al. "Epistasis analysis links immune cascades and cerebral amyloidosis." Journal of neuroinflammation vol. 12 227. 1 Dec. 2015.	10.1186/s12974-015-0436-z
Bis, Joshua C et al. "Whole exome sequencing study identifies novel rare and common Alzheimer's-Associated variants involved in immune response and transcriptional regulation."	10.1038/s41380-018-0112-7
Chauhan VP, Ray I, Chauhan A, Wisniewski HM. Binding of gelsolin, a secretory protein, to amyloid beta-protein. Biochem Biophys Res Commun. 1999;258(2):241-246.	10.1006/bbrc.1999.0623
Chen, Mei, and Weiming Xia. "Proteomic Profiling of Plasma and Brain Tissue from Alzheimer's Disease Patients Reveals Candidate Network of Plasma Biomarkers." Journal of Alzh	10.3233/JAD-200110
Chiba, T et al. "Amyloid-beta causes memory impairment by disturbing the JAK2/STAT3 axis in hippocampal neurons." Molecular psychiatry vol. 14,2 (2009): 206-22.	10.1038/mp.2008.105
Corsi MM, Licastro F, Porcellini E, et al. Reduced plasma levels of P-selectin and L-selectin in a pilot study from Alzheimer disease: relationship with neuro-degeneration. Biogeronto	10.1007/s10522-011-9335-6
Dahlbäck, Björn, and Bruno O Villoutreix. "Regulation of blood coagulation by the protein C anticoagulant pathway: novel insights into structure-function relationships and molecular r	10.1161/01.ATV.0000168421.13467.82
Gebre, Abadi Kahsu et al. "Targeting Renin-Angiotensin System Against Alzheimer's Disease." Frontiers in pharmacology vol. 9 440. 30 Apr. 2018.	10.3389/fphar.2018.00440
Heneka, M T et al. "Neuronal and glial coexpression of argininosuccinate synthetase and inducible nitric oxide synthase in Alzheimer disease." Journal of neuropathology and experir	10.1093/jnen/60.9.906
Ji, Lina et al. "Potential therapeutic implications of gelsolin in Alzheimer's disease." Journal of Alzheimer's disease : JAD vol. 44,1 (2015): 13-25.	10.3233/JAD-141548
Kang, Seokjo et al. "PiB-PET Imaging-Based Serum Proteome Profiles Predict Mild Cognitive Impairment and Alzheimer's Disease." Journal of Alzheimer's disease : JAD vol. 53,4 (2	10.3233/JAD-160025
Kim, Dong Kyu et al. "Deep proteome profiling of the hippocampus in the 5XFAD mouse model reveals biological process alterations and a novel biomarker of Alzheimer's disease." I	10.1038/s12276-019-0326-z
Kolev, Martin V et al. "Implication of complement system and its regulators in Alzheimer's disease." Current neuropharmacology vol. 7,1 (2009): 1-8.	10.2174/157015909787602805
Larvie, Mykol et al. "Mannose-binding lectin binds to amyloid β protein and modulates inflammation." Journal of biomedicine & biotechnology vol. 2012 (2012): 929803.	10.1155/2012/929803
Liu, Chia-Chen et al. "Apolipoprotein E and Alzheimer disease: risk, mechanisms and therapy." Nature reviews. Neurology vol. 9,2 (2013): 106-18.	10.1038/nrneurol.2012.263
Moon M, Song H, Hong HJ, et al. Vitamin D-binding protein interacts with Aβ and suppresses Aβ-mediated pathology. Cell Death Differ. 2013;20(4):630-638.	10.1038/cdd.2012.161
Muenchhoff, Julia et al. "Changes in the plasma proteome at asymptomatic and symptomatic stages of autosomal dominant Alzheimer's disease." Scientific reports vol. 6 29078. 6 Ju	10.1038/srep29078
Muhia, Mary et al. "The Kinesin KIF21B Regulates Microtubule Dynamics and Is Essential for Neuronal Morphology, Synapse Function, and Learning and Memory." Cell reports vol.	10.1016/j.celrep.2016.03.086
Naveed M, Mubeen S, Khan A, Ibrahim S, Meer B. Plasma Biomarkers: Potent Screeners of Alzheimer's Disease. Am J Alzheimers Dis Other Demen. 2019;34(5):290-301.	10.1177/1533317519848239
Olsson B, Lautner R, Andreasson U, et al. CSF and blood biomarkers for the diagnosis of Alzheimer's disease: a systematic review and meta-analysis. Lancet Neurol. 2016;15(7):67	10.1016/S1474-4422(16)00070-3
Paula-Lima AC, Tricerri MA, Brito-Moreira J, et al. Human apolipoprotein A-I binds amyloid-beta and prevents Abeta-induced neurotoxicity. Int J Biochem Cell Biol. 2009;41(6):1361-	10.1016/j.biocel.2008.12.003
Pollard, Amelia et al. "Mitochondrial proteomic profiling reveals increased carbonic anhydrase II in aging and neurodegeneration." Aging vol. 8,10 (2016): 2425-2436.	10.18632/aging.101064
Sanjurjo, Lucía et al. "CD5L Promotes M2 Macrophage Polarization through Autophagy-Mediated Upregulation of ID3." Frontiers in immunology vol. 9 480. 12 Mar. 2018.	10.3389/fimmu.2018.00480
Shen, Liming et al. "Proteomics Analysis of Blood Serums from Alzheimer's Disease Patients Using iTRAQ Labeling Technology." Journal of Alzheimer's disease : JAD vol. 56,1 (201	10.3233/JAD-160913
Shin TM, Isas JM, Hsieh CL, et al. Formation of soluble amyloid oligomers and amyloid fibrils by the multifunctional protein vitronectin. Mol Neurodegener. 2008;3:16. Published 2008	10.1186/1750-1326-3-16
Song F, Poljak A, Crawford J, et al. Plasma apolipoprotein levels are associated with cognitive status and decline in a community cohort of older individuals. PLoS One. 2012;7(6):e3	10.1371/journal.pone.0034078
Sundelöf, J et al. "Serum cystatin C and the risk of Alzheimer disease in elderly men." Neurology vol. 71,14 (2008): 1072-9.	10.1212/01.wnl.0000326894.40353.93
Trouw, Leendert A et al. "C4b-binding protein in Alzheimer's disease: binding to Abeta1-42 and to dead cells." Molecular immunology vol. 45,13 (2008): 3649-60.	10.1016/j.molimm.2008.04.025
Wang L, Chiang HC, Wu W, et al. Epidermal growth factor receptor is a preferred target for treating amyloid-β-induced memory loss. Proc Natl Acad Sci U S A. 2012;109(41):16743-	10.1073/pnas.1208011109
Webster, S et al. "Charge-based binding of complement component C1q to the Alzheimer amyloid beta-peptide." The American journal of pathology vol. 150,5 (1997): 1531-6.	NA (PubMed ID: 9137039)
Wolters, Frank J et al. "Von Willebrand factor and ADAMTS13 activity in relation to risk of dementia: a population-based study." Scientific reports vol. 8,1 5474. 3 Apr. 2018.	10.1038/s41598-018-23865-7
Yang, Hongqian et al. "Prognostic polypeptide blood plasma biomarkers of Alzheimer's disease progression." Journal of Alzheimer's disease : JAD vol. 40,3 (2014): 659-66.	10.3233/JAD-132102
Zamolodchikov, D et al. "The Alzheimer's disease peptide β-amyloid promotes thrombin generation through activation of coagulation factor XII." Journal of thrombosis and haemostas	10.1111/jth.13209
Zhang W, Huang W, Jing F. Contribution of blood platelets to vascular pathology in Alzheimer's disease. J Blood Med. 2013;4:141-147. Published 2013 Nov 8.	10.2147/JBM.S45071

Supplementary Table 2. Quality control information used to select quanfiable targets for 119 proteins.

Gene Name	Entry	Entry Name	Peptide Name	Precursor Charge	Product Charge	Fragment Ion	Light Precursor Mz	Light Product Mz	Heavy Precursor Mz	Heavy Product Mz	CE	RT	AuDIT, CV(%)	AuDIT, *P*	Skewness	Spiked SIS peptide level, fmol
A2M	P01023	A2MG_HUMAN	QGIPFFGQVR	2	1	y7	574.81	850.46	579.82	860.47	20.3	43.7	3.1	0.9932	0.152	1000.0
ADAMTS13	Q76LX8	ATS13_HUMAN	LFINVAPHAR	3	2	y8	379.89	439.25	383.23	444.26	6.5	34.3	12.2	0.9556	-0.089	10.0
ADIPOQ	Q15848	ADIPO_HUMAN	GDIGETGVPGAEGPR	2	1	y7	706.34	683.35	711.35	693.36	25.0	29.7	24.2	1.0000	-0.013	31.7
AFM	P43652	AFAM_HUMAN	FTFEYSR	2	1	y5	475.22	701.33	480.23	711.33	16.7	34.0	0.5	0.9891	-0.572	86.6
AGT	P01019	ANGT_HUMAN	DPTFIPAPIQAK	2	1	y7	649.36	724.44	653.37	732.45	23.0	39.4	14.2	0.9907	0.425	1000.0
AHSG	P02765	FETUA_HUMAN	EHAVEGDCDFQLLK	3	1	y5	554.26	648.41	556.93	656.42	13.4	35.9	12.1	1.0000	-1.234	1000.0
ALB	P02768	ALBU_HUMAN	LVNEVTEFAK	2	1	y8	575.31	937.46	579.32	945.48	20.3	34.7	4.6	0.9784	-0.868	233.9
ALDOA	P04075	ALDOA_HUMAN	ALQASALK	2	1	b2	401.25	185.13	405.25	185.13	14.1	25.5	19.3	0.3172	-0.574	531.3
AMBP	P02760	AMBP_HUMAN	TVAACNLPIVR	2	1	y4	607.34	484.32	612.34	494.33	21.5	35.1	15.3	0.7612	-1.172	1000.0
APCS	P02743	SAMP_HUMAN	VFVFPR	2	1	y4	382.73	518.31	387.73	528.32	13.4	36.9	10.2	0.7141	-0.286	64.3
APOA1	P02647	APOA1_HUMAN	DLATVYVDVLK	2	1	y6	618.35	736.42	622.35	744.44	21.8	45.9	22.1	0.8190	-0.063	1000.0
APOA2	P02652	APOA2_HUMAN	SPELQAEAK	2	2	y8	486.75	443.24	490.76	447.24	17.1	23.5	2.8	0.6299	-0.004	1000.0
APOA4	P06727	APOA4_HUMAN	ISASAEELR	2	1	y7	488.26	775.39	493.26	785.40	17.2	26.9	2.2	0.7057	-0.598	1000.0
APOB	P04114	APOB_HUMAN	TSSFALNLPTLPEVK	2	1	y7	808.95	783.46	812.96	791.48	28.7	48.4	3.3	0.8856	-0.395	434.0
APOC3	P02656	APOC3_HUMAN	GWVTDGFSSLK	2	1	y8	598.80	854.43	602.81	862.44	21.1	41.5	6.9	0.9323	0.102	701.2
APOH	P02749	APOH_HUMAN	VCPFAGILENGAVR	2	1	y7	751.89	758.42	756.90	768.42	26.6	44.5	25.0	0.9791	-1.194	352.9
APOM	O95445	APOM_HUMAN	AFLLTPR	2	1	y4	409.25	486.30	414.25	496.31	14.4	36.1	5.9	0.9892	-0.542	56.3
ASS1	P00966	ASSY_HUMAN	IDIVENR	2	1	y5	429.74	630.36	434.74	640.37	15.1	27.3	26.2	0.1470	-0.428	32.3
AZGP1	P25311	ZA2G_HUMAN	AGEVQEPELR	2	1	y6	564.29	771.40	569.29	781.41	19.9	27.9	8.5	0.9523	-0.547	576.1
B2M	P61769	B2MG_HUMAN	VNHVTLSQPK	3	1	y4	374.88	459.26	377.55	467.27	6.3	24.2	13.1	0.4148	0.531	1000.0
BCHE	P06276	CHLE_HUMAN	IFFPGVSEFGK	2	1	y8	614.32	820.42	618.33	828.43	21.7	45.8	11.7	0.9788	0.111	110.6
BTD	P43251	BTD_HUMAN	LSSGLVTAALYGR	2	1	y7	654.37	751.41	659.37	761.42	23.1	41.3	23.6	0.9975	-0.488	168.4
C1QA	P02745	C1QA_HUMAN	SLGFCDTTNK	2	1	y8	571.76	942.40	575.77	950.41	20.2	28.7	17.4	1.0000	-1.493	1000.0
C1QB	P02746	C1QB_HUMAN	TINVPLR	2	1	y5	406.75	598.37	411.76	608.38	14.3	31.6	2.7	0.1593	0.225	543.7
C1QC	P02747	C1QC_HUMAN	TNQVNSGGVLLR	2	1	y8	629.35	815.47	634.35	825.48	22.2	30.5	6.7	0.9856	-0.086	1000.0
C1R	P00736	C1R_HUMAN	VLNYVDWIK	2	1	y7	575.32	937.48	579.33	945.49	20.3	34.7	5.5	0.9994	-0.894	98.0
C1S	P09871	C1S_HUMAN	IIGGSDADIK	2	1	y8	494.77	762.36	498.78	770.38	17.4	27.1	7.0	0.3439	-0.644	1000.0
C2	P06681	CO2_HUMAN	AVISPGFDVFAK	2	2	y8	625.84	440.73	629.85	444.74	22.1	44.1	8.4	0.9954	-0.228	155.9
C3	P01024	CO3_HUMAN	VLLDGVQNPR	2	1	y7	555.82	785.39	560.82	795.40	19.6	31.5	2.4	0.9578	-0.054	844.7
C4BPA	P04003	C4BPA_HUMAN	YTCLPGYVR	2	1	y5	564.78	591.32	569.78	601.33	19.9	32.7	16.9	0.9913	-1.065	249.1
C5	P01031	CO5_HUMAN	TLLPVSKPEIR	3	2	y8	418.26	463.28	421.60	468.28	8.0	34.2	5.1	0.9997	-0.089	1000.0
C7	P10643	CO7_HUMAN	ELSHLPSLYDYSAYR	3	1	y6	605.30	774.34	608.63	784.35	15.4	41.4	27.4	1.0000	-0.547	560.3
C8A	P07357	CO8A_HUMAN	LYYGDDEK	2	1	y6	501.72	726.29	505.73	734.31	17.7	25.0	7.9	0.9259	-0.164	268.8
C8B	P07358	CO8B_HUMAN	SGFSFGFK	2	1	y5	438.72	585.30	442.72	593.32	15.4	39.9	2.5	0.1276	-0.205	99.6
C8G	P07360	CO8G_HUMAN	QLYGDTGVLGR	2	1	y8	589.81	774.41	594.82	784.42	20.8	33.6	11.5	0.9872	-0.138	125.9
C9	P02748	CO9_HUMAN	VVEESELAR	2	1	y7	516.27	833.40	521.28	843.41	18.2	26.1	7.7	0.7973	-0.317	516.3
CA1	P00915	CAH1_HUMAN	YSSLAEAASK	2	1	y8	513.76	776.41	517.77	784.43	18.1	25.5	10.2	0.9973	-0.319	10.0
CA2	P00918	CAH2_HUMAN	YGDFGK	2	1	y5	343.66	523.25	347.67	531.27	12.0	23.7	19.9	0.8561	-0.055	52.6
CALR	P27797	CALR_HUMAN	FVLSSGK	2	1	y5	369.21	491.28	373.22	499.30	12.9	27.0	12.0	0.3735	-0.337	485.6
CD14	P08571	CD14_HUMAN	VLAYSR	2	1	y4	354.71	496.25	359.71	506.26	12.4	23.9	24.0	1.0000	-0.321	11.2
CD5L	O43866	CD5L_HUMAN	LVGGDNLCSGR	2	1	y9	574.28	935.40	579.28	945.41	20.3	26.6	2.1	0.9234	-0.846	10.0
CFB	P00751	CFAB_HUMAN	DISEVVTPR	2	1	y4	508.27	472.29	513.28	482.30	17.9	31.2	1.0	0.9037	0.131	137.5
CFH	P08603	CFAH_HUMAN	TGESVEFVCK	2	1	y5	578.27	682.32	582.28	690.34	20.4	29.8	6.3	0.9351	-1.382	67.2
CFHR3	Q02985	FHR3_HUMAN	AQTTVTCTEK	2	1	b2	569.77	200.10	573.78	200.10	20.1	31.7	26.7	0.9098	-0.765	10.0
CFI	P05156	CFAI_HUMAN	HGNTDSEGIVEVK	3	1	y4	462.23	474.29	464.90	482.31	9.7	25.9	25.9	0.9997	0.210	238.8
CLU	P10909	CLUS_HUMAN	ELDESLQVAER	2	1	y7	644.82	802.44	649.83	812.45	22.8	31.9	4.5	0.8855	-0.261	1000.0
COL10A1	Q03692	COAA1_HUMAN	GETGPAGPAGYPGAK	2	1	y6	665.33	592.31	669.33	600.32	23.5	26.8	5.8	0.4459	-1.195	21.1
COL1A1	P02452	CO1A1_HUMAN	VLCDDVICDETK	2	1	y5	733.83	652.26	737.84	660.27	26.0	47.6	23.0	1.0000	-0.208	10.0
COMP	P49747	COMP_HUMAN	DTDLDGFPDEK	2	1	y7	626.27	807.35	630.28	815.37	22.1	32.7	27.1	1.0000	-0.934	62.4
CP	P00450	CERU_HUMAN	GAYPLSIEPIGVR	2	1	y8	686.39	870.50	691.39	880.51	24.3	42.6	14.8	0.8459	-0.382	594.1
CPN1	P15169	CBPN_HUMAN	IVQLIQDTR	2	1	y6	543.32	745.42	548.32	755.43	19.2	32.9	14.3	0.9750	-0.052	362.4
CPN2	P22792	CPN2_HUMAN	GQVVPALNEK	2	1	y6	527.80	671.37	531.81	679.39	18.6	29.7	15.8	0.7434	-0.143	221.0
CRP	P02741	CRP_HUMAN	ESDTSYVSLK	2	1	y4	564.77	446.30	568.78	454.31	19.9	29.4	22.7	0.8582	0.712	18.6
CST3	P01034	CYTC_HUMAN	ALDFAVGEYNK	2	1	y7	613.81	780.39	617.81	788.40	21.7	37.0	16.8	0.4500	-0.040	10.3
DES	P17661	DESM_HUMAN	VHEEEIR	2	1	y2	456.23	288.20	461.24	298.21	16.0	27.8	26.1	0.5885	-1.410	10.0
DSG3	P32926	DSG3_HUMAN	LAEISLGVDGEGK	2	1	y9	644.34	861.43	648.35	869.45	22.8	35.9	27.5	0.3486	-0.839	45.6
EXOSC10	Q01780	EXOSX_HUMAN	SGPLPSAER	2	1	y5	457.24	559.28	462.24	569.29	16.1	24.9	9.1	0.1484	-1.436	914.5

F10	P00742	FA10_HUMAN	TGIVSGFGR	2	1	y6	447.25	622.33	452.25	632.34	15.7	31.7	5.5	0.9339	-0.369	18.0
F11	P03951	FA11_HUMAN	TAAISGYSFK	2	1	y6	522.77	688.33	526.78	696.34	18.4	32.3	13.5	1.0000	-0.517	853.1
F12	P00748	FA12_HUMAN	VVGGLVALR	2	1	y7	442.29	685.44	447.29	695.44	15.5	35.4	6.5	0.9193	0.096	14.4
F13A1	P00488	F13A_HUMAN	STVLTIPEIIIK	2	1	y6	663.92	712.46	667.92	720.47	23.5	49.3	26.3	0.6103	-0.100	20.0
F13B	P05160	F13B_HUMAN	IQTHSTTYR	3	2	y7	369.52	433.21	372.86	438.22	6.0	19.7	29.1	0.8659	0.036	16.3
F2	P00734	THRB_HUMAN	ELLESYIDGR	2	1	y6	597.80	710.35	602.81	720.36	21.1	38.6	4.1	0.8795	-0.176	458.3
F5	P12259	FA5_HUMAN	LAAALGIR	2	1	y7	392.76	671.42	397.76	681.43	13.8	32.1	20.5	0.7659	-0.391	26.0
F9	P00740	FA9_HUMAN	VSVSQTSK	2	2	b4	418.23	187.11	422.24	187.11	14.7	34.2	15.8	0.9987	0.165	145.5
FBLN1	P23142	FBLN1_HUMAN	TGYYFDGISR	2	1	y6	589.78	694.35	594.78	704.36	20.8	35.7	16.0	0.9953	-0.143	193.6
FGA	P02671	FIBA_HUMAN	GSESGIFTNTK	2	1	y5	570.78	610.32	574.79	618.33	20.1	28.0	7.8	1.0000	-0.058	1000.0
FGB	P02675	FIBB_HUMAN	QGFGNVATNTDGK	2	1	y7	654.81	706.34	658.82	714.35	23.2	26.8	7.4	0.9843	-0.317	1000.0
FGG	P02679	FIBG_HUMAN	EGFGHLSPTGTTEFWLGNEK	3	1	y4	736.35	447.22	739.02	455.23	20.6	44.4	8.7	0.9013	-0.005	1000.0
FN1	P02751	FINC_HUMAN	LTVGLTR	2	1	y5	380.24	545.34	385.24	555.35	13.3	29.8	10.3	0.9511	-0.256	663.2
GC	P02774	VTDB_HUMAN	YTFELSR	2	1	y5	458.23	651.35	463.24	661.35	16.1	34.0	3.5	0.7795	-0.986	951.3
GPX3	P22352	GPX3_HUMAN	FYTFLK	2	1	y4	409.73	508.31	413.73	516.33	14.4	38.9	12.0	0.9592	-0.239	580.1
GSN	P06396	GELS_HUMAN	HVVPNEVVVQR	3	1	y4	425.91	501.31	429.25	511.32	8.3	27.8	5.1	1.0000	-0.396	1000.0
HBA1	P69905	HBA_HUMAN	VGAHAGEYGAEALER	3	1	y4	510.58	488.28	513.92	498.29	11.6	28.8	9.9	0.7033	0.677	1000.0
HP	P00738	HPT_HUMAN	VTSIQDWVQK	2	1	y6	602.32	803.40	606.33	811.42	21.3	36.1	6.3	0.9943	0.013	33.5
HPX	P02790	HEMO_HUMAN	NFPSPVDAAFR	2	2	y7	610.81	388.21	615.81	393.21	21.6	40.1	5.6	0.6315	-0.035	1000.0
HRG	P04196	HRG_HUMAN	DGYLFQLLR	2	1	y5	562.81	676.41	567.81	686.42	19.9	49.5	2.0	0.7609	-0.426	1000.0
ICAM1	P05362	ICAM1_HUMAN	VELAPLPSWQPVGK	2	1	b2	760.93	229.12	764.93	229.12	27.0	41.6	15.2	0.9952	-0.319	10.0
IDS	P22304	IDS_HUMAN	QSTEQAIQLLEK	2	1	y5	694.38	630.38	698.38	638.40	24.6	39.9	21.9	1.0000	0.730	200.8
IGF2	P01344	IGF2_HUMAN	GIVEECCFR	2	1	y6	585.26	900.33	590.26	910.34	20.7	31.6	9.3	0.7212	-0.789	156.2
IGFBP3	P17936	IBP3_HUMAN	YGQPLPGYTTK	2	1	y6	612.82	666.35	616.82	674.36	21.7	30.3	22.5	0.9995	-0.468	55.8
IGFBP5	P24593	IBP5_HUMAN	AVYLPNCDR	2	1	y5	554.27	661.27	559.27	671.28	19.6	29.1	16.6	1.0000	-1.206	4.0
IGHM	P01871	IGHM_HUMAN	QIQVSWLR	2	1	y4	515.30	561.31	520.30	571.32	18.2	39.5	15.6	0.9897	-0.680	630.5
IL5	P05113	IL5_HUMAN	ETLALLSTHR	2	1	y4	570.82	500.26	575.83	510.27	20.1	35.0	23.6	1.0000	-0.381	10.0
INSR	P06213	INSR_HUMAN	EPNGLIVLYEVSYR	2	1	y6	826.44	816.39	831.44	826.40	29.3	46.0	16.4	0.7698	1.038	106.3
ITIH1	P19827	ITIH1_HUMAN	GSLVQASEANLQAAQDFVR	3	1	y7	668.68	806.42	672.01	816.42	17.9	45.7	14.4	0.9996	0.373	1000.0
ITIH2	P19823	ITIH2_HUMAN	TEVNVLPGAK	2	1	y4	514.29	372.22	518.30	380.24	18.1	30.3	9.9	0.9663	-0.789	1000.0
ITIH4	Q14624	ITIH4_HUMAN	LALDNGGLAR	2	1	y7	500.28	702.35	505.29	712.36	17.6	31.4	2.5	0.9880	-0.144	1000.0
KNG1	P01042	KNG1_HUMAN	QVVAGLNFR	2	1	y6	502.29	677.37	507.29	687.38	17.7	35.8	4.7	0.4522	-0.426	1000.0
LAMP2	P13473	LAMP2_HUMAN	IPLNDLFR	2	2	y7	494.28	437.74	499.29	442.75	17.4	42.9	24.3	0.8478	-0.100	64.0
LUM	P51884	LUM_HUMAN	ILGPLSYSK	2	1	y7	489.29	751.40	493.29	759.41	17.2	34.9	4.5	0.9988	-0.621	1000.0
MBL2	P11226	MBL2_HUMAN	FQASVATPR	2	1	y7	488.76	701.39	493.77	711.40	17.2	27.6	7.7	0.8026	-0.431	200.5
MTDH	Q86UE4	LYRIC_HUMAN	WNSVSPASAGK	2	1	y7	552.28	617.33	556.28	625.34	19.5	26.6	29.5	1.0000	-0.440	170.0
ORM1	P02763	A1AG1_HUMAN	SDVVYTDWK	2	1	y5	556.77	712.33	560.77	720.34	19.6	33.3	8.6	0.9671	-0.388	1000.0
PFN1	P07737	PROF1_HUMAN	STGGAPTFNVTVTK	2	1	y4	690.36	448.28	694.37	456.29	24.4	50.3	25.2	0.9940	-0.169	53.6
PLA2G7	Q13093	PAFA_HUMAN	ASLAFLQK	2	1	y5	439.26	606.36	443.27	614.38	15.4	35.7	1.7	0.2825	-0.978	10.0
PLG	P00747	PLMN_HUMAN	EAQLPVIENK	2	1	y6	570.82	699.40	574.82	707.42	20.1	32.2	3.1	0.9987	-0.255	94.7
PON1	P27169	PON1_HUMAN	IQNILTEEPK	2	1	y8	592.83	943.51	596.84	951.52	20.9	33.5	11.3	0.6315	-0.929	1000.0
PRDX2	P32119	PRDX2_HUMAN	TDEGIAYR	2	1	y6	462.72	708.37	467.73	718.38	16.3	24.9	10.4	0.9996	-0.001	11.0
PROC	P04070	PROC_HUMAN	TFVLNFIK	2	1	y6	491.29	733.46	495.30	741.47	17.3	46.1	24.3	0.9698	-0.563	22.9
PROS1	P07225	PROS_HUMAN	VYFAGFPR	2	1	y6	478.75	694.37	483.76	704.38	16.8	38.8	8.5	0.6383	-0.276	591.9
RBP4	P02753	RET4_HUMAN	YWGVASFLQK	2	1	y8	599.82	849.48	603.82	857.50	21.2	46.1	3.2	0.8216	0.072	1000.0
S100A9	P06702	S10A9_HUMAN	LGHPDTLNQGEFK	3	1	b3	485.91	308.17	488.58	308.17	10.7	29.4	14.2	1.0000	-0.747	66.0
SELL	P14151	LYAM1_HUMAN	AEIEYLEK	2	1	y6	497.76	794.43	501.77	802.44	17.5	30.9	1.5	0.2602	-0.075	364.2
SERPINA4	P29622	KAIN_HUMAN	WADLSGITK	2	1	y7	495.77	733.41	499.77	741.42	17.5	35.8	8.8	1.0000	-0.185	242.5
SERPINA6	P08185	CBG_HUMAN	ITQDAQLK	2	1	y6	458.76	702.38	462.77	710.39	16.1	23.1	10.4	0.9843	-0.272	1000.0
SERPINC1	P01008	ANT3_HUMAN	LQPLDFK	2	1	y5	430.75	619.34	434.75	627.36	15.1	35.3	8.2	0.9092	-0.325	1000.0
SERPIND1	P05546	HEP2_HUMAN	QFPILLDFK	2	1	y7	560.82	845.51	564.83	853.53	19.8	50.1	5.7	0.9978	-0.237	277.6
SERPINF1	P36955	PEDF_HUMAN	LQSLFDSPDFSK	2	1	b2	692.34	242.15	696.35	242.15	24.5	41.2	6.0	0.9937	-0.138	167.4
SERPING1	P05155	IC1_HUMAN	FQPTLLTLPR	2	1	y8	593.35	910.57	598.36	920.58	21.0	43.8	9.8	0.7165	-0.422	1000.0
SHBG	P04278	SHBG_HUMAN	TSSSFEVR	2	1	y6	456.72	724.36	461.73	734.37	16.1	25.3	16.4	0.9560	0.154	83.9
SLC4A1	P02730	B3AT_HUMAN	LSVPDGFK	2	1	y7	431.74	749.38	435.74	757.40	15.2	34.5	6.7	0.8329	-0.408	764.4
SSB	P05455	LA_HUMAN	IGCLLK	2	1	y5	352.21	590.33	356.22	598.35	12.3	29.2	11.3	0.0002	-0.325	260.1
TF	P02787	TRFE_HUMAN	SASDLTWDNLK	2	1	y6	625.31	776.39	629.31	784.41	22.1	36.7	6.8	0.8666	0.233	1000.0
TPM1	P09493	TPM1_HUMAN	HIAEDADR	2	1	b2	463.72	251.15	468.72	251.15	16.3	39.5	16.0	0.0137	-0.164	375.9
TTR	P02766	TTHY_HUMAN	VLDAVR	2	1	y4	336.71	460.25	341.71	470.26	11.8	24.3	3.2	0.6249	-0.436	245.2
UMOD	P07911	UROM_HUMAN	VLNLGPITR	2	1	y7	491.81	770.45	496.81	780.46	17.3	36.9	24.6	0.6248	-0.263	10.0
VTN	P04004	VTNC_HUMAN	FEDGVLDPDYPR	2	1	y5	711.83	647.31	716.83	657.32	25.2	37.4	9.7	0.9996	-0.686	1000.0
VWF	P04275	VWF_HUMAN	VTVFPIGIGDR	2	1	y7	587.33	727.41	592.34	737.42	20.7	42.7	6.9	0.7823	0.108	23.9

Supplementary Table 3. Differentially expressed proteins between amyloid-β+ vs β–

Gene Name	Entry	Entry Name	Fold Change	Student's t-test, *P* value
A2M	P01023	A2MG_HUMAN	1.013	0.81
ADAMTS13	Q76LX8	ATS13_HUMAN	0.930	0.085
ADIPOQ	Q15848	ADIPO_HUMAN	**1.288**	**0.0048**
AFM	P43652	AFAM_HUMAN	0.957	0.26
AGT	P01019	ANGT_HUMAN	0.988	0.64
AHSG	P02765	FETUA_HUMAN	0.978	0.65
ALB	P02768	ALBU_HUMAN	0.943	0.18
ALDOA	P04075	ALDOA_HUMAN	0.862	0.13
AMBP	P02760	AMBP_HUMAN	1.010	0.97
APCS	P02743	SAMP_HUMAN	0.919	0.18
APOA1	P02647	APOA1_HUMAN	0.958	0.31
APOA2	P02652	APOA2_HUMAN	0.948	0.21
APOA4	P06727	APOA4_HUMAN	**0.895**	**0.025**
APOB	P04114	APOB_HUMAN	**1.102**	**0.031**
APOC3	P02656	APOC3_HUMAN	0.801	0.078
APOH	P02749	APOH_HUMAN	0.987	0.88
APOM	O95445	APOM_HUMAN	1.020	0.78
ASS1	P00966	ASSY_HUMAN	0.922	0.11
AZGP1	P25311	ZA2G_HUMAN	1.070	0.054
B2M	P61769	B2MG_HUMAN	**1.107**	**0.032**
BCHE	P06276	CHLE_HUMAN	1.001	0.53
BTD	P43251	BTD_HUMAN	0.960	0.23
C1QA	P02745	C1QA_HUMAN	1.027	0.89
C1QB	P02746	C1QB_HUMAN	0.996	0.79
C1QC	P02747	C1QC_HUMAN	1.013	0.70
C1R	P00736	C1R_HUMAN	0.963	0.26
C1S	P09871	C1S_HUMAN	0.989	0.64
C2	P06681	CO2_HUMAN	0.973	0.26
C3	P01024	CO3_HUMAN	0.948	0.11
C4BPA	P04003	C4BPA_HUMAN	0.957	0.74
C5	P01031	CO5_HUMAN	0.971	0.24
C7	P10643	CO7_HUMAN	0.981	0.63
C8A	P07357	CO8A_HUMAN	**0.940**	**0.038**
C8B	P07358	CO8B_HUMAN	0.956	0.13
C8G	P07360	CO8G_HUMAN	0.942	0.052
C9	P02748	CO9_HUMAN	**1.124**	**0.021**
CA1	P00915	CAH1_HUMAN	**0.856**	**0.012**
CA2	P00918	CAH2_HUMAN	0.993	0.79
CALR	P27797	CALR_HUMAN	1.059	0.15
CD14	P08571	CD14_HUMAN	1.020	0.81
CD5L	O43866	CD5L_HUMAN	0.930	0.51
CFB	P00751	CFAB_HUMAN	**0.919**	**0.027**
CFH	P08603	CFAH_HUMAN	0.964	0.66
CFHR3	Q02985	FHR3_HUMAN	1.103	0.76
CFI	P05156	CFAI_HUMAN	0.972	0.31
CLU	P10909	CLUS_HUMAN	0.964	0.077
COL10A1	Q03692	COAA1_HUMAN	0.967	0.83
COL1A1	P02452	CO1A1_HUMAN	1.054	0.50

COMP	P49747	COMP_HUMAN	0.958	0.43
CP	P00450	CERU_HUMAN	1.023	0.43
CPN1	P15169	CBPN_HUMAN	0.965	0.25
CPN2	P22792	CPN2_HUMAN	0.972	0.49
CRP	P02741	CRP_HUMAN	1.768	0.80
CST3	P01034	CYTC_HUMAN	1.055	0.33
DES	P17661	DESM_HUMAN	0.971	0.39
DSG3	P32926	DSG3_HUMAN	0.888	0.73
EXOSC10	Q01780	EXOSX_HUMAN	0.931	0.35
F10	P00742	FA10_HUMAN	0.942	0.051
F11	P03951	FA11_HUMAN	0.960	0.29
F12	P00748	FA12_HUMAN	0.944	0.35
F13A1	P00488	F13A_HUMAN	**0.908**	**0.0094**
F13B	P05160	F13B_HUMAN	**0.906**	**0.017**
F2	P00734	THRB_HUMAN	0.973	0.31
F5	P12259	FA5_HUMAN	0.977	0.40
F9	P00740	FA9_HUMAN	1.005	0.76
FBLN1	P23142	FBLN1_HUMAN	1.018	0.63
FGA	P02671	FIBA_HUMAN	**0.945**	**0.042**
FGB	P02675	FIBB_HUMAN	0.943	0.077
FGG	P02679	FIBG_HUMAN	0.960	0.18
FN1	P02751	FINC_HUMAN	0.960	0.17
GC	P02774	VTDB_HUMAN	0.955	0.20
GPX3	P22352	GPX3_HUMAN	1.007	0.80
GSN	P06396	GELS_HUMAN	1.041	0.25
HBA1	P69905	HBA_HUMAN	1.365	0.38
HP	P00738	HPT_HUMAN	1.509	0.99
HPX	P02790	HEMO_HUMAN	0.979	0.33
HRG	P04196	HRG_HUMAN	0.957	0.35
ICAM1	P05362	ICAM1_HUMAN	1.355	0.92
IDS	P22304	IDS_HUMAN	1.156	0.20
IGF2	P01344	IGF2_HUMAN	0.950	0.43
IGFBP3	P17936	IBP3_HUMAN	**0.900**	**0.019**
IGFBP5	P24593	IBP5_HUMAN	0.991	0.59
IGHM	P01871	IGHM_HUMAN	0.987	0.47
IL5	P05113	IL5_HUMAN	**0.873**	**0.046**
INSR	P06213	INSR_HUMAN	1.288	0.70
ITIH1	P19827	ITIH1_HUMAN	1.013	0.86
ITIH2	P19823	ITIH2_HUMAN	0.964	0.29
ITIH4	Q14624	ITIH4_HUMAN	0.988	0.60
KNG1	P01042	KNG1_HUMAN	0.996	0.80
LAMP2	P13473	LAMP2_HUMAN	1.017	0.60
LUM	P51884	LUM_HUMAN	1.057	0.24
MBL2	P11226	MBL2_HUMAN	1.078	0.80
MTDH	Q86UE4	LYRIC_HUMAN	**1.448**	**0.00020**
ORM1	P02763	A1AG1_HUMAN	1.034	0.82
PFN1	P07737	PROF1_HUMAN	1.280	0.092
PLA2G7	Q13093	PAFA_HUMAN	1.108	0.12
PLG	P00747	PLMN_HUMAN	0.977	0.37
PON1	P27169	PON1_HUMAN	**0.929**	**0.0067**
PRDX2	P32119	PRDX2_HUMAN	0.931	0.062
PROC	P04070	PROC_HUMAN	0.947	0.18
PROS1	P07225	PROS_HUMAN	1.001	0.96

RBP4	P02753	RET4_HUMAN	**0.904**	**0.034**
S100A9	P06702	S10A9_HUMAN	0.973	0.15
SELL	P14151	LYAM1_HUMAN	0.966	0.41
SERPINA4	P29622	KAIN_HUMAN	**0.916**	**0.031**
SERPINA6	P08185	CBG_HUMAN	0.969	0.38
SERPINC1	P01008	ANT3_HUMAN	1.004	0.76
SERPIND1	P05546	HEP2_HUMAN	0.940	0.077
SERPINF1	P36955	PEDF_HUMAN	0.949	0.11
SERPING1	P05155	IC1_HUMAN	0.978	0.64
SHBG	P04278	SHBG_HUMAN	1.138	0.14
SLC4A1	P02730	B3AT_HUMAN	1.052	0.21
SSB	P05455	LA_HUMAN	0.995	0.91
TF	P02787	TRFE_HUMAN	**0.852**	**0.043**
TPM1	P09493	TPM1_HUMAN	0.980	0.29
TTR	P02766	TTHY_HUMAN	0.999	0.92
UMOD	P07911	UROM_HUMAN	1.004	0.82
VTN	P04004	VTNC_HUMAN	0.948	0.079
VWF	P04275	VWF_HUMAN	0.934	0.20

Supplementary Table 4. Selection probability of 18-proteins in the Nested Cross Validation for AD model

Gene Name	Inner.loop1	Inner.loop2	Inner.loop3	Inner.loop4	Inner.loop5	Selection Probability
ADIPOQ	Yes	Yes	Yes	Yes	Yes	**100%**
APOA4	Yes	Yes	Yes	Yes	No	**80%**
APOB	Yes	Yes	Yes	Yes	Yes	**100%**
B2M	No	Yes	Yes	No	Yes	60%
C8A	Yes	Yes	Yes	No	No	60%
C9	Yes	Yes	Yes	No	Yes	**80%**
CA1	Yes	No	Yes	Yes	Yes	**80%**
CFB	Yes	No	Yes	No	No	40%
F13A1	Yes	No	Yes	Yes	Yes	**80%**
F13B	Yes	No	Yes	Yes	No	60%
FGA	Yes	Yes	Yes	No	Yes	**80%**
IGFBP3	No	No	Yes	Yes	No	40%
IL5	No	No	Yes	Yes	Yes	60%
MTDH	Yes	Yes	Yes	Yes	Yes	**100%**
PON1	Yes	No	Yes	No	No	40%
RBP4	Yes	No	Yes	Yes	Yes	**80%**
SERPINA4	Yes	Yes	Yes	No	No	60%
TF	No	Yes	Yes	Yes	Yes	**80%**
Accuracy	78.38%	81.08%	67.57%	67.57%	70.27%	

Supplementary Table 5. Comparison of characteristics of AD prediction models

Comparison	Characteristics	APOE ε4 carriers (CV)			10 proteins (Nested CV)[&]			10 proteins (CV)[*]			10 proteins with APOE ε4 carriers (CV)		
		Mean	Lower 95% CI	Upper 95% CI	Mean	Lower 95% CI	Upper 95% CI	Mean	Lower 95% CI	Upper 95% CI	Mean	Lower 95% CI	Upper 95% CI
Amyloid β– vs. Amyloid β+	Sensitivity (%)	68.9	59.7	78.1	69.8	66.1	73.5	84.9	80.5	89.3	89.9	88.6	91.3
Amyloid β– vs. Amyloid β+	Specificity (%)	64.4	46.7	82.2	82.2	73.9	90.5	73.6	65.1	82.0	69.8	63.7	75.8
Amyloid β– vs. Amyloid β+	PPV (%)	86.9	81.6	92.3	93.1	89.9	96.2	91.3	89.0	93.5	90.0	88.0	92.0
Amyloid β– vs. Amyloid β+	NPV (%)	41.1	36.2	46.0	47.7	43.8	51.6	63.7	58.6	68.8	69.6	66.4	72.9
Amyloid β– vs. Amyloid β+	Accuracy (%)	67.4	62.3	72.6	73.0	70.1	75.8	82.2	79.4	84.9	84.9	82.8	87.0
Amyloid β– vs. Amyloid β+	Balanced accuracy (%)	66.7	58.9	74.5	76.0	68.9	83.1	79.2	72.5	85.9	79.9	73.2	86.6
Amyloid β– vs. Amyloid β+	Equal error rate (%)	33.3	22.2	44.4	24.0	11.4	36.6	20.8	7.6	34.0	20.1	6.8	33.4
CN vs. AsymAD	Sensitivity (%)	77.5	68.3	86.8	63.7	51.5	75.9	82.4	77.4	87.4	84.8	77.7	92.0
CN vs. AsymAD	Specificity (%)	64.4	46.7	82.2	80.2	72.8	87.7	69.3	61.8	76.8	71.8	66.2	77.4
CN vs. AsymAD	PPV (%)	71.6	61.2	82.0	73.9	65.8	82.0	70.5	65.8	75.3	71.5	65.8	77.2
CN vs. AsymAD	NPV (%)	75.7	66.5	85.0	77.9	71.3	84.6	84.8	80.4	89.2	87.3	81.4	93.3
CN vs. AsymAD	Accuracy (%)	69.6	59.5	79.8	74.4	72.8	76.0	76.2	71.7	80.7	77.5	73.9	81.1
CN vs. AsymAD	Balanced accuracy (%)	71.0	63.5	78.5	71.9	64.4	79.4	75.9	68.8	83.0	78.3	71.4	85.2
CN vs. AsymAD	Equal error rate (%)	29.0	17.2	40.8	28.1	16.1	40.1	24.1	11.5	36.7	21.7	8.7	34.7

[&] Performance of the model validated by 5 test sets of outer loops

[*] Performance of the model validated by 5 fold cross validation of entire sets

Supplementary Table 6. Coefficients of the final AD prediction model

Variables	Estimate	Std.Error	Z value	Pr(>|z|)	Significance[&]
(Intercept)	1.170	0.360	3.247	0.0012	**
APOE ε4	1.665	0.527	3.158	0.0016	**
ADIPOQ	0.533	0.299	1.780	0.075	#
APOA4	-0.459	0.306	-1.498	0.13	
APOB	0.460	0.258	1.780	0.075	#
C9	0.493	0.310	1.592	0.11	
CA1	-0.508	0.281	-1.810	0.070	#
F13A1	-0.470	0.283	-1.665	0.096	#
FGA	-1.061	0.328	-3.230	0.0012	**
MTDH	0.953	0.293	3.258	0.0011	**
RBP4	-0.158	0.277	-0.568	0.57	
TF	-0.615	0.256	-2.400	0.016	*

[&] Significance Levels are represented as "#" for P < 0.1; "*" for P < 0.05; "**" for P < 0.01; and "***" for P < 0.001.

Supplementary Table 7. Differentially expressed proteins between asymptomatic AD, prodromal AD, and AD with dementia

Gene Name	Entry	Entry Name	Student's t-test, *P* value (AsymAD vs. ProdAD)	Student's t-test, *P* value (ProdAD vs. ADD)
A2M	P01023	A2MG_HUMAN	0.17	0.12
ADAMTS13	Q76LX8	ATS13_HUMAN	0.37	0.79
ADIPOQ	Q15848	ADIPO_HUMAN	0.91	0.82
AFM	P43652	AFAM_HUMAN	0.60	0.44
AGT	P01019	ANGT_HUMAN	0.99	0.21
AHSG	P02765	FETUA_HUMAN	0.50	0.84
ALB	P02768	ALBU_HUMAN	0.46	0.73
ALDOA	P04075	ALDOA_HUMAN	0.68	0.93
AMBP	P02760	AMBP_HUMAN	0.81	0.57
APCS	P02743	SAMP_HUMAN	0.82	0.35
APOA1	P02647	APOA1_HUMAN	0.26	0.17
APOA2	P02652	APOA2_HUMAN	0.082	0.99
APOA4	P06727	APOA4_HUMAN	0.81	**0.00042**
APOB	P04114	APOB_HUMAN	**0.048**	0.77
APOC3	P02656	APOC3_HUMAN	0.19	0.83
APOH	P02749	APOH_HUMAN	0.55	0.89
APOM	O95445	APOM_HUMAN	0.37	0.48
ASS1	P00966	ASSY_HUMAN	0.53	0.11
AZGP1	P25311	ZA2G_HUMAN	**0.023**	0.74
B2M	P61769	B2MG_HUMAN	0.76	**2.24E-05**
BCHE	P06276	CHLE_HUMAN	0.35	0.33
BTD	P43251	BTD_HUMAN	0.79	0.18
C1QA	P02745	C1QA_HUMAN	0.57	0.84
C1QB	P02746	C1QB_HUMAN	0.79	0.47
C1QC	P02747	C1QC_HUMAN	0.54	0.25
C1R	P00736	C1R_HUMAN	0.51	0.84
C1S	P09871	C1S_HUMAN	0.065	0.58
C2	P06681	CO2_HUMAN	0.64	1.00
C3	P01024	CO3_HUMAN	0.19	0.52
C4BPA	P04003	C4BPA_HUMAN	0.97	0.13
C5	P01031	CO5_HUMAN	0.28	0.21
C7	P10643	CO7_HUMAN	**0.046**	0.055
C8A	P07357	CO8A_HUMAN	0.49	0.80
C8B	P07358	CO8B_HUMAN	0.40	0.79
C8G	P07360	CO8G_HUMAN	0.71	0.56
C9	P02748	CO9_HUMAN	0.76	0.099
CA1	P00915	CAH1_HUMAN	0.43	0.063
CA2	P00918	CAH2_HUMAN	0.39	0.63
CALR	P27797	CALR_HUMAN	0.28	**0.0068**
CD14	P08571	CD14_HUMAN	0.52	0.37
CD5L	O43866	CD5L_HUMAN	0.15	0.58
CFB	P00751	CFAB_HUMAN	**0.0013**	0.39
CFH	P08603	CFAH_HUMAN	0.81	0.35
CFHR3	Q02985	FHR3_HUMAN	**0.016**	0.20
CFI	P05156	CFAI_HUMAN	0.052	**0.033**
CLU	P10909	CLUS_HUMAN	0.78	0.66
COL10A1	Q03692	COAA1_HUMAN	0.47	0.63
COL1A1	P02452	CO1A1_HUMAN	0.39	0.30
COMP	P49747	COMP_HUMAN	**0.036**	0.45
CP	P00450	CERU_HUMAN	0.68	0.85
CPN1	P15169	CBPN_HUMAN	0.22	0.21
CPN2	P22792	CPN2_HUMAN	0.65	0.85
CRP	P02741	CRP_HUMAN	0.23	**0.027**
CST3	P01034	CYTC_HUMAN	**0.021**	**0.0068**
DES	P17661	DESM_HUMAN	0.42	**0.010**
DSG3	P32926	DSG3_HUMAN	**0.028**	0.065
EXOSC10	Q01780	EXOSX_HUMAN	0.49	0.26
F10	P00742	FA10_HUMAN	0.26	0.62
F11	P03951	FA11_HUMAN	0.57	0.98
F12	P00748	FA12_HUMAN	0.28	0.70
F13A1	P00488	F13A_HUMAN	**0.00014**	0.16
F13B	P05160	F13B_HUMAN	**0.021**	0.80
F2	P00734	THRB_HUMAN	0.16	0.67
F5	P12259	FA5_HUMAN	0.33	0.058
F9	P00740	FA9_HUMAN	0.46	0.34
FBLN1	P23142	FBLN1_HUMAN	0.37	0.38
FGA	P02671	FIBA_HUMAN	**0.00020**	0.44
FGB	P02675	FIBB_HUMAN	**0.023**	0.52
FGG	P02679	FIBG_HUMAN	**0.029**	0.92

FN1	P02751	FINC_HUMAN	**1.88E-08**	**4.95E-05**
GC	P02774	VTDB_HUMAN	0.19	0.78
GPX3	P22352	GPX3_HUMAN	0.91	0.14
GSN	P06396	GELS_HUMAN	0.98	0.36
HBA1	P69905	HBA_HUMAN	**0.043**	0.76
HP	P00738	HPT_HUMAN	0.71	**0.047**
HPX	P02790	HEMO_HUMAN	0.42	0.091
HRG	P04196	HRG_HUMAN	0.50	**0.049**
ICAM1	P05362	ICAM1_HUMAN	0.36	0.94
IDS	P22304	IDS_HUMAN	0.60	0.75
IGF2	P01344	IGF2_HUMAN	0.95	0.90
IGFBP3	P17936	IBP3_HUMAN	0.52	0.30
IGFBP5	P24593	IBP5_HUMAN	0.65	0.81
IGHM	P01871	IGHM_HUMAN	0.19	0.45
IL5	P05113	IL5_HUMAN	0.40	0.42
INSR	P06213	INSR_HUMAN	0.72	0.91
ITIH1	P19827	ITIH1_HUMAN	0.23	0.71
ITIH2	P19823	ITIH2_HUMAN	0.75	0.50
ITIH4	Q14624	ITIH4_HUMAN	0.11	0.12
KNG1	P01042	KNG1_HUMAN	0.95	0.85
LAMP2	P13473	LAMP2_HUMAN	0.51	**9.34E-05**
LUM	P51884	LUM_HUMAN	0.42	0.56
MBL2	P11226	MBL2_HUMAN	0.36	0.91
MTDH	Q86UE4	LYRIC_HUMAN	**7.38E-07**	0.49
ORM1	P02763	A1AG1_HUMAN	0.80	**0.00067**
PFN1	P07737	PROF1_HUMAN	**0.0021**	0.20
PLA2G7	Q13093	PAFA_HUMAN	0.33	0.24
PLG	P00747	PLMN_HUMAN	0.80	0.67
PON1	P27169	PON1_HUMAN	0.088	0.50
PRDX2	P32119	PRDX2_HUMAN	0.16	0.11
PROC	P04070	PROC_HUMAN	0.48	0.36
PROS1	P07225	PROS_HUMAN	0.86	0.90
RBP4	P02753	RET4_HUMAN	0.81	**0.0028**
S100A9	P06702	S10A9_HUMAN	0.059	0.63
SELL	P14151	LYAM1_HUMAN	0.57	**0.029**
SERPINA4	P29622	KAIN_HUMAN	0.90	0.64
SERPINA6	P08185	CBG_HUMAN	0.11	0.36
SERPINC1	P01008	ANT3_HUMAN	0.49	0.70
SERPIND1	P05546	HEP2_HUMAN	0.49	0.16
SERPINF1	P36955	PEDF_HUMAN	0.78	0.85
SERPING1	P05155	IC1_HUMAN	0.28	0.76
SHBG	P04278	SHBG_HUMAN	0.11	0.89
SLC4A1	P02730	B3AT_HUMAN	0.23	0.55
SSB	P05455	LA_HUMAN	0.53	0.76
TF	P02787	TRFE_HUMAN	**0.043**	0.36
TPM1	P09493	TPM1_HUMAN	0.82	0.96
TTR	P02766	TTHY_HUMAN	0.11	0.43
UMOD	P07911	UROM_HUMAN	0.84	**0.024**
VTN	P04004	VTNC_HUMAN	**0.047**	0.89
VWF	P04275	VWF_HUMAN	0.14	0.82

Supplementary Table 8. Selection probability of 32-proteins in the Nested Cross Validation for APM model

Gene Name	Inner.loop1	Inner.loop2	Inner.loop3	Inner.loop4	Inner.loop5	Selection Probability
APOA4	Yes	Yes	Yes	Yes	Yes	**100%**
APOB	No	Yes	Yes	Yes	No	60%
AZGP1	No	No	No	Yes	No	20%
B2M	Yes	Yes	Yes	Yes	Yes	**100%**
C7	No	Yes	No	Yes	No	40%
CALR	Yes	No	Yes	Yes	Yes	**80%**
CFB	Yes	No	Yes	Yes	No	60%
CFHR3	No	Yes	Yes	Yes	No	60%
CFI	No	Yes	Yes	Yes	No	60%
COMP	No	No	Yes	No	No	20%
CRP	No	No	Yes	No	No	20%
CST3	Yes	No	No	No	No	20%
DES	No	Yes	Yes	Yes	Yes	**80%**
DSG3	No	No	No	No	No	0%
F13A1	Yes	Yes	Yes	Yes	Yes	**100%**
F13B	No	No	No	No	No	0%
FGA	Yes	Yes	Yes	Yes	Yes	**100%**
FGB	No	No	Yes	No	No	20%
FGG	No	Yes	Yes	No	No	40%
FN1	Yes	Yes	Yes	Yes	Yes	**100%**
HBA1	No	No	No	No	No	0%
HP	No	No	No	No	No	0%
HRG	No	No	Yes	Yes	No	40%
LAMP2	Yes	Yes	Yes	Yes	Yes	**100%**
MTDH	Yes	Yes	Yes	Yes	Yes	**100%**
ORM1	Yes	No	Yes	Yes	Yes	**80%**
PFN1	Yes	Yes	Yes	Yes	No	**80%**
RBP4	No	Yes	Yes	Yes	No	60%
SELL	No	Yes	No	Yes	No	40%
TF	No	No	No	Yes	Yes	40%
UMOD	No	No	No	Yes	No	20%
VTN	No	Yes	No	No	No	20%
Accuracy	67.86%	67.86%	77.78%	71.43%	78.57%	

Supplementary Table 9. Comparison of characteristics of the 4 AD progression monitoring models

Comparison	Characteristics	APOE ε4 carriers (CV)			K-MMSE score (CV)			10 proteins (Nested CV)[&]			10 proteins (CV)[*]			10 proteins with K-MMSE (CV)		
		Mean	Lower 95% CI	Uppper 95% CI	Mean	Lower 95% CI	Uppper 95% CI	Mean	Lower 95% CI	Uppper 95% CI	Mean	Lower 95% CI	Uppper 95% CI	Mean	Lower 95% CI	Uppper 95% CI
AsymAD vs. ProdAD + ADD	Sensitivity (%)	100.0	100.0	100.0	96.0	93.6	98.4	89.0	87.1	90.9	89.0	85.3	92.7	89.0	86.1	91.9
AsymAD vs. ProdAD + ADD	Specificity (%)	0.0	0.0	0.0	20.7	14.2	27.3	69.3	66.4	72.1	66.8	62.0	71.6	72.5	63.3	81.7
AsymAD vs. ProdAD + ADD	PPV (%)	72.0	71.4	72.5	75.8	74.5	77.1	88.1	87.0	89.3	87.4	85.8	88.9	89.7	86.5	93.0
AsymAD vs. ProdAD + ADD	NPV (%)	NA	NA	NA	77.5	65.7	89.3	71.5	67.6	75.5	73.0	65.4	80.6	73.4	69.1	77.8
AsymAD vs. ProdAD	Sensitivity (%)	100.0	100.0	100.0	90.6	84.8	96.4	87.4	80.6	94.3	87.1	81.8	92.4	83.3	77.4	89.1
AsymAD vs. ProdAD	Specificity (%)	0.0	0.0	0.0	21.7	14.8	28.6	76.1	69.7	82.4	78.6	74.6	82.6	81.8	75.9	87.7
AsymAD vs. ProdAD	PPV (%)	50.8	50.0	51.5	55.8	54.0	57.6	80.5	75.2	85.8	85.3	83.1	87.4	87.5	83.8	91.2
AsymAD vs. ProdAD	NPV (%)	NA	NA	NA	77.5	65.7	89.3	86.7	80.4	92.9	83.6	76.8	90.3	80.7	74.1	87.3
AsymAD vs. ADD	Sensitivity (%)	100.0	100.0	100.0	100.0	100.0	100.0	85.9	81.7	90.2	88.3	84.3	92.3	93.3	90.5	96.1
AsymAD vs. ADD	Specificity (%)	0.0	0.0	0.0	91.7	84.2	99.1	91.7	86.4	96.9	81.9	76.0	87.8	84.3	77.3	91.3
AsymAD vs. ADD	PPV (%)	58.8	51.9	65.6	97.8	95.6	100.0	93.8	89.7	97.8	86.5	82.6	90.4	90.7	86.5	94.8
AsymAD vs. ADD	NPV (%)	NA	NA	NA	100.0	100.0	100.0	82.4	77.1	87.7	84.3	80.1	88.5	91.0	87.1	94.9
ProdAD vs. ADD	Sensitivity (%)	38.0	36.0	40.0	78.0	73.1	82.9	84.1	78.2	90.0	84.3	79.8	88.9	91.3	87.2	95.5
ProdAD vs. ADD	Specificity (%)	50.0	40.5	59.5	80.5	78.5	82.5	81.4	75.4	87.4	91.0	87.1	94.9	93.3	88.9	97.8
ProdAD vs. ADD	PPV (%)	45.1	39.8	50.4	81.1	78.9	83.4	84.1	79.5	88.6	91.1	87.2	95.1	94.5	90.9	98.2
ProdAD vs. ADD	NPV (%)	42.9	37.4	48.4	77.7	74.1	81.3	85.7	81.2	90.1	86.0	82.0	90.0	91.2	87.1	95.4
AsymAD vs. ProdAD vs. ADD	Accuracy (%)	31.6	28.0	35.2	60.4	59.3	61.6	72.7	70.4	75.0	74.8	73.0	76.7	79.1	77.8	80.5

[&] Performance of the model validated by 5 test sets of outer loops

[*] Performance of the model validated by 5 fold cross validation of entire sets

Supplementary Table 10. Coefficients of the final AD progression monitoring model

10 Proteins with K-MMSE	Coefficient, ProdAD	Standard error, ProdAD	Z value, ProdAD	*P* value, ProdAD	Coefficient, ADD	Standard error, ADD	Z value, ADD	*P* value, ADD
(Intercept)	20.031	7.486	2.676	0.0075	32.587	8.441	3.861	0.00011
K-MMSE	-0.732	0.276	-2.651	0.0080	-1.382	0.346	-3.994	0.000065
APOA4	-0.562	0.672	-0.837	0.40	-3.178	1.105	-2.877	0.0040
B2M	-0.049	0.617	-0.080	0.94	1.986	0.835	2.379	0.017
CALR	-1.040	0.529	-1.964	0.049	0.725	0.773	0.938	0.35
DES	0.288	0.482	0.597	0.55	-0.887	0.787	-1.128	0.26
F13A1	2.177	0.726	2.999	0.0027	2.219	0.838	2.649	0.0081
FGA	1.333	0.783	1.702	0.089	0.350	0.928	0.378	0.71
FN1	-3.065	0.846	-3.622	0.00029	-0.871	0.938	-0.929	0.35
LAMP2	0.476	0.531	0.896	0.37	2.801	0.975	2.872	0.0041
MTDH	0.709	0.808	0.877	0.38	2.319	1.194	1.943	0.052
ORM1	-1.422	0.672	-2.117	0.034	0.252	0.898	0.280	0.78

* AsymAD (Asymtomatic AD) groups were designated as the reference group

Supplementary Table 11. Gene ontology (GO) analysis using 312 proteins from the three consensus network modules (M2, M5, and M8) from Johnson et al. brain cohorts.

Category	Term	Count	Percent (%)	Proteins	Fold Enrichment	EASE score	FDR	TF (M2 Blue)	B2M (M5 Green)	F13A1 (M5 Green)	CA1 (M8 Pink)	FGA (M8 Pink)	ORM1 (M8 Pink)
GOTERM_CC_DIRECT	GO:0070062~extracellular exosome	189	61.0	P04114, Q9BW	4.0	8.0E-76	2.4E-73	Yes	Yes	No	Yes	Yes	Yes
GOTERM_CC_DIRECT	GO:0072562~blood microparticle	42	13.5	P04433, P0267	16.3	6.1E-38	8.9E-36	Yes	No	Yes	No	Yes	Yes
UP_KEYWORDS	Acetylation	134	43.2	P04114, Q9BW	2.6	2.3E-28	6.8E-26	No	No	Yes	Yes	No	No
GOTERM_CC_DIRECT	GO:0005615~extracellular space	78	25.2	Q96DZ9, P041	3.4	4.1E-22	3.0E-20	Yes	Yes	No	No	Yes	Yes
GOTERM_CC_DIRECT	GO:0005576~extracellular region	84	27.1	P04433, P041	3.1	4.9E-21	2.4E-19	Yes	Yes	Yes	No	Yes	Yes
GOTERM_CC_DIRECT	GO:0005925~focal adhesion	42	13.5	P50395, P365	6.3	4.9E-21	2.4E-19	No	Yes	No	No	No	No
GOTERM_CC_DIRECT	GO:0005829~cytosol	126	40.6	P04114, Q142	2.2	1.4E-20	5.0E-19	No	No	No	Yes	No	No
UP_KEYWORDS	Phosphoprotein	204	65.8	P04114, Q142	1.6	3.1E-20	4.6E-18	Yes	No	No	No	Yes	No
UP_KEYWORDS	Cytoplasm	145	46.8	P04114, Q9BW	2.0	1.3E-19	1.3E-17	No	No	Yes	Yes	No	No
UP_KEYWORDS	Disease mutation	93	30.0	P04114, P024	2.4	2.8E-16	2.1E-14	Yes	Yes	Yes	No	Yes	No
UP_KEYWORDS	Cytoskeleton	58	18.7	Q9BW30, Q14	3.4	6.3E-16	3.7E-14	No	No	No	No	No	No
UP_KEYWORDS	Actin-binding	29	9.4	Q9NVD7, Q8T	7.0	1.3E-15	6.4E-14	No	No	No	No	No	No
GOTERM_BP_DIRECT	**GO:0002576~platelet degranulation**	21	6.8	P01011, P0267	11.5	1.3E-15	2.3E-12	Yes	No	Yes	No	Yes	Yes
GOTERM_CC_DIRECT	GO:0005913~cell-cell adherens junction	31	10.0	Q9NVD7, P33	5.7	3.6E-14	1.2E-12	No	No	No	No	No	No
GOTERM_MF_DIRECT	GO:0098641~cadherin binding involved in cell-cell adhesion	30	9.7	Q9NVD7, P33	5.8	4.5E-14	2.1E-11	No	No	No	No	No	No
UP_KEYWORDS	Secreted	75	24.2	P04433, P041	2.5	6.3E-14	2.3E-12	Yes	Yes	Yes	No	Yes	Yes
GOTERM_MF_DIRECT	GO:0003779~actin binding	25	8.1	Q86T65, Q9N	5.1	1.7E-10	2.6E-08	No	No	No	No	No	No
GOTERM_CC_DIRECT	**GO:0031093~platelet alpha granule lumen**	12	3.9	P01011, P0267	12.9	1.7E-09	3.7E-08	No	No	Yes	No	Yes	Yes
GOTERM_BP_DIRECT	GO:0098609~cell-cell adhesion	23	7.4	P53990, P077	4.8	3.1E-09	1.1E-06	No	No	No	No	No	No
GOTERM_MF_DIRECT	GO:0005515~protein binding	204	65.8	P04114, Q142	1.3	1.5E-08	1.1E-06	Yes	Yes	No	Yes	Yes	Yes
GOTERM_BP_DIRECT	**GO:0006953~acute-phase response**	10	3.2	P01011, P007	14.4	2.0E-08	5.1E-06	No	No	No	No	No	Yes
GOTERM_CC_DIRECT	GO:0005856~cytoskeleton	25	8.1	Q14764, P004	4.0	2.2E-08	4.0E-07	No	No	No	No	No	No
GOTERM_CC_DIRECT	GO:0016020~membrane	72	23.2	Q14204, Q142	1.9	4.4E-08	7.1E-07	No	Yes	No	No	No	No
UP_KEYWORDS	**Acute phase**	7	2.3	P01011, P007	24.5	2.5E-07	6.8E-06	No	No	No	No	No	Yes
UP_KEYWORDS	Disulfide bond	87	28.1	P04433, P041	1.7	5.2E-07	1.1E-05	Yes	Yes	No	No	Yes	Yes
UP_KEYWORDS	Glycation	6	1.9	G3V1N2, P688	33.2	5.4E-07	1.1E-05	No	Yes	No	No	No	No
UP_SEQ_FEATURE	disulfide bond	78	25.2	P04433, P041	1.7	6.0E-07	8.3E-04	Yes	Yes	No	No	Yes	Yes
GOTERM_CC_DIRECT	GO:0005737~cytoplasm	128	41.3	P04114, Q9BW	1.4	1.3E-06	1.7E-05	No	Yes	No	Yes	No	No
UP_SEQ_FEATURE	signal peptide	84	27.1	P04433, P041	1.6	2.5E-06	1.1E-03	Yes	Yes	No	No	Yes	Yes
UP_KEYWORDS	**Amyloidosis**	7	2.3	P02671, O146	16.6	3.1E-06	4.6E-05	No	Yes	No	No	Yes	No
GOTERM_CC_DIRECT	GO:0009897~external side of plasma membrane	16	5.2	P02671, Q995	4.4	3.6E-06	4.1E-05	No	Yes	No	No	Yes	No
GOTERM_CC_DIRECT	GO:0005788~endoplasmic reticulum lumen	15	4.8	P04114, P024	4.6	5.1E-06	5.5E-05	No	Yes	No	No	No	No
UP_KEYWORDS	Glycoprotein	103	33.2	P04114, P024	1.5	5.9E-06	8.3E-05	Yes	Yes	Yes	No	Yes	Yes
GOTERM_MF_DIRECT	GO:0015485~cholesterol binding	8	2.6	P05090, Q9BX	11.0	6.8E-06	3.2E-04	No	No	No	No	No	No
GOTERM_BP_DIRECT	GO:0042493~response to drug	19	6.1	P02452, P299	3.5	8.3E-06	1.0E-03	No	Yes	No	No	No	No
UP_KEYWORDS	**Pyrrolidone carboxylic acid**	10	3.2	P02452, P050	7.1	1.1E-05	1.4E-04	No	Yes	No	No	No	Yes
UP_KEYWORDS	Lipoprotein	31	10.0	P04114, Q094	2.4	1.4E-05	1.8E-04	No	No	No	No	No	No
INTERPRO	IPR000566:Lipocalin/cytosolic fatty-acid binding protein domain	7	2.3	P41222, P050	12.5	1.7E-05	4.3E-03	No	No	No	No	No	Yes
INTERPRO	IPR012674:Calycin	7	2.3	P41222, P050	11.5	2.8E-05	4.3E-03	No	No	No	No	No	Yes
UP_KEYWORDS	**Amyloid**	6	1.9	P02671, P026	15.9	3.1E-05	3.5E-04	No	Yes	No	No	Yes	No
INTERPRO	IPR011038:Calycin-like	7	2.3	P41222, P050	10.9	3.8E-05	4.3E-03	No	No	No	No	No	Yes
UP_KEYWORDS	**Neurodegeneration**	16	5.2	Q14204, Q142	3.6	4.0E-05	4.1E-04	No	No	No	No	No	No
GOTERM_CC_DIRECT	GO:0005886~plasma membrane	101	32.6	P04114, Q8W	1.4	4.0E-05	3.3E-04	No	Yes	No	No	Yes	No
GOTERM_CC_DIRECT	GO:0005938~cell cortex	11	3.5	P02671, Q96A	5.3	4.6E-05	3.6E-04	No	No	No	No	Yes	No
UP_KEYWORDS	Signal	92	29.7	P04433, P041	1.5	6.3E-05	5.7E-04	Yes	Yes	No	No	Yes	Yes
UP_KEYWORDS	Immunoglobulin domain	22	7.1	P04433, Q9GZ	2.7	8.6E-05	7.5E-04	No	Yes	No	No	No	No
GOTERM_BP_DIRECT	GO:0001523~retinoid metabolic process	8	2.6	P04114, P026	7.4	9.7E-05	7.7E-03	No	No	No	No	No	No
UP_KEYWORDS	Transport	50	16.1	P04114, Q142	1.7	3.2E-04	2.6E-03	Yes	No	No	No	No	Yes
GOTERM_MF_DIRECT	GO:0042802~identical protein binding	28	9.0	P02452, Q147	2.1	3.8E-04	1.0E-02	No	Yes	No	No	No	No
INTERPRO	IPR003006:Immunoglobulin/major histocompatibility complex, conserved site	7	2.3	P16112, P018	7.1	4.4E-04	2.5E-02	No	Yes	No	No	No	No
GOTERM_BP_DIRECT	GO:0042157~lipoprotein metabolic process	6	1.9	P04114, P027	8.9	5.2E-04	2.4E-02	No	No	No	No	No	No
GOTERM_MF_DIRECT	GO:0005543~phospholipid binding	8	2.6	P04114, Q9BX	5.2	8.3E-04	1.9E-02	No	No	No	No	No	No
GOTERM_BP_DIRECT	GO:0042632~cholesterol homeostasis	7	2.3	P04114, P026	6.2	9.2E-04	3.7E-02	No	No	No	No	No	No
GOTERM_BP_DIRECT	GO:0010903~negative regulation of very-low-density lipoprotein particle remodeling	3	1.0	P02647, P026	56.3	9.2E-04	3.7E-02	No	No	No	No	No	No
GOTERM_MF_DIRECT	GO:0070653~high-density lipoprotein particle receptor binding	3	1.0	P02647, P026	56.3	9.3E-04	2.0E-02	No	No	No	No	No	No
INTERPRO	IPR013783:Immunoglobulin-like fold	30	9.7	P04433, P209	1.9	9.9E-04	4.5E-02	No	Yes	Yes	No	No	No
GOTERM_MF_DIRECT	GO:0042803~protein homodimerization activity	26	8.4	O14672, Q995	2.0	1.3E-03	2.3E-02	No	No	No	No	No	No

GOTERM_MF_DIRECT	GO:0017048~Rho GTPase binding	5	1.6	Q86T65, P0773	10.0	1.4E-03	2.3E-02	No	No	No	No	No	No
GOTERM_MF_DIRECT	GO:0008289~lipid binding	10	3.2	P60903, P0509	3.7	1.5E-03	2.4E-02	No	No	No	No	No	No
GOTERM_CC_DIRECT	GO:0042627~chylomicron	4	1.3	P04114, P0264	16.9	1.5E-03	8.1E-03	No	No	No	No	No	No
UP_KEYWORDS	Isopeptide bond	31	10.0	P02671, Q1476	1.8	1.9E-03	1.2E-02	No	No	No	No	Yes	No
GOTERM_BP_DIRECT	GO:0050821~protein stabilization	9	2.9	P13473, O0029	3.7	2.9E-03	8.8E-02	No	No	No	No	No	No
UP_SEQ_FEATURE	glycosylation site:N-linked (GlcNAc...)	86	27.7	P04114, P0245	1.3	3.1E-03	1.3E-01	Yes	No	Yes	No	Yes	Yes
GOTERM_MF_DIRECT	GO:0005102~receptor binding	15	4.8	P02671, P0773	2.4	4.3E-03	5.9E-02	No	No	No	No	Yes	No
GOTERM_BP_DIRECT	GO:0048260~positive regulation of receptor-mediated endocytosis	4	1.3	O00291, P0278	11.9	4.3E-03	1.2E-01	Yes	Yes	No	No	No	No
GOTERM_CC_DIRECT	GO:0034361~very-low-density lipoprotein particle	4	1.3	P04114, P0264	11.8	4.4E-03	1.9E-02	No	No	No	No	No	No
GOTERM_MF_DIRECT	GO:0055102~lipase inhibitor activity	3	1.0	P02647, P0265	28.1	4.5E-03	5.9E-02	No	No	No	No	No	No
GOTERM_BP_DIRECT	GO:0044267~cellular protein metabolic process	8	2.6	P02671, P8424	3.8	5.0E-03	1.3E-01	No	Yes	No	No	Yes	No
GOTERM_MF_DIRECT	GO:0005319~lipid transporter activity	4	1.3	P04114, P0509	11.3	5.0E-03	6.5E-02	No	No	No	No	No	No
INTERPRO	IPR003597:Immunoglobulin C1-set	6	1.9	P01859, P0187	5.4	5.1E-03	1.3E-01	No	Yes	No	No	No	No
GOTERM_BP_DIRECT	GO:0001895~retina homeostasis	5	1.6	P02768, P0187	7.0	5.3E-03	1.3E-01	Yes	Yes	No	No	No	No
GOTERM_MF_DIRECT	GO:0001948~glycoprotein binding	6	1.9	Q02246, P0867	5.2	5.9E-03	7.1E-02	No	Yes	No	No	No	No
SMART	SM00407:IGc1	6	1.9	P01859, P0187	5.1	6.3E-03	1.4E-01	No	Yes	No	No	No	No
GOTERM_CC_DIRECT	GO:0005794~Golgi apparatus	26	8.4	P04114, P0245	1.8	6.3E-03	2.5E-02	No	Yes	No	No	No	No
GOTERM_BP_DIRECT	GO:0008203~cholesterol metabolic process	6	1.9	P04114, Q0546	5.0	7.0E-03	1.6E-01	No	No	No	No	No	No
GOTERM_CC_DIRECT	**GO:0043005~neuron projection**	11	3.5	P09417, P2352	2.7	7.2E-03	2.8E-02	No	No	No	No	No	No
GOTERM_CC_DIRECT	GO:0034366~spherical high-density lipoprotein particle	3	1.0	P02647, P0265	22.1	7.5E-03	2.8E-02	No	No	No	No	No	No
UP_KEYWORDS	Oxidation	4	1.3	P02647, P6273	9.8	7.5E-03	4.1E-02	No	No	No	No	No	No
INTERPRO	IPR003599:Immunoglobulin subtype	17	5.5	P04433, Q9GZ	2.1	8.2E-03	1.7E-01	No	Yes	No	No	No	No
INTERPRO	IPR007110:Immunoglobulin-like domain	23	7.4	P04433, Q9GZ	1.8	8.5E-03	1.7E-01	No	Yes	No	No	No	No
INTERPRO	IPR013106:Immunoglobulin V-set	15	4.8	P04433, Q9GZ	2.2	9.0E-03	1.7E-01	No	Yes	No	No	No	No
GOTERM_BP_DIRECT	GO:0033344~cholesterol efflux	4	1.3	P04114, P0264	9.0	9.4E-03	1.9E-01	No	No	No	No	No	No
GOTERM_BP_DIRECT	GO:0042158~lipoprotein biosynthetic process	3	1.0	P04114, P0264	18.8	1.0E-02	2.0E-01	No	No	No	No	No	No
GOTERM_BP_DIRECT	GO:0006869~lipid transport	6	1.9	P05090, P1790	4.4	1.1E-02	2.1E-01	No	No	No	No	No	No
GOTERM_CC_DIRECT	GO:0031901~early endosome membrane	7	2.3	Q9UNH7, P040	3.6	1.4E-02	4.7E-02	No	Yes	No	No	No	No
GOTERM_BP_DIRECT	GO:0050776~regulation of immune response	9	2.9	P04433, P0245	2.8	1.4E-02	2.5E-01	No	Yes	No	No	No	No
UP_KEYWORDS	Lipid transport	6	1.9	P04114, Q9NZ	4.1	1.5E-02	7.5E-02	No	No	No	No	No	No
GOTERM_CC_DIRECT	GO:0005769~early endosome	10	3.2	P04114, P3650	2.6	1.6E-02	5.2E-02	Yes	No	No	No	No	No
SMART	SM00409:IG	17	5.5	P04433, Q9GZ	1.9	1.8E-02	1.8E-01	No	Yes	No	No	No	No
GOTERM_BP_DIRECT	**GO:0045087~innate immune response**	15	4.8	P02671, P0CG	2.0	2.1E-02	3.4E-01	No	Yes	No	No	Yes	No
GOTERM_BP_DIRECT	GO:0006641~triglyceride metabolic process	4	1.3	P04040, P0265	6.4	2.4E-02	3.7E-01	No	No	No	No	No	No
GOTERM_BP_DIRECT	GO:0033700~phospholipid efflux	3	1.0	P02647, P0265	12.1	2.5E-02	3.9E-01	No	No	No	No	No	No
KEGG_PATHWAY	hsa04810:Regulation of actin cytoskeleton	11	3.5	P18206, P3650	2.2	2.6E-02	3.7E-01	No	No	No	No	No	No
UP_KEYWORDS	HDL	3	1.0	P02647, P0DJI	11.7	2.6E-02	1.2E-01	No	No	No	No	No	No
SMART	SM00406:IGv	11	3.5	P04433, Q9GZ	2.2	2.8E-02	2.4E-01	No	Yes	No	No	No	No
GOTERM_BP_DIRECT	GO:0030036~actin cytoskeleton organization	7	2.3	P84095, Q86T6	3.0	2.8E-02	4.3E-01	No	No	No	No	No	No
GOTERM_BP_DIRECT	GO:0034375~high-density lipoprotein particle remodeling	3	1.0	P02647, P0265	11.3	2.8E-02	4.3E-01	No	No	No	No	No	No
GOTERM_MF_DIRECT	GO:0017127~cholesterol transporter activity	3	1.0	P04114, P0264	11.3	2.8E-02	2.5E-01	No	No	No	No	No	No
UP_SEQ_FEATURE	sequence variant	207	66.8	P04114, Q1420	1.1	3.0E-02	5.0E-01	Yes	Yes	Yes	Yes	Yes	Yes
UP_KEYWORDS	Host-virus interaction	12	3.9	P02790, P6293	2.1	3.2E-02	1.4E-01	No	No	No	No	No	No
INTERPRO	IPR001500:Alpha-1-acid glycoprotein	2	0.6	P19652, P0276	60.8	3.2E-02	4.6E-01	No	No	No	No	No	Yes
UP_SEQ_FEATURE	mutagenesis site	45	14.5	P04114, Q9NZ	1.3	3.5E-02	5.6E-01	No	Yes	No	No	No	No
GOTERM_BP_DIRECT	GO:0060621~negative regulation of cholesterol import	2	0.6	P02656, P0265	56.3	3.5E-02	5.1E-01	No	No	No	No	No	No
GOTERM_BP_DIRECT	GO:0051496~positive regulation of stress fiber assembly	4	1.3	P60903, P0773	5.4	3.8E-02	5.2E-01	No	No	No	No	No	No
UP_KEYWORDS	Immunity	14	4.5	P04433, P0267	1.9	4.0E-02	1.7E-01	No	Yes	No	No	Yes	No
GOTERM_BP_DIRECT	GO:0043691~reverse cholesterol transport	3	1.0	P02647, P0265	9.4	4.0E-02	5.4E-01	No	No	No	No	No	No
GOTERM_MF_DIRECT	GO:0044822~poly(A) RNA binding	29	9.4	P50395, Q1420	1.4	4.5E-02	3.6E-01	No	No	No	No	No	No
KEGG_PATHWAY	hsa05132:Salmonella infection	6	1.9	Q14204, P8409	3.1	4.6E-02	5.2E-01	No	No	No	No	No	No
OMIM_DISEASE	**105200~Amyloidosis, familial visceral**	2	0.6	P02671, P6176	38.9	5.0E-02	1.0E+00	No	Yes	No	No	Yes	No
GOTERM_BP_DIRECT	GO:0018158~protein oxidation	2	0.6	P02647, P0265	37.6	5.2E-02	6.6E-01	No	No	No	No	No	No
GOTERM_BP_DIRECT	GO:0046340~diacylglycerol catabolic process	2	0.6	Q05469, P0265	37.6	5.2E-02	6.6E-01	No	No	No	No	No	No
GOTERM_MF_DIRECT	GO:0034190~apolipoprotein receptor binding	2	0.6	P02647, P0265	37.5	5.2E-02	3.8E-01	No	No	No	No	No	No
GOTERM_CC_DIRECT	GO:0034364~high-density lipoprotein particle	3	1.0	P02647, P0DJI	8.0	5.3E-02	1.5E-01	No	No	No	No	No	No
UP_KEYWORDS	Ubl conjugation	35	11.3	Q14203, Q147	1.4	5.4E-02	2.1E-01	No	No	No	No	No	No
UP_KEYWORDS	Polymorphism	196	63.2	P04114, Q1420	1.1	5.5E-02	2.1E-01	Yes	No	Yes	Yes	Yes	Yes
PIR_SUPERFAMILY	PIRSF036899:alpha(1)-acid glycoprotein	2	0.6	P19652, P0276	34.5	5.6E-02	1.0E+00	No	No	No	No	No	Yes
UP_SEQ_FEATURE	glycosylation site:N-linked (Glc) (glycation); in vitro	2	0.6	P02768, P6176	32.7	6.0E-02	7.5E-01	No	Yes	No	No	No	No
GOTERM_BP_DIRECT	GO:0060192~negative regulation of lipase activity	2	0.6	P02647, P0265	28.2	6.9E-02	7.8E-01	No	No	No	No	No	No
UP_KEYWORDS	**Amyotrophic lateral sclerosis**	3	1.0	Q14203, P0773	6.9	7.0E-02	2.5E-01	No	No	No	No	No	No
GOTERM_BP_DIRECT	GO:0046686~response to cadmium ion	3	1.0	P04040, P6176	6.8	7.2E-02	7.9E-01	No	Yes	No	No	No	No

GOTERM_BP_DIRECT	GO:0006656~phosphatidylcholine biosynthetic process	3	1.0	Q8WWI5, P02	6.8	7.2E-02	7.9E-01	No	No	No	No	No	No
GOTERM_MF_DIRECT	GO:0005546~phosphatidylinositol-4,5-bisphosphate binding	4	1.3	O00291, P077	3.9	8.3E-02	5.3E-01	No	No	No	No	No	No
GOTERM_BP_DIRECT	GO:0030300~regulation of intestinal cholesterol absorption	2	0.6	P02647, P026	22.5	8.5E-02	8.8E-01	No	No	No	No	No	No
GOTERM_BP_DIRECT	**GO:0002682~regulation of immune system process**	2	0.6	P19652, P027	22.5	8.5E-02	8.8E-01	No	No	No	No	No	Yes
BIOCARTA	h_rhoPathway:Rho cell motility signaling pathway	4	1.3	P18206, P077	3.5	9.6E-02	1.0E+00	No	No	No	No	No	No

Supplementary Table 12. LOD and LOQ results of final target proteins in the models

Gene Name	Curve fit, slope	Curve fit, intercept	Curve fit, R^2	LOD, fmol	LLOQ, fmol
ADIPOQ	0.091	0.227	0.993	4.307	7.813
APOA4	0.010	0.004	0.998	0.154	0.244
APOB	0.008	-0.187	0.990	2.497	7.813
B2M	0.029	0.062	1.000	2.236	3.906
C9	0.013	0.002	0.999	0.128	0.977
CA1	0.438	0.046	0.999	0.114	0.488
CALR	0.305	-0.362	1.000	1.493	1.953
DES	0.456	0.040	0.999	0.123	0.244
F13A1	0.045	-1.181	0.988	26.885	31.250
FGA	0.001	-0.001	1.000	1.688	3.906
FN1	0.004	-0.002	0.998	1.904	3.906
LAMP2	1.077	-13.606	0.997	12.642	15.625
MTDH	0.161	-0.029	0.999	0.755	1.953
ORM1	0.000	0.000	0.998	1.701	1.953
RBP4	0.020	-0.071	0.995	4.010	7.813
TF	0.003	-0.045	0.999	14.552	15.625

Supplementary Table 13. Stability results for the target proteins

Gene Name	QC concentration, fmol	Replicates	6 hours	24 hours	Freeze & Thaw	Freeze & Thaw Twice	4 Weeks
ADIPOQ	23.438	1	101.5	95.2	117.1	84.6	119.9
		2	117.8	90.7	97.1	**71.1**	113.5
		3	97.6	86.5	83.8	106.8	105.5
APOA4	0.732	1	110.2	108.1	98.7	102.7	107.2
		2	101.2	94.1	104.3	100.9	117.7
		3	105.4	105.6	100.7	98.3	87.8
APOB	23.438	1	97.2	101.2	96.5	98.0	119.5
		2	100.8	101.3	99.4	98.8	102.6
		3	100.3	101.4	100.2	101.0	109.0
B2M	11.719	1	111.4	107.6	109.4	110.2	99.9
		2	100.2	99.8	99.0	103.6	111.2
		3	105.0	99.9	107.6	110.0	119.9
C9	2.930	1	93.8	92.5	92.7	92.1	84.6
		2	103.2	94.2	102.0	102.0	111.1
		3	97.8	97.2	89.5	95.3	93.5
CA1	1.465	1	97.3	96.9	98.1	97.7	96.4
		2	93.4	94.8	87.0	97.1	98.3
		3	99.7	103.4	102.3	99.2	80.1
CALR	5.859	1	111.8	113.5	109.7	111.8	115.2
		2	105.9	**78.5**	97.7	108.8	101.0
		3	108.6	111.9	111.2	100.0	105.0
DES	1.465	1	118.0	115.6	**120.9**	111.2	113.9
		2	113.1	115.2	111.1	89.5	106.6
		3	105.3	**121.0**	90.2	100.7	118.2

F13A1	93.750	1	103.8	101.5	101.3	101.3	80.4
		2	97.6	111.6	100.2	100.2	108.0
		3	97.7	104.3	97.2	97.2	90.0
FGA	11.719	1	96.6	88.7	97.4	100.0	82.9
		2	99.1	96.6	95.6	97.5	90.6
		3	101.4	105.6	108.6	105.3	111.0
FN1	11.719	1	111.4	113.8	94.3	86.8	115.0
		2	86.4	87.6	95.6	86.5	114.5
		3	105.1	105.7	101.9	106.1	104.4
LAMP2	46.875	1	85.2	92.6	92.0	96.8	91.2
		2	92.0	100.8	97.6	91.1	84.0
		3	94.5	90.8	93.2	92.7	83.9
MTDH	5.859	1	113.5	105.9	105.3	103.1	108.3
		2	102.0	102.8	101.3	97.2	107.7
		3	106.7	99.7	100.7	96.7	99.5
ORM1	5.859	1	97.0	105.9	102.9	106.0	92.2
		2	86.5	104.1	106.3	111.0	85.2
		3	89.9	113.6	104.5	101.2	101.6
RBP4	5.859	1	101.5	98.6	100.7	100.7	116.0
		2	96.1	95.6	105.4	100.4	113.7
		3	100.8	106.0	105.5	103.4	113.6
TF	46.875	1	95.8	97.8	89.9	96.2	101.5
		2	92.1	89.9	87.8	92.8	113.9
		3	91.8	105.9	100.3	98.0	92.8
